# **Pinkment**: a synthetic platform for the development of fluorescent probes for diagnostic and theranostic applications†

**DOI:** 10.1039/d0sc02438d

**Published:** 2020-08-06

**Authors:** Maria Weber, Hai-Hao Han, Bo-Han Li, Maria L. Odyniec, Charlotte E. F. Jarman, Yi Zang, Steven D. Bull, Amanda B. Mackenzie, Adam C. Sedgwick, Jia Li, Xiao-Peng He, Tony D. James

**Affiliations:** Department of Chemistry, University of Bath Bath BA2 7AY UK t.d.james@bath.ac.uk; Centre for Doctoral Training, Centre for Sustainable & Circular Technologies, University of Bath Bath BA2 7AY UK; National Centre for Drug Screening, State Key Laboratory of Drug Research, Shanghai Institute of Materia Medica, Chinese Academy of Sciences 189 Guo Shoujing Rd. Shanghai 201203 PR China jli@simm.ac.cn; Key Laboratory for Advanced Materials & Feringa Nobel Prize Scientist Joint Research Centre, School of Chemistry and Molecular Engineering, Frontiers Center for Materiobiology and Dynamic Chemistry, East China University of Science and Technology 130 Meilong Rd. Shanghai 200237 PR China xphe@ecust.edu.cn; University of Chinese Academy of Sciences No. 19A Yuquan Road Beijing 100049 PR China; Department of Chemistry, University of Texas at Austin 105 East 24th Street A5300 Austin Texas 78712-1224 USA a.c.sedgwick@utexas.edu; Department of Pharmacy and Pharmacology, University of Bath Bath BA2 7AY UK; Centre for Therapeutic Innovation, University of Bath Bath BA2 7AY UK

## Abstract

Reaction-based fluorescent-probes have proven successful for the visualisation of biological species in various cellular processes. Unfortunately, in order to tailor the design of a fluorescent probe to a specific application (*i.e.* organelle targeting, material and theranostic applications) often requires extensive synthetic efforts and the synthetic screening of a range of fluorophores to match the required synthetic needs. In this work, we have identified **Pinkment-OH** as a unique “plug-and-play” synthetic platform that can be used to develop a range of ONOO^−^ responsive fluorescent probes for a variety of applications. These include theranostic-based applications and potential material-based/bioconjugation applications. The as prepared probes displayed an excellent sensitivity and selectivity for ONOO^−^ over other ROS. *In vitro* studies using HeLa cells and RAW 264.7 macrophages demonstrated their ability to detect exogenously and endogenously produced ONOO^−^. Evaluation in an LPS-induced inflammation mouse model illustrated the ability to monitor ONOO^−^ production in acute inflammation. Lastly, theranostic-based probes enabled the simultaneous evaluation of indomethacin-based therapeutic effects combined with the visualisation of an inflammation biomarker in RAW 264.7 cells.

## Abstract 2

**Pinkment**, a resorufin based ONOO^−^ selective and sensitive ‘plug and play’ fluorescence-based platform for *in vitro* and *in vivo* use, enables facile functionalisation for various imaging and theranostic applications.

## Graphical abstract

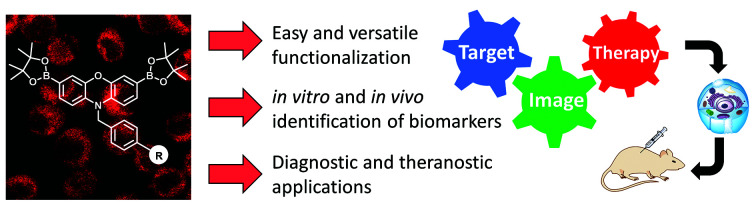

## Introduction

**Fig. 1:**
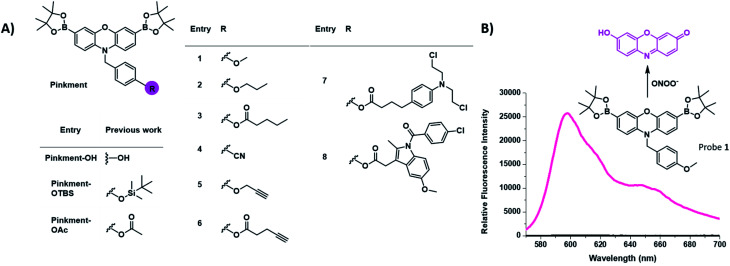
(A) Chemical structure of the resorufin-based probes for the sensing of ONOO^−^, including the previously reported **Pinkment-OH** “plug and play” scaffold and dual analyte probes **Pinkment-OTBS** and **Pinkment-OAc**.^10^ (B) Unexpected fluorescence turn on response of **1** in the presence of ONOO^−^.

There is a growing need for new and effective diagnostic tools that can evaluate biomarkers involved in inflammatory based diseases.^1–6^ Inflammation is the innate defence mechanism of the body that recognises damaged cells, pathogens and infections. The inflammatory response often results in the generation of reactive oxygen species/reactive nitrogen species (ROS/RNS), which are involved in the functional regulation of M1 and M2 macrophages.^7,8^ The M1 pro-inflammatory phenotype is induced by lipopolysaccharide (LPS), which triggers the generation of ROS from NADPH using NADPH oxidase (NOX).^9^ This production of ROS regulates an array of cellular events including the activation of the nuclear factor kappa-B (NF-κB), the production of cytokines and cell survival whereas, high levels of ROS are associated with programmed cell death, *i.e.* apoptosis.^7,10–14^ The high sensitivity and high spatial and temporal resolution of fluorescent probes allow us to visualise these key cellular events. Our group and others have focused on the fluorescence-based detection of ROS/RNS such as ONOO^−^, H_2_O_2_ and HOCl.^1,15–21^ To achieve the selective detection of a particular ROS requires the careful consideration of both fluorophore and reactive motif. In this regard, resorufin is a particularly attractive fluorophore due to its red shifted fluorescence and easy to functionalise scaffold. Pioneering work led by Chang *et al.* developed peroxyresorufin-1 (PR1) for H_2_O_2_ detection whereby resorufin is masked with boronic esters.^22,23^ Boronic esters have been identified as a relevant sensing group for both H_2_O_2_ and ONOO^−^ detection. However, in an environment with both species present, boronic esters preferentially react with ONOO^−^ due to the inherent faster reactivity of ONOO^−^ in comparison to H_2_O_2_.^24^ Previously, we have demonstrated PR1's ability to preferentially detect ONOO^−^ over H_2_O_2_*in vitro*.^25^ Consequently, we decided to investigate functionalized synthetic derivatives of PR1.^26^ This led to the development of **Pinkment-OH** for the design of dual analyte AND-logic probes, **Pinkment-OTBS** (ONOO^−^ “AND” fluoride) and **Pinkment-OAc** (H_2_O_2_ “AND” esterase) using **Pinkment-OH** (Fig. 1) as a synthetic starting point. These results revealed the potential of **Pinkment-OH** to be used as a synthetic platform for the development of ONOO^−^ selective fluorescence probes with additional sensing, targeting or drug units. Here, we have serendipitously discovered that the benzyl unit of our ROS **Pinkment** fluorescent probe can be functionalized with a functional unit of choice without compromising ROS selectivity. As a result, **Pinkment-OH** was successfully shown as a synthetic platform to develop ONOO^−^ selective fluorescent probes with additional functional units (Fig. 1).

## Results and discussion

Initially, our focus was on continuing the development of “AND”-based logic-gates for biological application.^1,17,26^ This led to the elaboration of probe **1**, which was accessed in a simple three step synthesis (Scheme S1†). Unexpectedly, we discovered that **1** “turned on” in the sole presence of ONOO^−^ (Fig. 1B and S1†). This led to the development of **Pinkment** probes **2** and **3** to further confirm this observation. These probes were accessible from the synthetic platform **Pinkment-OH**, whose 6-step synthesis has been previously reported by our group.^26^ Nucleophilic substitution by **Pinkment-OH** using 1-bromopropane and pentanoyl chloride respectively gave **2** and **3** in moderate yields: 50% and 51% respectively (Scheme S2 and S3†). Probes **2** and **3** showed good selectivity towards ONOO^−^ over other ROS species (Fig. S2–S8†). Surprisingly, the probes demonstrated a high sensitivity towards ONOO^−^ requiring concentrations in the low micromolar range. Both **2** and **3** displayed increased solubility in comparison to **1**. We decided to further explore this unexpected result by introducing a terminal nitrile group. Probe **4** was accessible in a facile three-step synthesis (Scheme S4†) in the same manner as **1**. Again, good selectivity and sensitivity for ONOO^−^ was observed (Fig. S2–S8†). From these results, we realized that the **Pinkment** benzyl unit can be functionalized with any unit of choice without compromising the ROS selectivity. Thus, we rationalized that **Pinkment-OH** offers a unique platform for the design of ONOO^−^ selective fluorescence based probes that can be tailored towards a range of applications.^27–29^ This led to the development of alkyne-based **Pinkment** probes **5** and **6** that have potential to be used in “click” chemistry.^29^ These probes were accessed from **Pinkment-OH** and prepared in moderate yields: 48% and 47% for **5**, and **6** respectively (Scheme S5 and S6†). Fluorescence studies of **5** and **6** established good sensitivity and selectivity towards ONOO^−^ over other ROS (Fig. S2–S8†).

We then turned our attention to assessing the imaging capacity of probes **2** and **3** and the potential “click” based probes **5** and **6** in cells and live animals. To demonstrate their suitability as imaging tools, all four probes were evaluated for cellular toxicity in murine RAW 264.7 macrophages using a MTS cell proliferation assay. Probes **2**, **3**, **5** and **6** were incubated at different concentrations ranging from 5 to 40 μM for 24 h (Fig. S9†). Probes **2**, **5** and **6** were found to be non-toxic. In contrast, probe **3** decreased the cell viability of RAW 264.7 macrophages by 40% at a concentration of 40 μM compared to control conditions. As a result, **3** was not taken forward for further cell studies since high concentrations of the probe are required for *in vivo* studies.

**Fig. 2:**
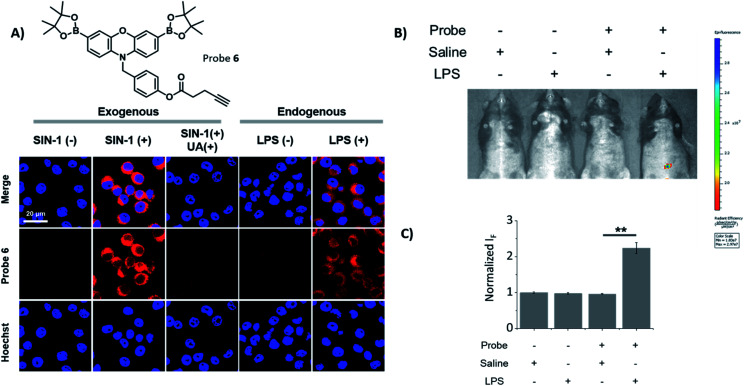
(A) Confocal imaging of RAW 264.7 macrophages treated with probe **6** (20 μM, 30 min) in the presence and absence of SIN-1 (500 μM, 30 min) and uric acid (100 μM, 2 h) or LPS (1 μg mL^−1^, 24 h) as indicated. Fluorescence data was collected using *λ*_ex_ = 559 nm and *λ*_em_ = 580–650 nm, respectively. The cell nuclei was stained using Hoechst 33342 and fluorescence collected at *λ*_ex_ = 405 nm and *λ*_em_ = 450–480 nm. Scale bar = 20 μm. *N* = 3. (B) Intraperitoneal injection of male C57BL/6J mice with probe **6** (200 μM) or saline in the absence and presence of LPS (2 mg mL^−1^ in saline) with *λ*_ex_ = 535 nm and *λ*_em_ = 600 nm. *N* = 3. (C) Quantification of (B) C57BL/6J male mice treated with probe **6** (200 μM) or saline in the absence and presence of LPS (2 mg mL^−1^ in saline) with *λ*_ex_ = 550 nm and *λ*_em_ = 580–620 nm. Error bars represent s. d. with **p ≤ 0.01. *N* = 3. Normalised fluorescence intensities were calculated using the saline solution fluorescence intensities.

Probes **2**, **5** and **6** were shown to be non-toxic, and were evaluated with exogenous ONOO^−^, using SIN-1 (500 μM) in RAW 264.7 macrophages (Fig. 2A and S10†). Each probe alone demonstrated minimal fluorescence in cells, the addition of SIN-1 led to a significant enhancement in intracellular fluorescence at a wavelength corresponding to the dye, resorufin, therefore, suggesting the intracellular reaction of the probe with ONOO^−^ and their suitability for use as fluorescence-based probes. The SIN-1 generated fluorescence signal was then evaluated with the ONOO^−^ scavenger, uric acid.^30^ As expected, uric acid attenuated the fluorescent increase that was induced by SIN-1 for all probes, thus confirming the ONOO^−^ mediated increase in fluorescence intensity. Next, we evaluated the capability of **2**, **5** and **6** to detect endogenous ONOO^−^ in LPS primed RAW 264.7 macrophages. All three probes were shown to detect endogenous ONOO^−^ in LPS primed RAW 264.7 macrophages (Fig. 2A and S11†), confirming their promise for the imaging of LPS-induced inflammatory responses. In addition, HeLa and A549 cell lines treated with or without SIN-1 were used to illustrate the versatility of the **Pinkment** probes (Fig. S12 and S13†).

Encouraged by these cell imaging results, we used a known LPS-induced inflammation mouse model^31^ for the *in vivo* detection of ONOO^−^ (Fig. 2B). The injection of LPS (2 mg mL^−1^ in saline) to the abdominal region of mice followed by the injection of **6** (200 μM) led to its fluorescence activation. The quantified fluorescence intensity in the probe(+)/LPS(+) group was significantly larger than that in the probe(+)/LPS(−) group (Fig. 1C), demonstrating the potential of using **6** for the monitoring of ONOO^−^*in situ* during acute inflammation.

In order to follow our current interest in theranostics,^32^ we then turned our attention towards the potential of **Pinkment-OH** for the design of fluorescence-based drug releasing probes. Therefore, we used the drugs chlorambucil and indomethacin to afford two distinct theranostic probes **7** and **8**, respectively (Fig. 1A). Chlorambucil is used to treat chronic lymphatic leukemia^33^ and indomethacin is used as a non-steroidal anti-inflammatory drug (NSAID).^34,35^ Both **7** and **8** were easily accessible from **Pinkment-OH** (Scheme S7 and S8†).

Mass spectrometry confirmed and validated the simultaneous release of each drug and fluorescent resorufin dye (Fig. S14 and S15†). Therefore, the enhancement in the fluorescence intensity over time indicates the release of each drug. As such, time-dependent fluorescence experiments with **7** and **8** in the presence of ONOO^−^ were performed to illustrate the time dependence of the drug release. These experiments revealed a maximum fluorescence response after ∼10 min (Fig. S16†).

**Fig. 3:**
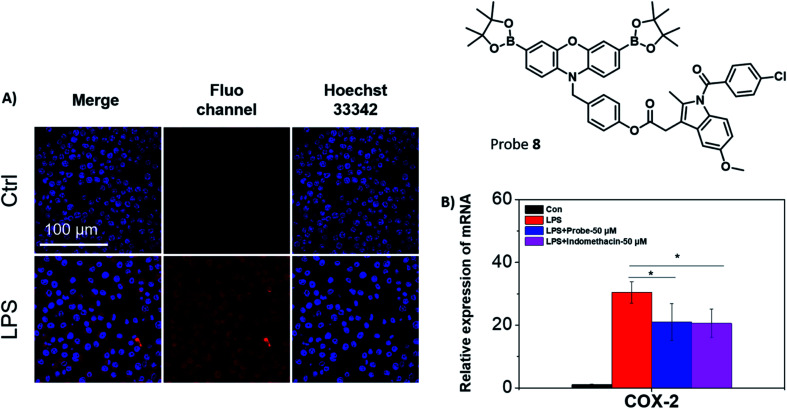
(A) Confocal imaging of RAW 264.7 macrophages treated with LPS (1 μg mL^−1^, 24 h) and then loaded with **8** (20 μM, 30 min) as indicated. Fluorescence data was collected using *λ*_ex_ = 559 nm and *λ*_em_ = 580–650 nm, respectively. The cell nuclei were stained using Hoechst 33342 and fluorescence collected at *λ*_ex_ = 405 nm and *λ*_em_ = 450–480 nm. Scale bar = 100 μm. *N* = 3. (B) Effect of **8** on LPS-induced COX-2 gene expression in RAW 264.7 macrophages. Cells were treated with LPS alone (1 μg mL^−1^) or together with **8** for 24 h. Indomethacin was set as a positive control, and the relative mRNA level of COX-2 gene was normalized by GAPDH (*p < 0.05). *N* = 4.

Fluorescence studies were carried out including ROS selectivity, H_2_O_2_ titration and ONOO^−^ titration studies (Fig. S17–S21†) and demonstrated high sensitivity towards these inflammation-based biomarkers. Following these initial studies, we evaluated both **7** and **8** in RAW 264.7 macrophages towards exogenous ONOO^−^ detection (Fig. S22†). The presence of SIN-1 significantly enhanced the intracellular fluorescence of **7** and **8**, confirming the applicability of the probes *in vitro*. Despite **7** displaying significant promise, the creation of an appropriate model system to differentiate between cancerous and healthy cells would require a significant amount of development and as such was beyond the scope of this current research. Therefore, only **8** was further evaluated, since its cellular behaviour was easier to monitor. Endogenous ONOO^−^ was also detected by **8** in RAW 264.7 macrophages (Fig. 3A). Indomethacin, a NSAID, is an effective and non-selective inhibitor of cyclooxygenase-1 (COX-1) and cyclooxygenase-2 (COX-2), of which COX-2 is mainly responsible for the inflammatory response.^36^ The therapeutic effects on the LPS-induced inflammatory responses in RAW 264.7 macrophages were further investigated using **8**. RAW 264.7 macrophages were treated with LPS and the expression of the pro-inflammatory gene (COX-2) was investigated using qRT-PCR in the presence or absence of **8** (Fig. 3B).^37^ The mRNA level of COX-2 decreased in the presence of **8** (50 μM) in comparison to the LPS-induced group. A similar effect to the LPS-induced group was observed with indomethacin alone. This suggests that **8** can monitor ONOO^−^ production in acute inflammation, and in addition, reduce the inflammatory response by releasing indomethacin.

## Conclusions

The ability of the **Pinkment** scaffold to be functionalised with any unit of choice without compromising the overall ROS selectivity, opens up new possibilities for the design of highly specific ONOO^−^ probes that can be used in a variety of applications. In this work, we have successfully illustrated the applicability of **Pinkment**-based probes for diagnostic and theranostics applications. Our probes displayed good selectivity and sensitivity towards ONOO^−^ over a range of other ROS. Cellular studies with the **Pinkment** probes led to the identification of alkyne-functionalised **Pinkment** probe **6** as a suitable candidate for *in vivo* studies using an inflammatory mouse model. These promising results led us to design potential theranostic probes **7** and **8** with candidate **8** displaying promising properties *in vitro*. We believe this work demonstrates **Pinkment-OH** as a useful synthetic platform to enable the rapid development of a ONOO^−^ fluorescent probe that can be tailored to the needs of the chemical biologist. In particular, the alkyne **Pinkment** probes offer the possibility of attaching any desired unit *via* click chemistry. Therefore, we anticipate that the **Pinkment** scaffold can be further elaborated for the development of dual analyte, organelle targeting and theranostic probes for a range of diagnostic and theranostic applications.

## Conflicts of interest

There are no conflicts to declare.

## Supplementary Material

SC-011-D0SC02438D-s001
