# Supplementary material for: Pinkment: a synthetic platform for the development of fluorescent probes for diagnostic and theranostic applications
Source: Chem Sci. 2020 Aug 6;11(32):8567–71. doi: 10.1039/d0sc02438d (PMC8163375; doi:10.1039/d0sc02438d)
Supplement: SC-011-D0SC02438D-s001 [file SC-011-D0SC02438D-s001.pdf]

Electronic Supplementary Material (ESI) for Chemical Science.
This journal is © The Royal Society of Chemistry 2020

Pinkment: A Synthetic Platform for the Development of Fluorescent Probes for Diagnostic and Theranostic Applications

Maria Weber,[a,1,2] Hai-Hao Han,[a, 3, 4] Bo-Han Li,[a, 3, 5] Maria L. Odyniec,[1] Charlotte E. F. Jarman,[1] Yi Zang,[3] Steven D. Bull,[1] Amanda B. Mackenzie,[6, 7] Adam C. Sedgwick,[8,*] Jia Li,[3,5*] Xiao-Peng He[4,*] and Tony D. James[1,*]

[1]Department of Chemistry, University of Bath, Bath, BA2 7AY, UK
[2]Centre for Doctoral Training, Centre for Sustainable & Circular Technologies, University of Bath, Bath, BA2 7AY, UK
[3]National Center for Drug Screening, State Key Laboratory of Drug Research, Shanghai Institute of Materia Medica, Chinese Academy of Sciences, 189 Guo Shoujing Rd., Shanghai 201203, P. R. China
[4] Key Laboratory for Advanced Materials & Feringa Nobel Prize Scientist Joint Research Centre, School of Chemistry and Molecular Engineering, Frontiers Center for Materiobiology and Dynamic Chemistry, East China University of Science and Technology, 130 Meilong Rd., Shanghai 200237, PR China.
[5] University of Chinese Academy of Sciences, No.19A Yuquan Road, Beijing 100049, PR China
[6] Department of Pharmacy and Pharmacology, University of Bath, Bath, BA2 7AY, UK
[7] Centre for Therapeutic Innovation, University of Bath, Bath, BA2 7AY, UK
[8] Department of Chemistry. University of Texas at Austin, 105 East 24th Street A5300, Austin, Texas 78712-1224, United States

[a]Equal contribution

Email:
t.d.james@bath.ac.uk (T.D.J.)
xphe@ecust.edu.cn (X.P.H.)
a.c.sedgwick@utexas.edu (A.C.S)
jli@simm.ac.cn (J.L.)

Table of Contents

1. GENERAL MATERIALS AND METHODS S3

1.1. CHEMISTRY S3

1.2. FLUORESCENCE S4

1.3. *IN VITRO* STUDIES S5

1.3.1. CELL CULTURE S5

1.3.2. CELL VIABILITY ASSAY S5

1.3.3. RNA ISOLATION AND QUANTITATIVE RT-PCR. S5

1.3.4. CONFOCAL LASER SCANNING MICROSCOPY S5

1.4. *IN VIVO* STUDIES S6

2. SYNTHESIS S7

3. SCHEMES AND FIGURES S17

5. NMR SPECTRA S39

AUTHOR CONTRIBUTIONS S52

1. General Materials and Methods

1.1. Chemistry

Unless stated otherwise, reagents and solvents were sourced from commercial suppliers, specifically: Acros Organics, Fisher Scientific, Fluka Chemie GmbH, Fluorochem, Sigma Aldrich and TCI and were used directly as received. Reactions requiring inert conditions were performed using dry solvents and under an atmosphere of nitrogen. Solvents were dried using an Innovative Technology Inc. PS-400-7 Solvent Purification System.

TLC was carried out on commercially available pre-coated aluminium-backed silica plates and compounds were visualised under UV light at 254 nm. Column chromatography was performed using 60 micron silica purchased from Sigma Aldrich.

^{1}H NMR and ^{13}C NMR spectra were recorded either in deuterated chloroform, acetone, methanol or DMSO at ambient temperature on either a Bruker Avance 250 (250 MHz), Bruker Avance 300 (300 MHz) or Bruker Avance 500 (500 MHz), with proton decoupling for all ^{13}C NMR spectra. Chemical shifts (δ / ppm) are referenced against tetramethylsilane as an internal standard and the abbreviations: s, d, t, q, quin, sext, m, and br, were used to denote singlet, doublet, triplet, quartet, quintet, sextet, multiplet, and broad respectively. Coupling constants (*J* / Hz) are reported where known.

Initially, analysis by mass spectrometry was conducted either by the EPSRC UK national mass spectrometry facility at Swansea University medical school on an electrospray ionisation LTQ Orbitrap XL 1 (Thermo Fisher Scientific) or on a microTOF electrospray time of flight (ESI-TOF) mass spectrometer (Bruker Dlatonik GmbH, Germany) at the University of Bath. More recently, LC-MS analyses were performed using an Agilent QTOF 6545 with Jetstream ESI spray source coupled to an Agilent 1260 Infinity II Quat pump HPLC with 1260 autosampler, column oven compartment and variable wavelength detector (VWD). The MS was operated in either positive or negative ionisation mode with the gas temperature at 250 °C, the drying gas at 12 L/min and the nebuliser gas at 45 psi (3.10 bar). The sheath gas temperature and flow were set to 350 °C and 12 L/min, respectively. The MS was calibrated using reference calibrant introduced from the independent ESI reference sprayer. The VCap, Fragmentor and Skimmer were set to 3500, 125 and 45 respectively.

For more advanced mass spectrometry analysis, the following guidelines were followed: Flow infusion analysis was conducted using a Maxis HD quadrupole electrospray time-of-flight (ESI-QTOF) mass spectrometer (Bruker Daltonik GmbH, Bremen, Germany) in negative-ion mode. The capillary voltage was set to 4500 V, nebulising gas at 1 bar, drying gas at 4 L/min. The drying gas temperature was increased from 200 – 280 °C during the first infusion. For the second infusion the freshly prepared sample was run at 280 °C, then increased to 320 °C. Extracted MS spectra represents drying gas conditions of 280 °C. The TOF scan range was from 50 – 1000 mass-to-charge ratio (*m/z*). Infusion injections were performed by the infusion pump using a flow rate of 3 µL/min. The MS instrument was calibrated using a sodium formate calibrant solution. The calibrant solution consisted of 3 parts of 1 M NaOH to 97 parts of 50 : 50 water : isopropanol with 2 % formic acid. Data processing was performed using the Compass Data Analysis software version 4.3 (Bruker Daltonik GmbH, Bremen, Germany).

Fourier Transform Infrared (FTIR) spectra were recorded on an ATR Perkin−Elmer FTIR Spectrum 100

spectrometer. The spectra were measured between 4000 − 600 cm^{-1}. Selected absorption bands are reported in wavenumbers (cm^{-1}), and their relative intensities described as *s* (strong), *m* (medium), or *w* (weak). When applicable, peak shape was characterised by *br.* (broad) or *sh.* (shoulder).

1.2. Fluorescence

Fluorescence measurements were performed on a BMG Labtech CLARIOstar® plate reader using Greiner bio-one microplates, 96-well, PS, flat-bottom (chimney well), black. Data were collected via the BMG Labtech Clariostar data analysis software package MARS. All solvents used in fluorescence measurements were HPLC or fluorescence grade and the water was de-ionised.
UV-Vis measurements were performed on a Perkin-Elmer Lambda20 Spectrophotometer, utilising Starna Silica (quartz) cuvette with 10 mm path lengths, two faces polished. Data was collected *via* the Perkin-Elmer UVWinlab software package.
All pH measurements taken for buffer pH adjustments were recorded on a Hanna Instrument HI 9321 microprocessor pH meter which was routinely calibrated using Fishers standard buffer solutions (pH 4.0 – phthalate, 7.0 – phosphate, and 10.0 – borate).
Phosphate buffered saline (PBS) was freshly prepared from 52 % methanol in water with KCl (10 mM), KH_2PO_4 (2.75 mM) and Na_2HPO_4 (2.76 mM). The PBS buffer was adjusted to pH 7.4/8.2 with 1 M HCl (aq.) as indicated.

Stock solutions for a variety of ROS were freshly prepared prior to each experiment:
<u>Hydrogen peroxide (H_2O_2)</u>
Hydrogen peroxide (H_2O_2) is commercially available whereby the concentration of H_2O_2 was determined through spectrophotometrical analysis with ε = 43.6 cm^{-1} M^{-1} at 240 nm.
<u>Peroxynitrite ($ONOO^-$)</u>
Peroxynitrite ($ONOO^-$) stock solutions were freshly prepared each time prior to usage. A solution of 3 M NaOH was cooled to 0 °C to which simultaneously 0.7 M H_2O_2, 0.6 M $NaNO_2$ and 0.6 M HCl were added. The $ONOO^-$ solution was analyzed spectrophotometrically whereby the concentration of $ONOO^-$ was estimated through ε = 1670 cm^{-1} M^{-1} at 302 nm in 0.1 M NaOH (aq.).
<u>Hypochlorite (ClO^-)</u>
Commercially available NaOCl was analysed spectrophotometrically to determine its concentration using ε = 350 cm^{-1} M^{-1} at 292 nm.
<u>Superoxide ($O_2^{\cdot-}$)</u>
KO_2 (0.0335 g) and 18-crown-6 ether (0.1235 g) were dissolved in DMSO (5 mL) and stirred for 5 min to produce a 0.1 M solution of $O_2^{\cdot-}$.
<u>Hydroxyl radical ($^{\cdot}OH$)</u>
Iron(II) perchlorate hydrate (0.1274 g) and H_2O_2 (3.9 µL) were dissolved in H_2O (5 mL) to produce a 0.1 M solution of $^{\cdot}OH$.
<u>Peroxyl radical ($ROO^{\cdot}$)</u>
2,2′-Azobis(2-methylpropionamidine) dihydrochloride (0.136 g) was dissolved in H_2O (5 mL) and heated at 37 °C for 30 min to give a 0.1 M solution of $ROO^{\cdot}$.
<u>Singlet oxygen ($^1O_2$)</u>
H_2O_2 (1.3 µL) was added to 4 mL of 0.1 M NaOCl to give a 0.1M solution of $^1O_2$.

Fluorescence titrations of ROS/RNS were carried out at 25 °C in PBS buffer pH 7.4/8.2. Different concentrations of ROS/RNS were prepared accordingly and investigated with the probe at a concentration ranging from 500 nM to 5 µM.

1.3. *In vitro* Studies

1.3.1. Cell Culture

RAW 264.7 macrophages (ATCC® TIB-71™) and HeLa cells(ATCC® CCL-2™) were maintained in a Dulbecco's Modified Eagle's Medium (DMEM, Gibco,12800082) supplemented with 10 % FBS (Gibco, 2025790) in a humidified atmosphere of 5 % CO_2 and 95 % air at 37 °C and split when the cells reached 90 % confluency.

1.3.2. Cell Viability Assay

Cells were plated on clear bottom 96-well plates (Corning, 3599) in growth medium. After 24 h, cells were treated with probes at different concentrations (0, 5, 10, 20, and 40 µM) for 24 h. Then, a solution of MTS/PMS (20:1, Promega Corp, G5430) (10 µL/well) was added to each well. After incubation at 37 °C under 5 % CO_2 for 2 h, the absorbance of the solutions was measured at 490 nm using a M5 microplate reader (Molecular Device, USA). The optical density of the result in the MTS assay was directly proportional to the number of viable cells.

1.3.3. RNA isolation and quantitative RT-PCR.

Total RNA was isolated from RAW 264.7 macrophages treated with or without different reagents using TRIzol reagent (Invitrogen, 15596018). The cDNA was prepared using PrimeScript Reverse Transcriptase (TaKaRa, RR036A) according to the manufacturer's instructions. After a 10-fold dilution, the gene expression was amplified using 2x SYBR Green qPCR Master Mix (ABclonal, RK21203) with a Stratagene Mx3005P (Agilent Technologies). qPCR values were normalized to GAPDH. Statistical analysis was performed with GraphPad PRISM (GraphPad Software, Inc.) using Student's unpaired t-test.The following primer sequences were synthetized from Sangon Biotech (Shanghai) Co., Ltd.:
GAPDH: 5'-TCTCCTGCGACTTCAACA-3'(forward),
5'-TGGTCCAGGGTTTCTTACT-3' (reverse);
cyclooxygenase(COX)-2: 5'-CTGGAACATGGACTCACTCAGTTTG-3' (forward),
5'-AGGCCTTTGCCACTGCTTGT-3' (reverse)

1.3.4. Confocal Laser Scanning Microscopy

HeLa and RAW 264.7 macrophages were plated onto 24-well plates (Corning, 3524) in DMEM plus 10 % FBS. The cells were cultured overnight at 37 °C in a humidified atmosphere of 5 % CO_2 and 95 % air. First, cells were incubated with an $ONOO^-$ donor (SIN-1) to evaluate sensitivity to exogenous $ONOO^-$. Cells were incubated with the probe (20 µM, 1% DMSO in PBS, pH 7.4) for 30 min, followed by incubation with SIN-1 (500 µM) for 30 min at 37 °C. To confirm the detection of exogenously generated $ONOO^-$, the cells were pre-incubated with uric acid (100 µM) for 2 h and then SIN-1 (500 µM) for 30 min followed by the probe (20 µM) for 30 min at 37 °C. To generate endogenous $ONOO^-$, LPS (1 µg mL^{-1}) was first incubated for 24 h in RAW 264.7 macrophages at 37 °C. After washing the LPS-primed cells with PBS twice, the cells were incubated with the probe (20 µM) for 30 min at 37 °C. The cells were then washed three times with PBS and fixed by 4 % paraformaldehyde for 20 min at RT. After rinsing twice with PBS, the nuclei of the fixed cells were stained with Hoechst 33342 (5 µg

mL^{-1}) at 37 °C in a humidified atmosphere of 5 % CO_2 in air for 5 min. The cells were then washed three times with PBS, the fluorescent signals were analysed using a confocal laser scanning microscopy (FV1000, Olympus, Japan) with λ_{ex} = 559 nm and λ_{em} = 580-650 nm for probes and λ_{ex} = 405 nm and λ_{em} = 450-480 nm for Hoechst 33342. Fluorescent cell images were analyzed and exported via FV10-ASW software (Olympus).

1.4. *In vivo* Studies

All animal experiments were carried out under the Guidelines for Care and Use of Laboratory Animals of Shanghai Institute of Materia Medica (SIMM), Chinese Academy of Sciences and approved by the Institutional Animal Care and Use Committee (IACUC) of SIMM (Shanghai, China). In addition, the procedures performed at SIMM were approved (NL1907-1) by the Animal Welfare & Ethical Review Body (AWERB) at the University of Bath.

Animals were housed in a SPF temperature-controlled room (22 ± 2°C) under a 12 h light/dark cycle in the Shanghai Institute of Materia Medica (SIMM) of the Chinese Academy of Science. Animals were anesthetized with pentobarbital sodium (50 mg/kg). At the end of the study, the mice were euthanized with CO_2 asphyxiation without recovery from the anaesthesia, according to the rules of the Animal Care and Use Committee of the Shanghai Institute of Materia Medica. Mice were divided into four groups and three mice were used in each group from three independent experiments. In addition, four mice were used for preliminary experiments to explore conditions.

8-week-old male C57BL/6J mice were divided into two groups; the first group was given an intraperitoneal (i.p.) injection of saline (200 µL) as a control group, and the second group was injected i.p. with LPS (200 µL, 2 mg mL^{-1} in saline, Sigma Aldrich, L2630). After 4 h, animals were anesthetized and abdominal fur was removed using a razor. Then, mice were injected i.p. with the probe (100 µL, 200 µM in saline) or saline (100 µL). Whole body images were acquired in 30 min by using the IVIS spectrum imaging system (PerkinElmer, USA). The imaging mode was set as: λ_{ex} = 550 nm, λ_{em} = 580-620 nm. Quantitative analysis of data was carried out with the Living Image 4.0 software where the average signal intensity using the saline group was normalized to 1. Statistical analysis was performed with GraphPad PRISM (GraphPad Software, Inc.) using Student's unpaired t-test. P-values <0.05 was considered statistically significant.

2. Synthesis

10-(4-methoxybenzyl)-10*H*-phenoxazine

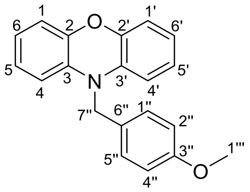

Phenoxazine (1.00 g, 5.46 mmol) and NaH, 60 % in mineral oil (0.26 g, 10.90 mmol) were added to dry DMF (30 mL) and cooled to 0 ºC. After stirring for 10 min, 4-methoxybenzyl chloride (0.85 g, 5.45 mmol) in DMF (10 mL) was added dropwise to the reaction mixture and left to stir at RT for 4 h. The reaction mixture was then quenched via dropwise addition of H_2O. The mixture was partitioned with EtOAc (100 mL) and H_2O (100 mL). The organic layer was washed with H_2O (3 x 50 mL) and brine (1 x 50 mL), dried over $MgSO_4$, filtered and concentrated *in-vacuo* to afford 10-(4-methoxybenzyl)-10*H*-phenoxazine (0.72 g, 43 %) as a brown solid.

^{1}H NMR (300 MHz, C*D*Cl$_3$): δ = 7.25 – 7.20 (m, 2 H; Ar*H*), 6.90 – 6.85 (d, *J* = 8.7 Hz 2 H; Ar*H*), 6.76 – 6.64 (t, *J* = 1.4 Hz, 6 H; Ar*H*), 6.36 – 6.34 (d, *J* = 7.2, 1.3 Hz, 2 H; Ar*H*), 4.73 (s, 2 H; H–C(7’’)), 3.79 ppm (s, 3 H; H–C(1’’)); ^{13}C NMR (75 MHz, C*D*Cl$_3$): δ = 158.73, 145.15, 133.88, 127.99, 127.15, 123.71, 121.15, 115.23, 114.35, 112.19, 55.31, 48.70 ppm; m.p. 119 – 121 ºC; IR (ATR): υ = 2843.1 cm^{-1} (s, O-CH$_3$); HR-ESI-MS: m/z (%): 304.1329 ([*M*+H]$^+$, calcd for $C_{20}H_{18}NO_2^+$: 304.2337).

3,7-dibromo-10-(4-methoxybenzyl)-10*H*-phenoxazine

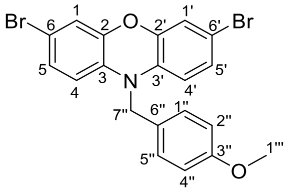

To a solution of 4-((3,7-dibromo-10*H*-phenoxazin-10-yl)methyl)methyl ester (0.67 g, 2.22 mmol) in $CHCl_3$ (100 mL), NBS (0.78g, 4.43 mmol) was added portion-wise and the reaction was left to stir for 1 h. The reaction was quenched with H_2O (100 mL) and the organic layer was washed with H_2O (3 x 50 mL) and brine (1 x 100 mL), dried over $MgSO_4$, filtered and and concentrated *in vacuo* to afford 3,7-dibromo-10-(4-methoxybenzyl)-10*H*-phenoxazine (0.91 g, 89 %) as a green solid.

^{1}H NMR (300 MHz, C*D*Cl$_3$): δ = 7.17 – 7.14 (d, *J* = 8.5 Hz, 2 H; Ar*H*), 6.89 – 6.86 (d, *J* = 8.7 Hz, 2 H; Ar*H*), 6.83 – 6.80 (d, *J* = 6.9 Hz, 4 H; Ar*H*), 6.21 – 6.18 (d, *J* = 9.1 Hz, 2 H; Ar*H*), 4.66 (s, 2 H; H–C(7’’)), 3.79 ppm (s, 3 H; H–C(1’’’)); ^{13}C NMR (75 MHz, C*D*Cl$_3$): δ = 158.98, 145.35, 132.71, 126.99, 126.75, 126.67, 118.57, 114.52, 113.33, 112.80, 55.30, 48.65 ppm; m.p. 162 –164 ºC; IR (ATR): υ = 2836.4 cm^{-1} (s, O-CH$_3$); HR-ESI-MS: m/z (%): 461.9519 ([*M*+H]$^+$, calcd for $C_{20}H_{16}NO_2{}^{79}Br^{81}Br^+$: 461.9520).

10-(4-methoxybenzyl)-3,7-bis(4,4,5,5-tetramethyl-1,3,2-dioxaborolan-2-yl)-10*H*-phenoxazine

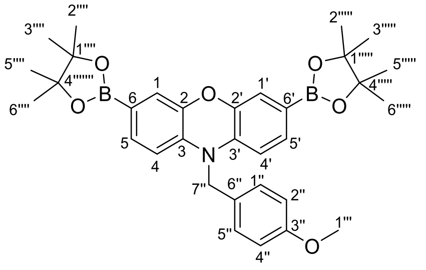

4-((3,7-bis(4,4,5,5-tetramethyl-1,3,2-dioxaborolan-2-yl)-10*H*-phenoxazin-10-yl)methyl)methyl ester (1.00 g, 2.16 mmol), bis(pinacolato)diboron (1.65 g, 6.51 mmol), KOAc (1.28 g, 13.04 mmol) and Pd(dppf)Cl_2 (0.16 g, 0.27 mmol) were dissolved in anhydrous DMF (15 mL) and refluxed under argon for 2 h. The reaction was cooled to RT and partitioned with EtOAc (50 mL) and H_2O (50 mL). The organic layer was washed with H_2O (3 x 50 mL), brine (1 x 50 mL), dried over $MgSO_4$, filtered and concentrated *in vacuo*. Trituration with petroleum ether 40-60 (100 mL) afforded 10-(4-methoxybenzyl)-3,7-bis(4,4,5,5-tetramethyl-1,3,2-dioxaborolan-2-yl)-10*H*-phenoxazine (0.41 g, 41 %) as a brown solid.

^{1}H NMR (300 MHz, C*D*Cl_3): δ = 7.17 – 7.16 (d, *J* = 3.3 Hz, 2 H; Ar*H*), 7.14 – 7.13 (d, *J* = 1.4 Hz, 2 H; Ar*H*), 7.07 – 7.06 (d, *J* = 1.4 Hz, 2 H; Ar*H*), 6.86 – 6.83 (d, *J* = 8.7 Hz, 2 H; Ar*H*), 6.34 – 6.32 (d, *J* = 8.0 Hz, 2 H; Ar*H*), 4.74 (s, 2 H; H–C(7’’)), 3.78 (s, 3 H; H–C(1’’’)), 1.30 ppm (s, 24 H; H–C(2’’’’), H–C(3’’’’), H–C(5’’’’), H–C(6’’’’), C(2’’’’’), H–C(3’’’’’), H–C(5’’’’’), H–C(6’’’’’)); ^{13}C NMR (75 MHz, C*D*Cl_3): δ = 144.72, 136.04, 130.97, 127.33, 127.12, 120.92, 114.38, 111.70, 83.60, 55.32, 25.01, 24.83, 24.61 ppm; m.p. 165 ºC; IR (ATR): υ = 2970.3 cm^{-1} (s, O-CH_3); HR-ESI-MS: m/z (%): 556.3034 ([*M*+H]$^+$, calcd for $C_{32}H_{40}NO_6{}^{10}B^{11}B^+$: 556.3047).

10-(4-propoxybenzyl)-3,7-bis(4,4,5,5-tetramethyl-1,3,2-dioxaborolan-2-yl)-10*H*-phenoxazine

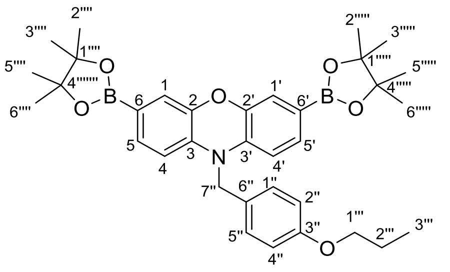

4-((3,7-bis(4,4,5,5-tetramethyl-1,3,2-dioxaborolan-2-yl)-10*H*-phenoxazin-10-yl)methyl)phenol (300 mg, 0.55 mmol) was dissolved in THF (30 mL). NaH–60 % mineral oil (32 mg, 0.83 mmol) was added, followed by dropwise addition of propargyl bromide – 80 wt% in toluene (0.15 mL, 0.83 mmol). The mixture was stirred at RT for 20 h. The reaction was quenched with MeOH (20 mL), diluted with EtOAc (30 mL), washed with water (3 x 60 mL), dried over $MgSO_4$, filtered and evaporated *in vacuo*. FC (SiO_2; petroleum ether/EtOAc 80:20) gave 10-(4-propoxybenzyl)-3,7-bis(4,4,5,5-tetramethyl-1,3,2-dioxaborolan-2-yl)-10*H*-phenoxazine (130 mg, 41 %) as a yellow solid.

^{1}H NMR (500 MHz, $CDCl_3$): δ = 7.20 – 7.11 (m, 4 H; Ar*H*), 7.10 – 7.03 (m, 2 H; Ar*H*), 6.79 (dd, *J* = 8.5, 6.1 Hz, 1 H; Ar*H*), 6.70 – 6.64 (m, 1 H; Ar*H*), 6.34 (dd, *J* = 7.9, 3.8 Hz, 2 H; Ar*H*), 4.72 (s, 2 H; H–C(7'')), 4.12 (q, *J* = 7.2 Hz, 2 H; H–C(1''')), 1.30 (s, 24 H; H–C(2''''), H–C(3''''), H–C(5''''), H–C(6''''), H–C(2'''''), H–C(3'''''), H–C(5'''''), H–C(6''''')), 1.28 – 1.23 (m, 2 H; H–C(2''')) 0.89 – 0.83 ppm (m, 3 H; H–C(3''')); ^{13}C NMR (126 MHz, $CDCl_3$): δ = 154.99, 144.73, 136.06, 130.96, 127.33, 127.30, 127.15, 120.97, 115.80, 111.73, 83.69, 48.10, 29.70, 24.78, 14.19, 1.02 ppm; m.p. 217 – 220 °C; IR (ATR): υ = 1138 cm^{-1} (*m*, C–O–C); HR-ESI-MS: m/z (%): 581.3365 ([*M*]$^+$, calcd for $C_{34}H_{43}O_6N^{10}B_2^+$: 581.3349).

4-((3,7-bis(4,4,5,5-tetramethyl-1,3,2-dioxaborolan-2-yl)-10*H*-phenoxazin-10-yl)methyl)phenyl pentanoate

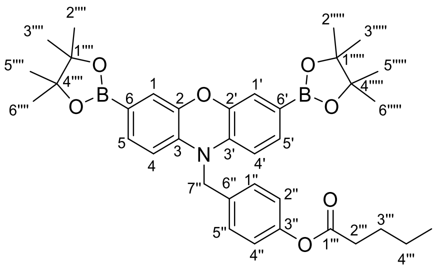

4-((3,7-bis(4,4,5,5-tetramethyl-1,3,2-dioxaborolan-2-yl)-10*H*-phenoxazin-10-yl)methyl)phenol (200 mg, 0.37 mmol) was dissolved in DCM (30 mL). The solution was cooled to 0 °C. First triethylamine (0.10 ml, 0.74 mmol) was added, followed by dropwise addition of valeroyl chloride (0.1 ml, 0.74 mmol). The mixture was stirred for 4 h at RT. The reaction was quenched with water, and the phases were separated. The organic phase was washed with water (3 x 20 ml), dried over $MgSO_4$, filtered and evaporated *in vacuo*. FC (SiO_2; petroleum ether/EtOAc 80:20) gave 4-((3,7-bis(4,4,5,5-tetramethyl-1,3,2-dioxaborolan-2-yl)-10*H*-phenoxazin-10-yl)methyl) phenyl pentanoate (72 mg, 31%) as a yellow/orange solid.

^{1}H NMR (500 MHz, C*D*Cl$_3$): δ = 7.28 (d, *J* = 8.4 Hz, 2 H; Ar*H*), 7.16 (dd, *J* = 7.9, 1.4 Hz, 2 H; Ar*H*), 7.07 (d, *J* = 1.4 Hz, 2 H; Ar*H*), 7.04 (d, *J* = 8.5 Hz, 2 H; Ar*H*), 6.31 (d, *J* = 8.0 Hz, 2 H; Ar*H*), 4.78 (s, 2 H; H–C(7’’)), 2.54 (t, *J* = 7.5 Hz, 2 H; H–C(2’’’)), 1.78 – 1.68 (m, 2 H; H–C(3’’’)), 1.48 – 1.38 (m, 2 H; H–C(4’’’)), 1.31 (s, 24 H; H–C(2’’’’), H–C(3’’’’), H–C(5’’’’), H–C(6’’’’), H–C(2’’’’’), H–C(3’’’’’), H–C(5’’’’’), H–C(6’’’’’)), 1.01 – 0.94 ppm (m, 3 H; H–C(5’’’)); ^{13}C NMR (126 MHz, C*D*Cl$_3$): δ = 179.34, 172.42, 150.06, 144.86, 136.00, 133.19, 131.16, 127.16, 122.26, 121.17, 111.83, 83.76, 48.57, 33.76, 26.89, 24.95, 22.32, 13.82 ppm; m.p. 183 – 186 °C; IR (ATR): υ = 1706 cm^{-1} (*s*, C=O); HR-ESI-MS: m/z (%): 623.3457 ([*M*]$^-$, calcd for $C_{36}H_{45}O_7N^{10}B_2^-$: 623.3455).

4-((10*H*-phenoxazin-10-yl)methyl)benzonitrile

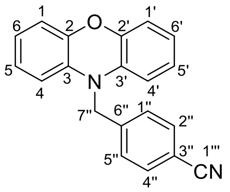

Phenoxazine (250 mg, 1.37 mmol) was dissolved in THF (15 mL). The mixture was cooled to 0 °C and left to stir for 10 min. NaH – 60% mineral oil (68 mg, 1.78 mmol) was slowly added. After 10 min, 4-(bromomethyl)benzonitrile (269 mg, 1.37 mmol) was slowly added. The solution was left to stir at RT for 7 h. The mixture was quenched with water (10 mL). After separation of the phases, the aqueous layer was extracted with EtOAc (2 x 20 mL). The combined organic layers were washed with water (3 x 20 mL), and brine (20 mL), dried over $MgSO_4$, filtered and evaporated *in vacuo*. FC (SiO_2; petroleum ether/EtOAc 90:10) gave 4-((10*H*-phenoxazin-10-yl)methyl)benzonitrile (313 mg, 77 %) as a dark brown solid.

^{1}H NMR (300 MHz, C*D*Cl$_3$): δ = 7.64 (d, *J* = 8.3 Hz, 2 H; Ar*H*), 7.44 (d, *J* = 8.0 Hz, 2 H; Ar*H*), 6.78 – 6.62 (m, 6 H; Ar*H*), 6.28 – 6.19 (m, 2 H; Ar*H*), 4.82 ppm (s, 2 H; H–C(7'')); ^{13}C NMR (75.5 MHz, C*D*Cl$_3$): δ = 145.28, 142.97, 142.51, 132.96, 132.74, 129.85, 127.07, 123.92, 121.94, 115.78, 112.05, 49.35 ppm; m.p. 143 – 145 °C; IR (ATR): υ = 2229 (*m*, C≡N), 1112 cm^{-1} (*s*, C–O); FTMS + p NSI MS: m/z (%): 299.1182 ([*M* + H]$^+$, calcd for $C_{20}H_{15}N_2O^+$: 299.1179).

4-((3,7-dibromo-10*H*-phenoxazin-10-yl)methyl)benzonitrile

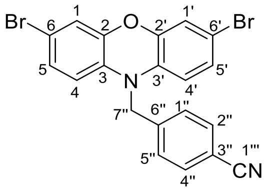

4-((10*H*-phenoxazin-10-yl)methyl)benzonitrile (200 mg, 0.67 mmol) was dissolved in chloroform (20 mL). NBS (238 mg, 1.34 mmol) was slowly added to the mixture, which was left to stir at RT for 2 h. The mixture was quenched with water. After separation of phases, the organic layer was washed with water (3 x 20 mL) and brine (20 mL), dried over $MgSO_4$, filtered and evaporated *in vacuo*. This gave 4-((3,7-dibromo-10*H*-phenoxazin-10-yl)methyl)benzonitrile (271 mg, 89 %) as a green oil.

^{1}H NMR (300 MHz, C*D*Cl$_3$): δ = 7.31 (d, *J* = 8.4 Hz, 2 H; Ar*H*), 7.03 (d, *J* = 8.6 Hz, 2 H; Ar*H*), 6.76 – 6.57 (m, 4 H; Ar*H*), 6.32 (d, *J* = 8.3 Hz, 2 H; Ar*H*), 4.77 ppm (s, 2 H; H–C(7'')); ^{13}C NMR (75.5 MHz, C*D*Cl$_3$): δ = 130.15, 129.64, 127.41, 126.86, 126.71, 126.58, 126.43, 125.37, 118.80, 114.60, 113.45, 49.02 ppm; IR (ATR): υ = 2228 cm^{-1} (*w*, C≡N); TOF MS ASAP: m/z (%): 456.9373 ([*M* + H]$^+$, calcd for $C_{20}H_{12}{}^{81}Br_2N_2O^+$: 456.9328).

4-((3,7-bis(4,4,5,5-tetramethyl-1,3,2-dioxaborolan-2-yl)-10*H*-phenoxazin-10-yl)methyl)benzonitrile

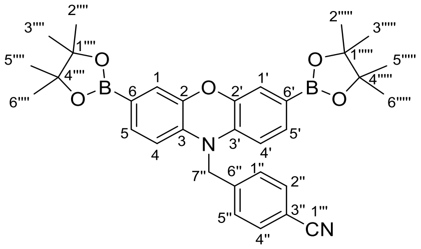

4-((3,7-dibromo-10*H*-phenoxazin-10-yl)methyl)benzonitrile (150 mg, 0.33 mmol), bis(pinacolato)diboron (252 mg, 0.99 mmol) and KOAc (194 mg, 1.98 mmol) were dissolved in DMF (25 mL), degased under argon and treated with [PdCl$_2$(dppf)] (24 mg, 0.033 mmol). The mixture was refluxed at 90 °C for 6 h, and cooled to RT after completion. After separation of the phases, the aqueous layer was washed with EtOAc (3 x 25 mL). The combined organic layers were washed with water (3 x 25 mL), dried over MgSO$_4$, filtered and evaporated *in vacuo*. FC (SiO$_2$; petroleum ether/EtOAc 80:20) gave 4-((3,7-bis(4,4,5,5-tetramethyl-1,3,2-dioxaborolan-2-yl)-10*H*-phenoxazin-10-yl)methyl)benzonitrile (90 mg, 50 %) as a dark red oil.

^{1}H NMR (300 MHz, (C*D*$_3$)$_2$CO): δ = 7.78 (d, *J* = 8.1 Hz, 2 H; Ar*H*), 7.57 (d, *J* = 8.1 Hz, 2 H; Ar*H*), 7.14 (dd, *J* = 7.9, 1.4 Hz, 2 H; Ar*H*), 7.00 (d, *J* = 1.3 Hz, 2 H; Ar*H*), 6.51 (d, *J* = 7.9 Hz, 2 H; Ar*H*), 5.08 (s, 2 H; H–C(7’’)), 1.30 ppm (s, 24 H; H–C(2’’’’), H–C(3’’’’), H–C(5’’’’), H–C(6’’’’), H–C(2’’’’’), H–C(3’’’’’), H–C(5’’’’’), H–C(6’’’’’)); ^{13}C NMR (126 MHz, (C*D*$_3$)$_2$CO): δ = 145.21, 143.14, 136.44, 133.90, 133.47, 132.04, 128.29, 121.59, 112.89, 84.39, 48.29, 25.15 ppm; IR (ATR): υ = 2229 cm^{-1} (*w*, C≡N); FTMS + p APCI corona MS: m/z (%): 548.2866 ([*M*]$^+$, calcd for $C_{32}H_{36}B_2N_2O_5^+$: 548.2877).

10-(4-(prop-2-yn-1-yloxy)benzyl)-3,7-bis(4,4,5,5-tetramethyl-1,3,2-dioxaborolan-2-yl)-10*H*-phenoxazine

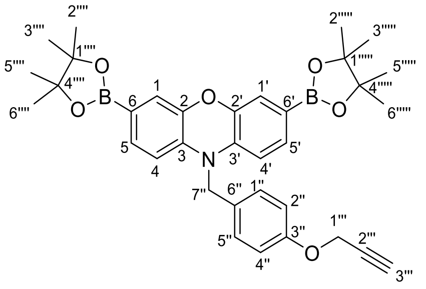

4-((3,7-bis(4,4,5,5-tetramethyl-1,3,2-dioxaborolan-2-yl)-10*H*-phenoxazin-10-yl)methyl)phenol (200 mg, 0.37 mmol) was dissolved in THF (30 mL). NaH – 60 % mineral oil (22 mg, 0.56 mmol) was added, followed by dropwise addition of propargyl bromide – 80 wt% in toluene (0.09 mL, 0.56 mmol). The mixture was stirred at RT for 20 h. The reaction was quenched with MeOH (20 mL), diluted with EtOAc (30 mL), washed with water (3 x 60 mL), dried over $MgSO_4$, filtered and evaporated *in vacuo*. FC (SiO_2; petroleum ether/EtOAc 80:20) gave 3,7-dibromo-10-(4-(prop-2-yn-1-yloxy)benzyl)-10*H*-phenoxazine (110 mg, 51 %) as a dark yellow oil.

^{1}H NMR (300 MHz, $CDCl_3$): δ = 7.20 (d, *J* = 8.4 Hz, 2 H; Ar*H*), 7.15 (dd, *J* = 7.9, 1.4 Hz, 2 H; Ar*H*), 7.06 (d, *J* = 1.4 Hz, 2 H; Ar*H*), 6.93 (d, *J* = 8.7 Hz, 2 H; Ar*H*), 6.33 (d, *J* = 8.0 Hz, 2 H; Ar*H*), 4.74 (s, 2 H; H–C(7’’)), 4.66 (d, *J* = 2.4 Hz, 2 H; H–C(1’’’)), 2.51 (t, *J* = 2.3 Hz, 1 H; H–C(3’’’)), 1.26 ppm (s, 24 H; H–C(2’’’’), H–C(3’’’’), H–C(5’’’’), H–C(6’’’’), H–C(2’’’’’), H–C(3’’’’’), H–C(5’’’’’), H–C(6’’’’’)); ^{13}C NMR (126 MHz, $(CD_3)_2CO$): δ = 158.03, 145.41, 137.01, 132.17, 129.51, 128.42, 128.38, 121.56, 121.51, 116.26, 116.23, 113.19, 84.49, 79.92, 48.07, 25.32 ppm; IR (ATR): υ = 2160 (*w*, C≡C), 1346 cm^{-1} (*s*, C–O); FTMS + p APCI corona MS: m/z (%): 579.2958 ([*M*]$^+$, calcd for $C_{34}H_{39}{}^{11}B_2NO_6^+$: 579.2970).

pent-4-ynoyl chloride

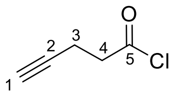

4-Pentynoic acid (300 mg, 3.06 mmol) was dissolved in DCM/DMF (10 ml/10 µL). Thionyl chloride (1.16 mL, 15.9 mmol) was added slowly to the mixture. The solution was refluxed at 40 °C for 6 h. Solvent was evaporated *in vacuo* giving pent-4-ynoyl chloride (270 mg, 76 %) as a light brown oil.

^{1}H NMR (500 MHz, $CDCl_3$): δ = 3.13 (t, *J* = 7.1 Hz, 2 H; H–C(4)), 2.57 (td, *J* = 7.1, 2.7 Hz, 2 H; H–C(3)), 2.04 ppm (t, *J* = 2.7 Hz, 1 H; H–C(1)); ^{13}C NMR (126 MHz, $CDCl_3$): δ = 172.12, 80.38, 70.26, 45.63, 14.69 ppm; IR (ATR): υ = 2119 (*w*, C≡C), 1693 cm^{-1} (*s*, C=O); HR-ESI-MS: m/z (%): 116.0097 ([*M*]$^+$, calcd for $C_5H_5ClO^+$: 116.0029).

4-((3,7-bis(4,4,5,5-tetramethyl-1,3,2-dioxaborolan-2-yl)-10*H*-phenoxazin-10-yl)methyl)phenyl pent-4-ynoate

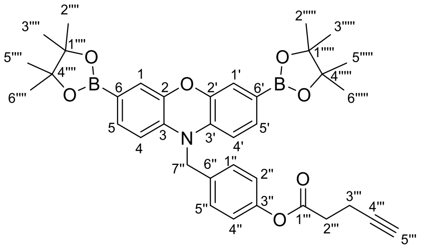

4-((3,7-bis(4,4,5,5-tetramethyl-1,3,2-dioxaborolan-2-yl)-10*H*-phenoxazin-10-yl)methyl)phenol (200 mg, 0.37 mmol) was dissolved in DCM (40 mL). The solution was cooled to 0 °C, followed by the addition of triethylamine (0.1 mL, 0.74 mmol), and pent-4-ynoyl chloride (86 mg, 0.74 mmol). The mixture was allowed to warm up to RT and stirred for 3 h. The reaction was quenched with water. After separation of the phases, the organic layer was washed with water (3 x 30 mL), dried over $MgSO_4$, filtered and evaporated *in vacuo*. FC (SiO_2; petroleum ether/EtOAc 80:20) gave 4-((3,7-bis(4,4,5,5-tetramethyl-1,3,2-dioxaborolan-2-yl)-10*H*-phenoxazin-10-yl)methyl)phenyl pent-4-ynoate (107 mg, 47 %) as a dark orange oil.

^{1}H NMR (300 MHz, $CDCl_3$): δ = 7.33 – 7.23 (m, 2 H; Ar*H*), 7.20 – 7.10 (m, 2 H; Ar*H*), 7.10 – 7.01 (m, 4 H; Ar*H*), 6.30 (d, *J* = 7.9 Hz, 2 H; Ar*H*), 4.78 (s, 2 H; H–C(7’’)), 2.83 – 2.67 (m, 1 H; H–C(5’’’)), 2.63 – 2.48 (m, 2 H; H–C(2’’’)), 2.07 – 1.97 (m, 2 H; H–C(3’’’)), 1.26 ppm (s, 24 H; H–C(2’’’’), H–C(3’’’’), H–C(5’’’’), H–C(6’’’’), H–C(2’’’’’), H–C(3’’’’’), H–C(5’’’’’), H–C(6’’’’’)); ^{13}C NMR (126 MHz, $CDCl_3$): δ = 170.40, 149.86, 144.84, 135.97, 134.87, 133.47, 131.37, 127.83, 127.21, 122.18, 121.19, 111.80, 83.77, 83.31, 75.26, 69.59, 33.62, 24.67 ppm; IR (ATR): υ = 2161 (*w*, C≡C), 1280 (*m*, C–N) 1121 cm^{-1} (*s*, C–O); FTMS + p APCI corona MS: m/z (%): 622.3123 ([*M* + H]$^+$, calcd for $C_{36}H_{42}{}^{11}B_2NO_7^+$: 622.3154).

4-((3,7-bis(4,4,5,5-tetramethyl-1,3,2-dioxaborolan-2-yl)-10*H*-phenoxazin-10-yl)methyl)phenyl 4-(4-(bis(2-chloroethyl)amino) phenyl) butanoate

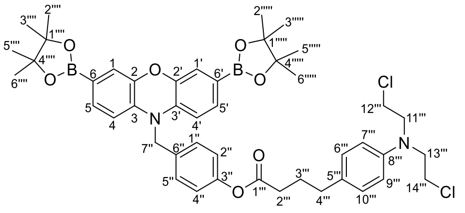

Chlorambucil (37 mg, 0.12 mmol) and HATU (49 mg, 0.13 mmol) were dissolved in DMF (5 mL) to which 4-((3,7-bis(4,4,5,5-tetramethyl-1,3,2-dioxaborolan-2-yl)-10*H*-phenoxazin-10-yl)methyl)phenol (100 mg, 0.18 mmol) and DIPEA (0.06 ml, 0.36 mmol) in DMF (5 mL) was added. The solution was left to stir at RT for 24 h. After completion, the mixture was diluted with EtOAc (15 mL), washed with water (1 x 20 mL) and brine (3 x 20 mL), dried with $MgSO_4$, filtered and evaporated *in vacuo*. FC (SiO_2; petroleum ether/EtOAc 80:20) gave 4-((3,7-bis(4,4,5,5-tetramethyl-1,3,2-dioxaborolan-2-yl)-10-phenoxazin-10-yl)methyl)phenyl 4-(4-(bis(2-chloroethyl) amino) phenyl)butanoate (30 mg, 20 %) as an orange solid.

^{1}H NMR (500 MHz, C*D*Cl$_3$): δ = 7.48 – 7.43 (m, 4 H; Ar*H*), 7.34 (dd, *J* = 7.9, 1.3 Hz, 2 H; Ar*H*), 7.29 (d, *J* = 8.2 Hz, 2 H; Ar*H*), 7.20 (d, *J* = 8.2 Hz, 2 H; Ar*H*), 6.82 (d, *J* = 8.3 Hz, 2 H; Ar*H*), 6.50 (d, *J* = 7.9 Hz, 2 H; Ar*H*), 4.96 (s, 2 H; H–C(7′′)), 3.89 (t, *J* = 7.0 Hz, 4 H; H–C(12′′′),H–C(14′′′)), 3.80 (t, *J* = 7.0 Hz, 4 H; H–C(11′′′), H–C(13′′′)), 2.83 (t, *J* = 7.5 Hz, 2 H; H–C(2′′′)), 2.74 (t, *J* = 7.3 Hz, 2 H; H–C(4′′′)), 2.21 (p, *J* = 7.4 Hz, 2 H; H–C(3′′′)), 1.49 ppm (s, 24 H; H–C(2′′′′), H–C(3′′′′), H–C(5′′′′), H–C(6′′′′), H–C(2′′′′′), H–C(3′′′′′), H–C(5′′′′′), H–C(6′′′′′)); ^{13}C NMR (126 MHz, C*D*Cl$_3$): δ = 172.00, 149.84, 144.70, 144.43, 135.83, 133.09, 131.01, 130.33, 129.76, 127.03, 122.08, 121.03, 112.23, 111.67, 83.60, 53.61, 48.36, 40.52, 33.93, 33.64, 26.68, 24.81 ppm; m.p.: 105 – 108 °C; IR (ATR): υ = 1755 (*m*, C=O), 1113 cm^{-1} (*s*, C–O–C); HR-ESI-MS: m/z (%): 824.3514 ([*M*]$^+$, calcd for $C_{45}H_{54}O_7N_2{}^{35}Cl_2{}^{10}B_2{}^+$: 824.3567).

4-((3,7-bis(4,4,5,5-tetramethyl-1,3,2-dioxaborolan-2-yl)-10*H*-phenoxazin-10-yl)methyl)phenyl 2-(1-(4-chlorobenzoyl)-5-me-thoxy-2-methyl-1*H*-indol-3-yl)acetate

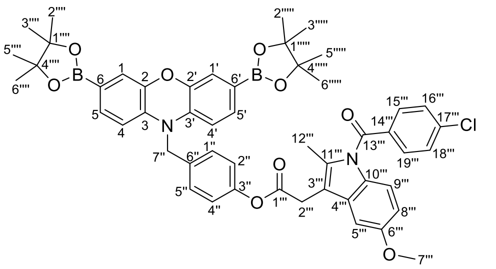

Indomethacin (43 mg, 0.12 mmol) and HATU (49 mg, 0.13 mmol) were dissolved in DMF (5 mL) to which 4-((3,7-bis(4,4,5,5-tetramethyl-1,3,2-dioxaborolan-2-yl)-10*H*-phenoxazin-10-yl)methyl)phenol (100 mg, 0.18 mmol) and DIPEA (0.06 ml, 0.36 mmol) in DMF (5 mL) was added. The solution was left to stir at RT for 24 h. After completion, the mixture was diluted with EtOAc (15 mL), washed with water (1 x 20 mL) and brine (3 x 20 mL), dried with $MgSO_4$, filtered and evaporated *in vacuo*. FC (SiO_2; petroleum ether/EtOAc 80:20) gave 4-((3,7-bis(4,4,5,5-tetramethyl-1,3,2-dioxaborolan-2-yl)-10*H*-phenoxazin-10-yl)methyl)phenyl 2-(1-(4-chlorobenzoyl)-5-methoxy-2-methyl-1*H*-indol-3-yl)acetate (45 mg, 28 %) as a dark orange solid.

^{1}H NMR (500 MHz, C*D*Cl$_3$): δ = 7.67 (dd, *J* = 8.4, 1.4 Hz, 2 H; Ar*H*), 7.49 – 7.45 (m, 2 H; Ar*H*), 7.16 – 7.10 (m, 4 H; Ar*H*), 7.08 – 7.03 (m, 4 H; Ar*H*), 6.89 (d, *J* = 9.1 Hz, 1 H; Ar*H*), 6.79 – 6.76 (m, 1 H; Ar*H*), 6.71 – 6.67 (m, 1 H; Ar*H*), 6.29 (d, J = 7.9 Hz, 2 H; Ar*H*), 4.76 (s, 2 H; H–C(7′′)), 3.89 (s, 2 H; H–C(2′′′)), 3.83 (s, 3 H; H–C(7′′′)), 2.44 (s, 3 H; H–C(12′′′)), 1.31 ppm (s, 24 H; H–C(2′′′′), H–C(3′′′′), H–C(5′′′′), H–C(6′′′′), H–C(2′′′′′), H–C(3′′′′′), H–C(5′′′′′), H–C(6′′′′′)); ^{13}C NMR (126 MHz, C*D*Cl$_3$): δ = 169.27, 168.30, 156.13, 149.84, 144.72, 144.69, 139.33, 136.19, 136.05, 135.78, 131.18, 130.99, 129.14, 127.30, 127.07, 121.92, 121.05, 115.81, 115.02, 111.86, 111.62, 101.17, 83.63, 83.62, 55.75, 48.30, 30.56, 24.80, 13.42. ppm; m.p.: 162 – 165 °C; IR (ATR): υ = 1591 (*m*, C=O), 1346 cm^{-1} (*s*, C–O–C); HR-ESI-MS: m/z (%): 878.3460 ([*M*]$^+$, calcd for $C_{50}H_{51}O_9N_2{}^{35}Cl^{10}B_2^+$: 878.3542).

3. Schemes and Figures

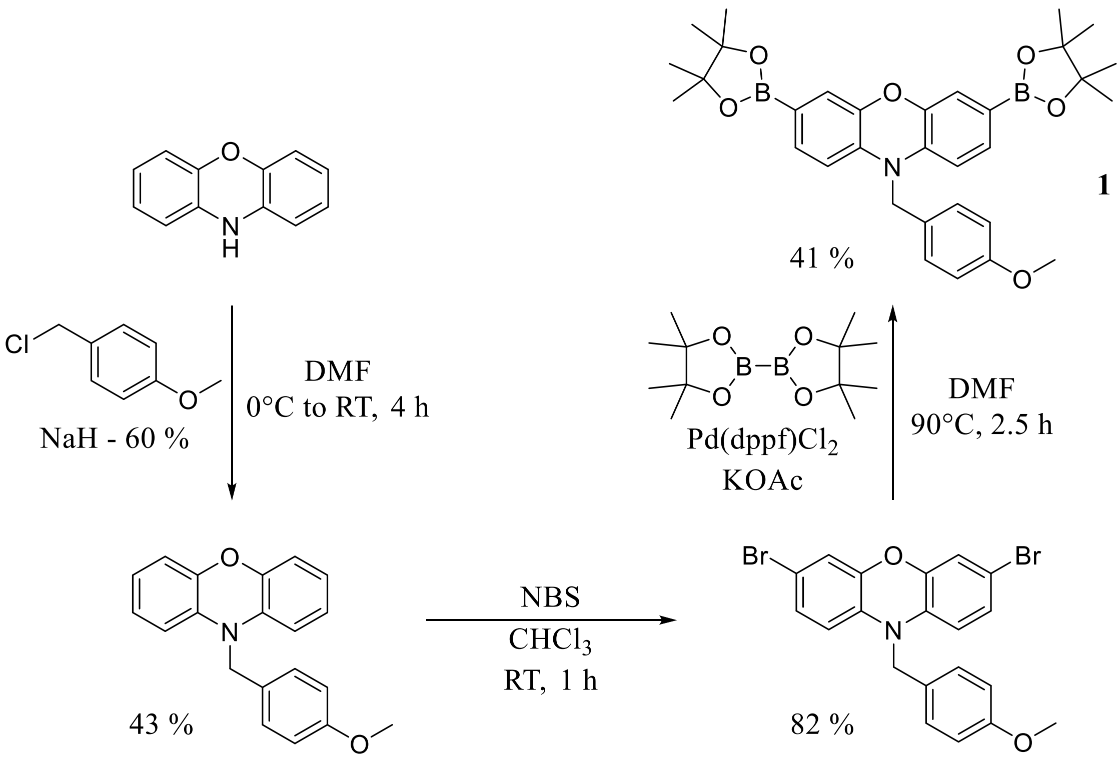

Scheme S1. Synthetic route of **1**.

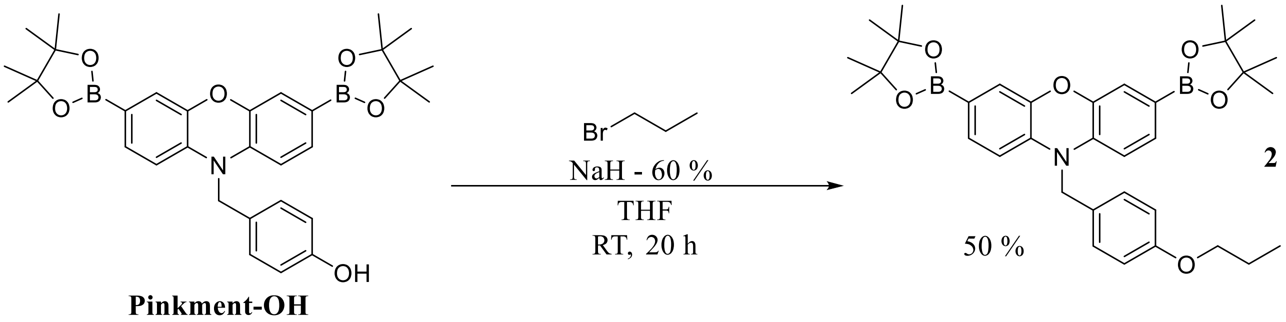

Scheme S2. Synthetic route of **2**.

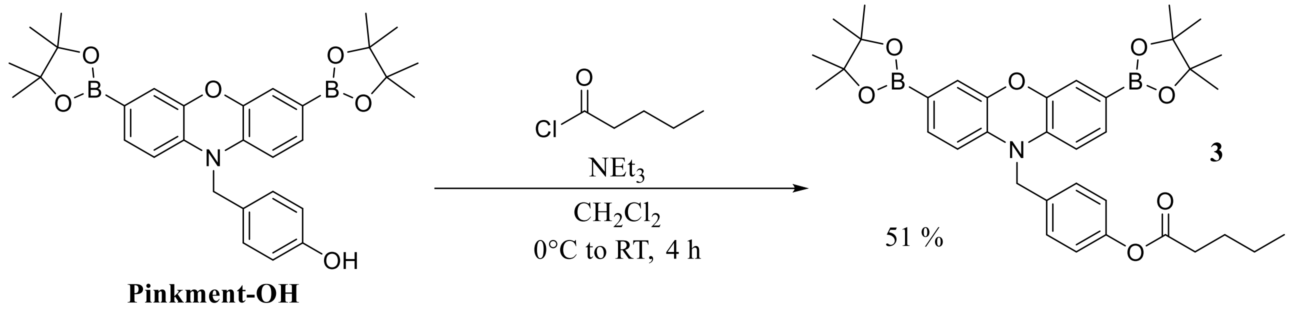

Scheme S3. Synthetic route of **3**.

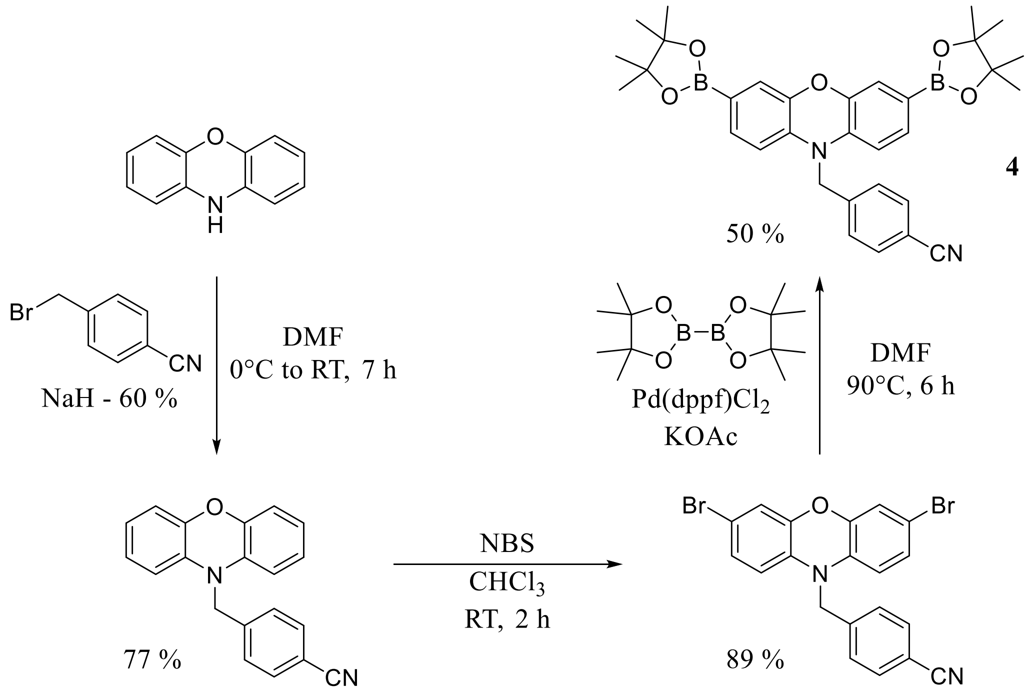

Scheme S4. Synthetic route of **4**.

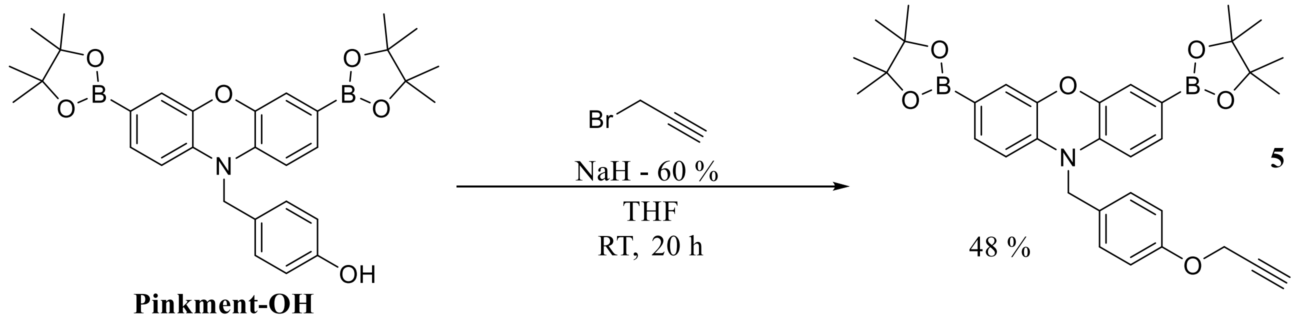

Scheme S5. Synthetic route of **5**.

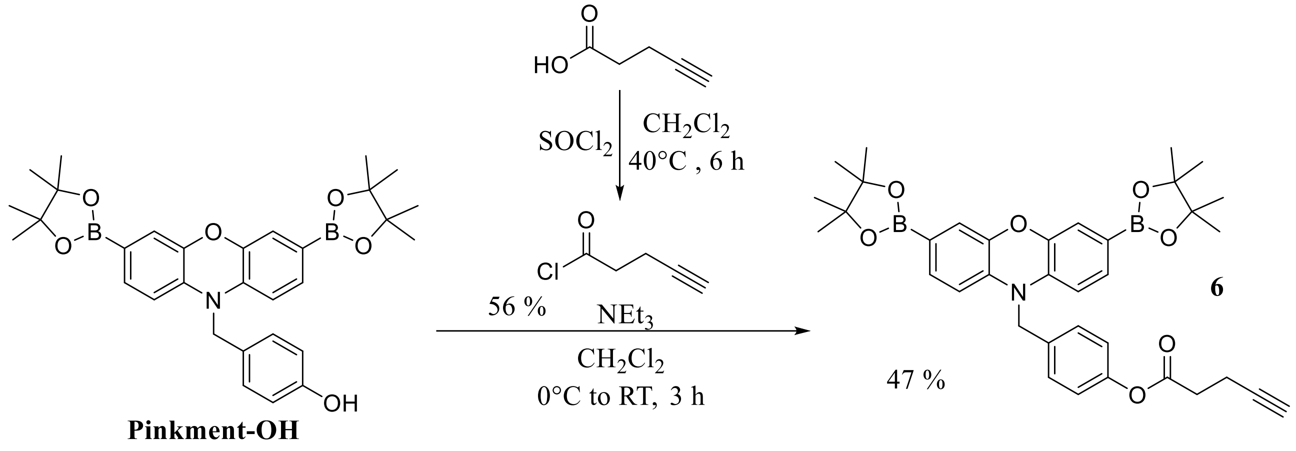

Scheme S6. Synthetic route of **6**.

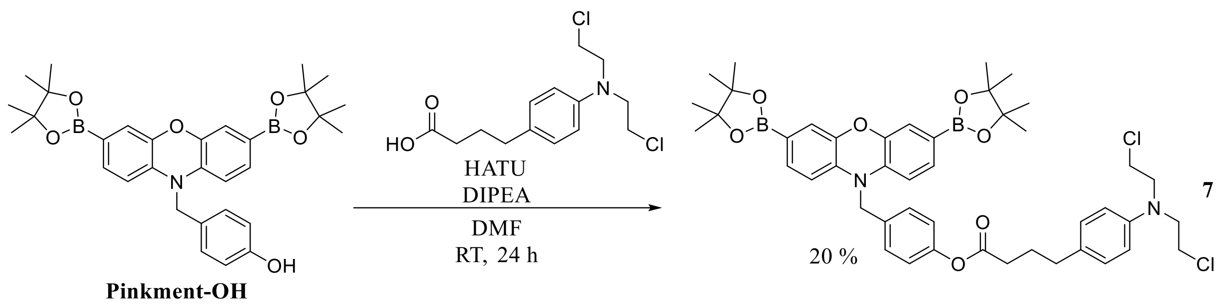

Scheme S7. Synthetic route of theranostic resorufin probe **7**.

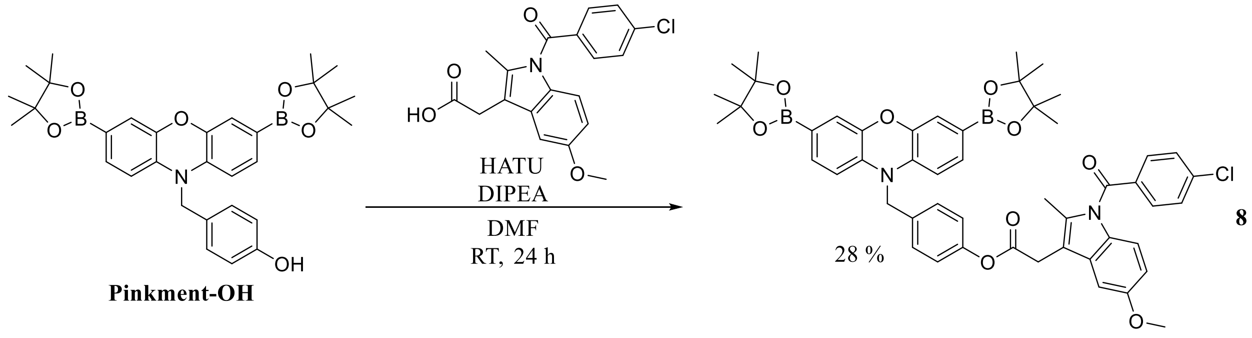

Scheme S8. Synthetic route of theranostic resorufin probe **8**.

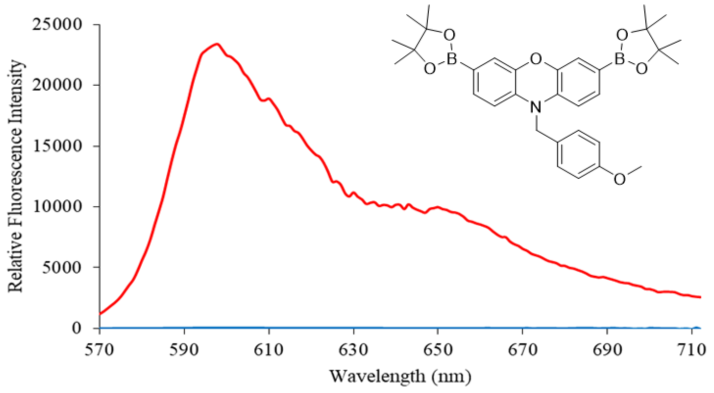

Figure S1. Emission spectra for **1** (500 nM) without (blue) and with $ONOO^-$ (50 µM, red) in 50% DMSO: 50% PBS buffer (52 % MeOH : H_2O, pH = 8.2) at 25 °C. Fluorescence intensities were measured with λ_{ex} = 550 (bandwith: 15) nm on a BMG Labtech CLARIOstar® plate reader.

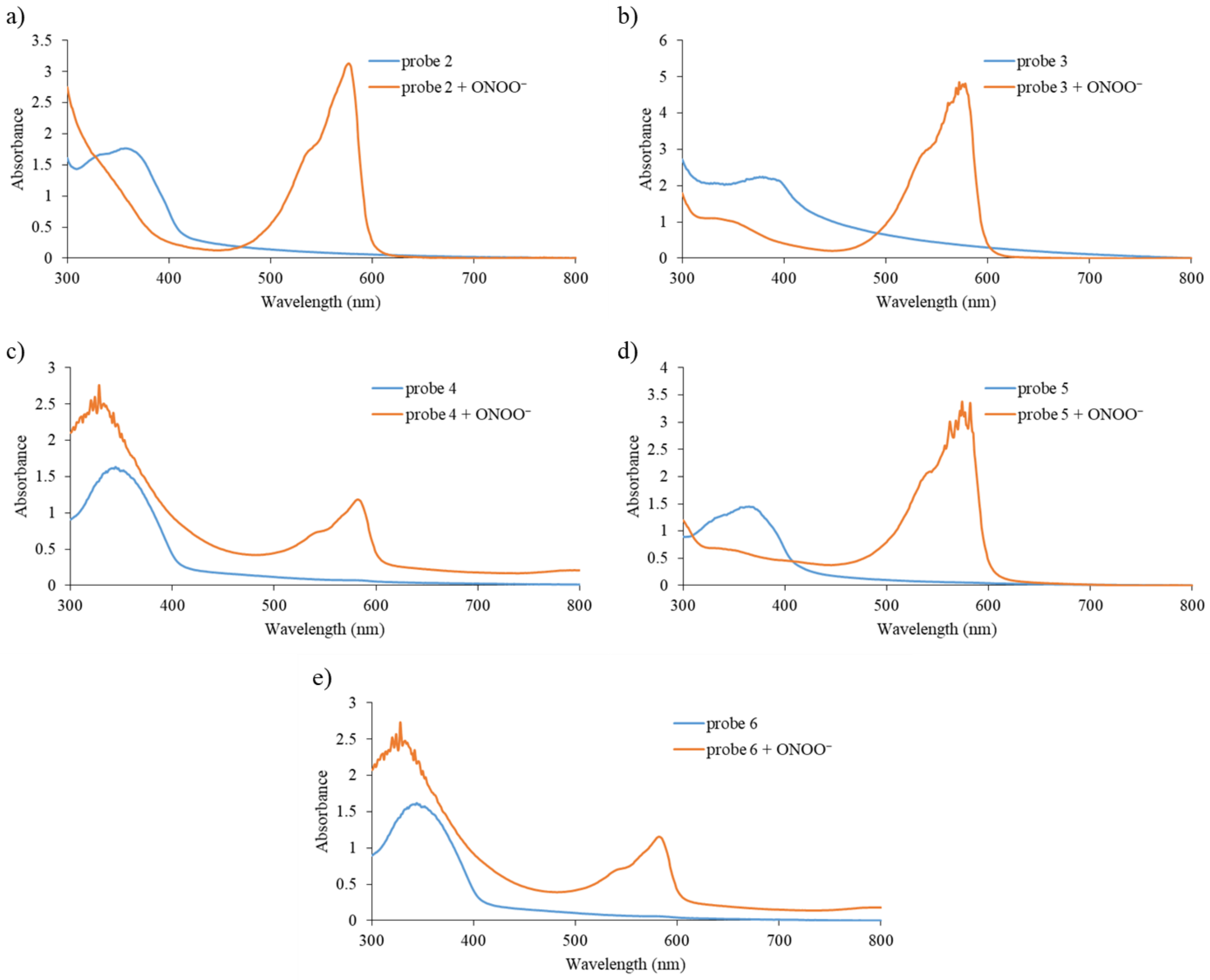

Figure S2. UV spectra of a) probe **2**, b) probe **3**, c) probe **4**, d) probe **5** and e) probe **6** with and without $ONOO^-$ (excess) in PBS buffer 52 % MeOH : H_2O, pH = 8.2 at 25 °C measured on a BMG Labtech CLARIOstar® plate reader.

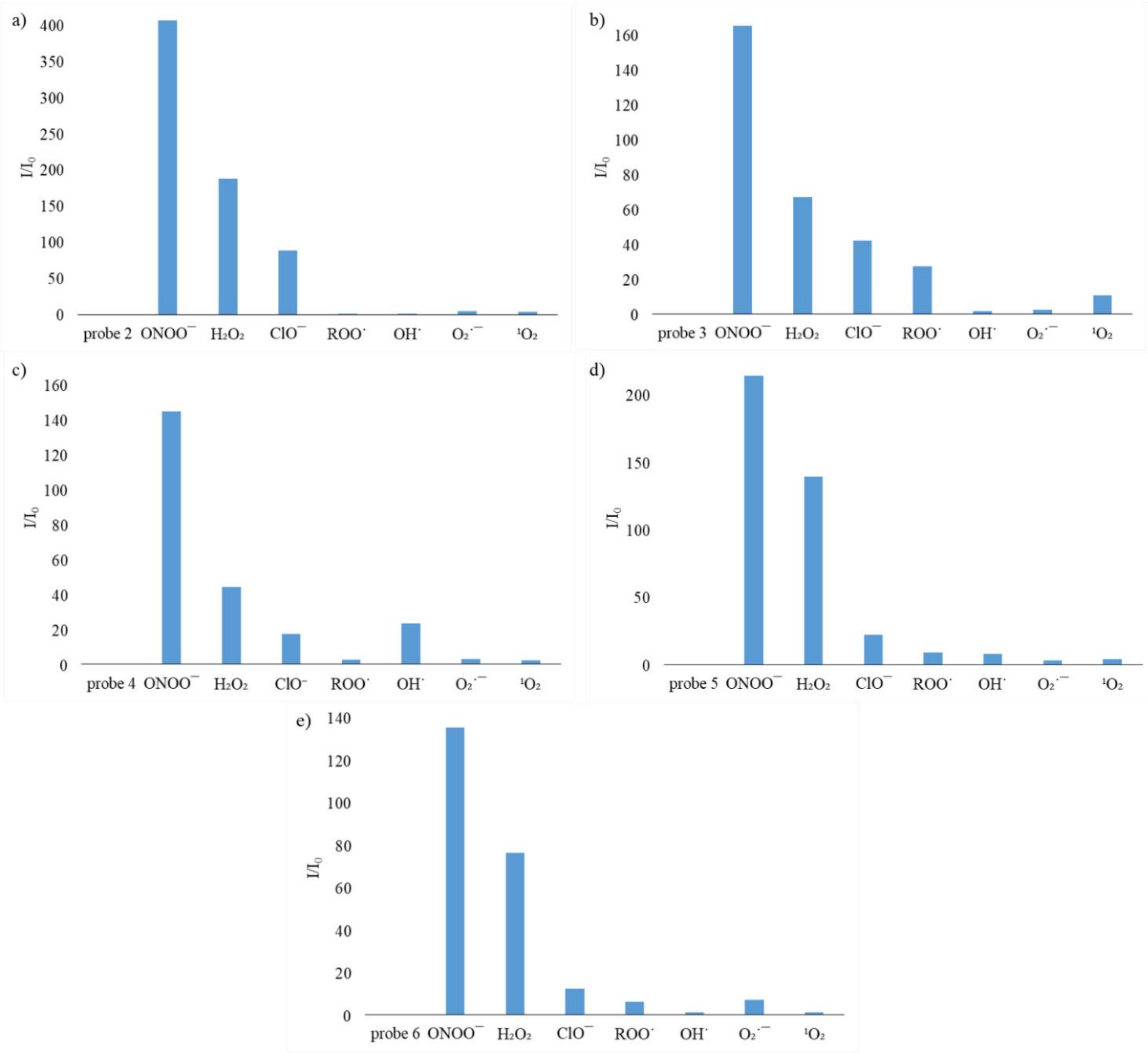

Figure S3. Selectivity data for a) **2** (500 nM), b) **3** (500 nM), c) **4** (500 nM), d) **5** (500 nM) and e) **6** (500 nM) in the presence of $ONOO^-$ (50 µM), OH· (500 µM), $O_2^{\cdot-}$ (500 µM), $^1O_2$ (500 µM) after 5 min. H_2O_2 (1 mM), ROO· (500 µM) and ClO^- (500 µM) were measured after 30 min. The data was obtained in PBS buffer 52 % MeOH : H_2O, pH = 8.2 at 25 °C at λ_{ex} = 550 (bandwith: 15) nm and max λ_{em} = 590 nm on a BMG Labtech CLARIOstar® plate reader.

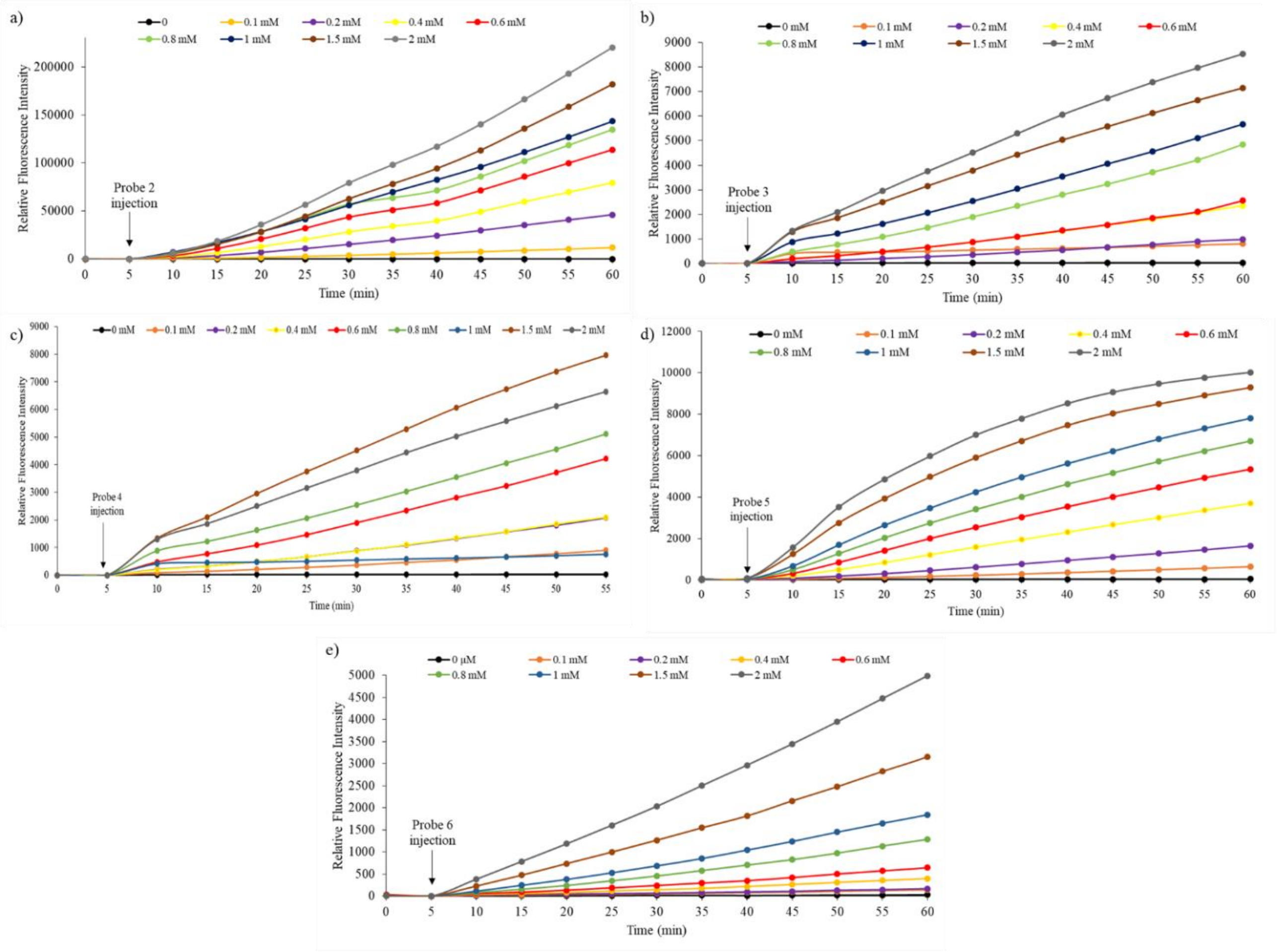

Figure S4. Fluorescence intensity changes over time for probes a) **2** (500 nM), b) **3** (500 nM), c) **4** (500 nM), d) **5** (500 nM) and e) **6** (500 nM) in the presence of H_2O_2 (0.1, 0.2, 0.4, 0.6, 0.8, 1, 1.5, 2 mM) in PBS buffer 52 % MeOH : H_2O, pH = 8.2 at 25 °C. Fluorescence intensities were measured with λ_{ex} = 550 (bandwidth: 15) nm and max λ_{em} = 590 nm on a BMG Labtech CLARIOstar® plate reader.

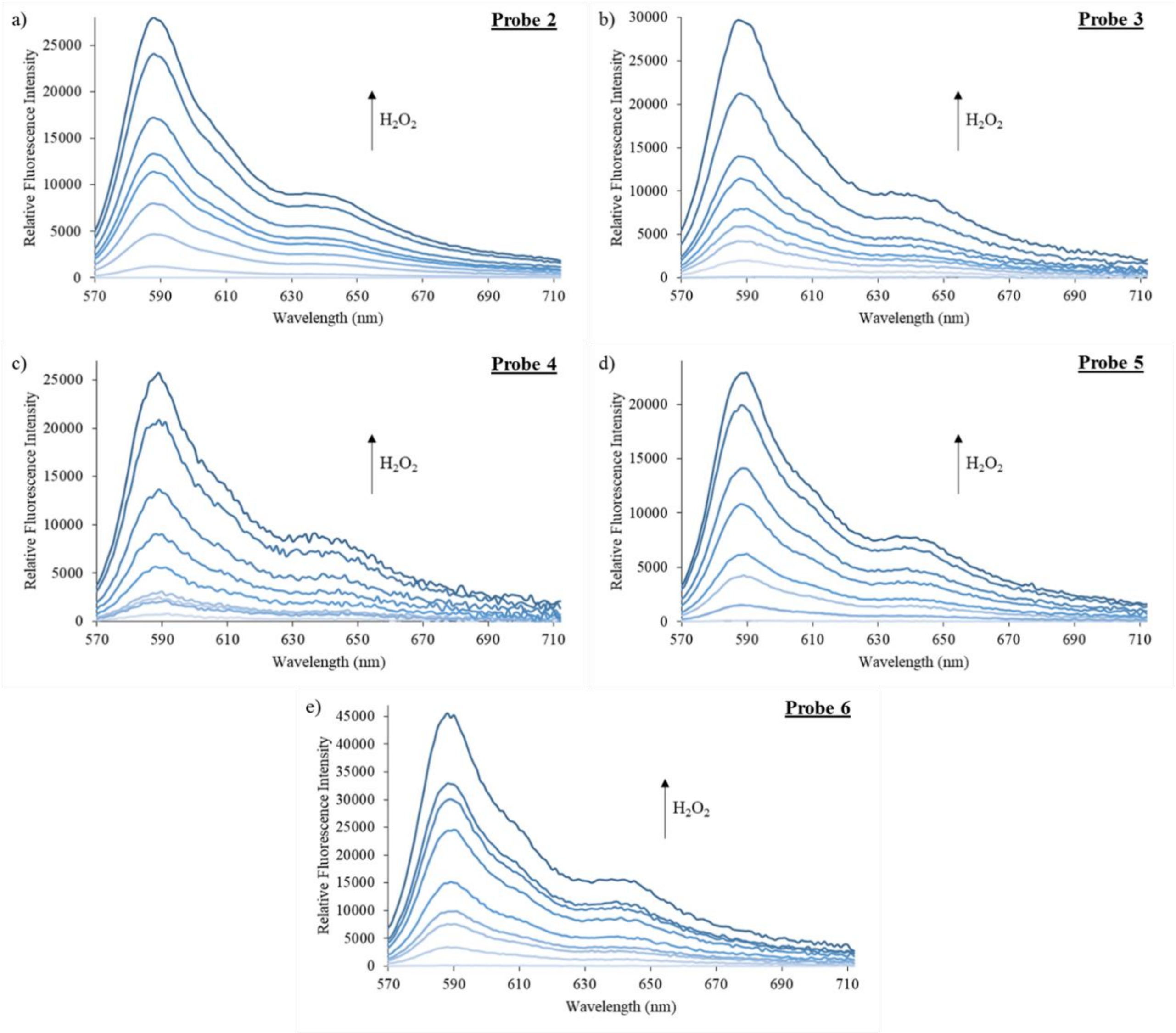

Figure S5. Emission spectra for probes a) **2** (500 nM), b) **3** (500 nM), c) **4** (500 nM), d) **5** (500 nM), and e) **6** (500 nM) in the presence of H_2O_2 (0.1, 0.2, 0.4, 0.6, 0.8, 1, 1.5, 2 mM) after 1 h in PBS buffer 52 % MeOH : H_2O, pH = 8.2 at 25 °C. Fluorescence intensities were measured with λ_{ex} = 550 (bandwidth: 15) nm on a BMG Labtech CLARIOstar® plate reader.

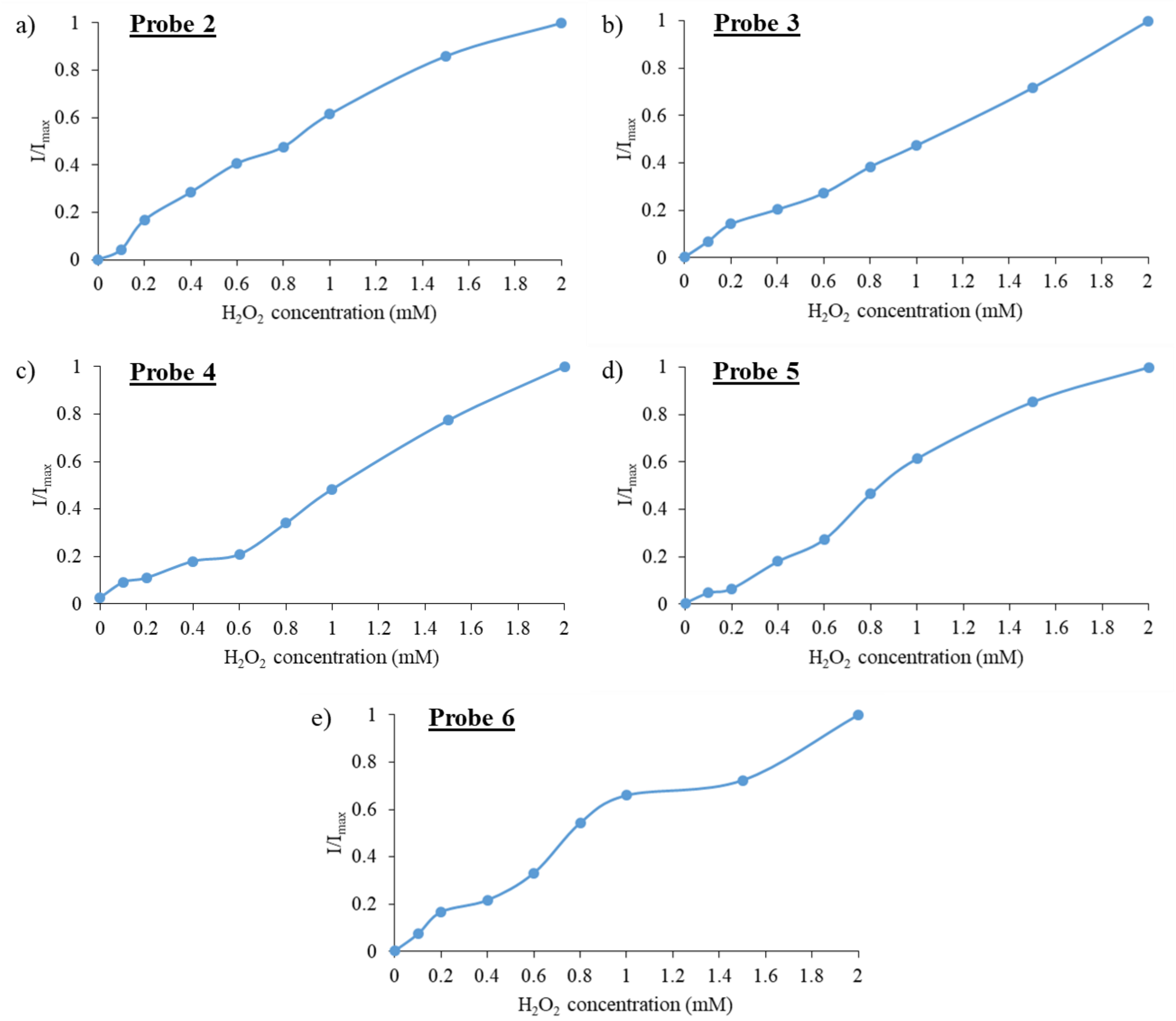

Figure S6. Dose dependence curve (I/I_{max}) for probes a) **2** (500 nM), b) **3** (500 nM), c) **4** (500 nM), d) **5** (500 nM) and e) **6** (500 nM) in the presence of H_2O_2 (0.1, 0.2, 0.4, 0.6, 0.8, 1, 1.5, 2 mM) in PBS buffer 52 % MeOH : H_2O, pH = 8.2 at 25 °C. Fluorescence intensities were measured with λ_{ex} = 550 (bandwidth: 15) nm and max λ_{em} = 590 nm on a BMG Labtech CLARIOstar® plate reader.

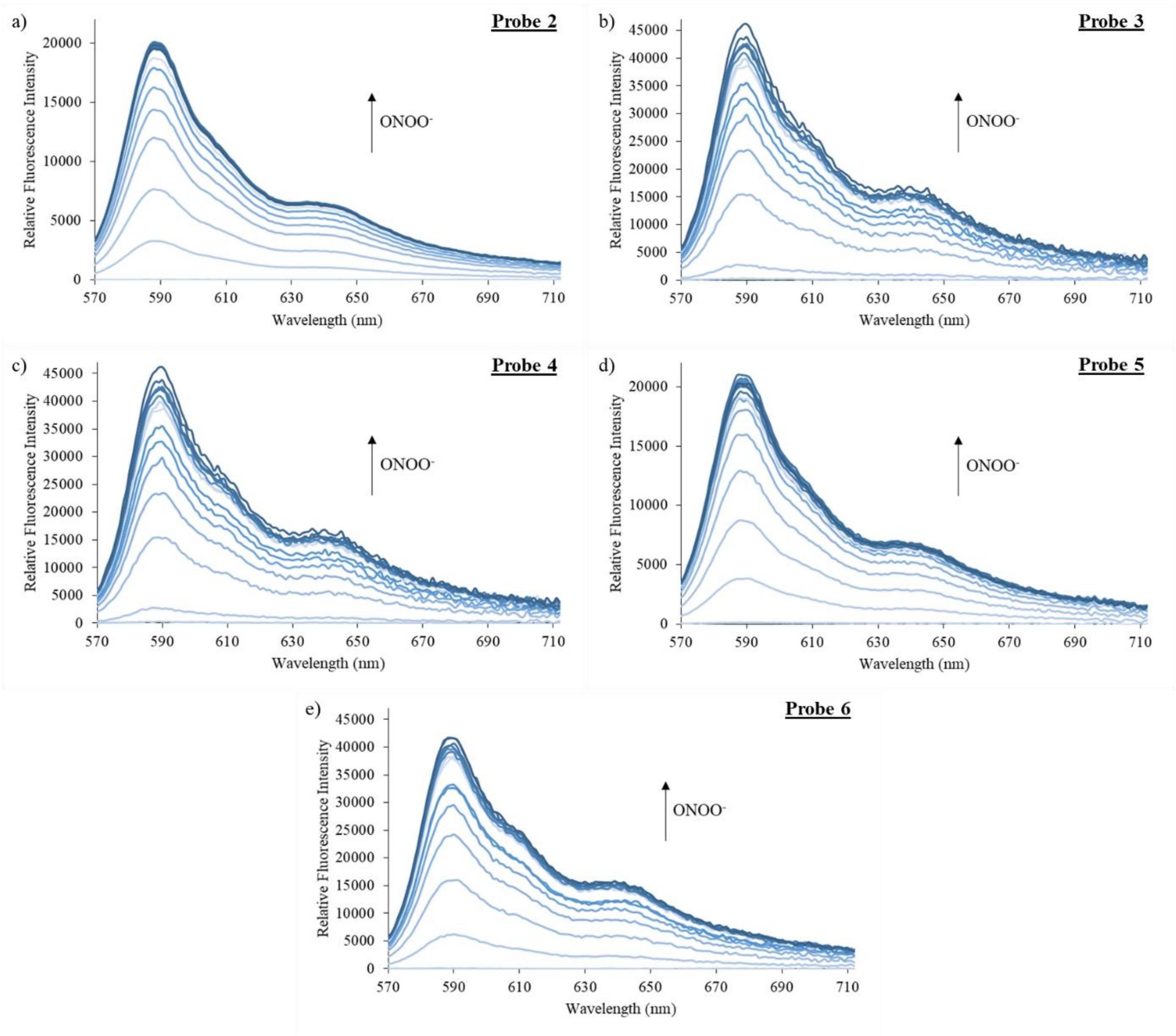

Figure S7. Emission spectra for probes a) **2** (500 nM), b) **3** (500 nM), c) **4** (500 nM), d) **5** (500 nM) and e) **6** (500 nM) in the presence of $ONOO^-$ (1, 2, 3, 4, 5, 6, 7, 8, 9, 10, 20, 30, 40, 50 µM) in PBS buffer 52 % MeOH : H_2O, pH = 8.2 at 25 °C. Fluorescence intensities were measured with λ_{ex} = 550 (bandwidth: 15) nm and max λ_{em} = 590 nm on a BMG Labtech CLARIOstar® plate reader.

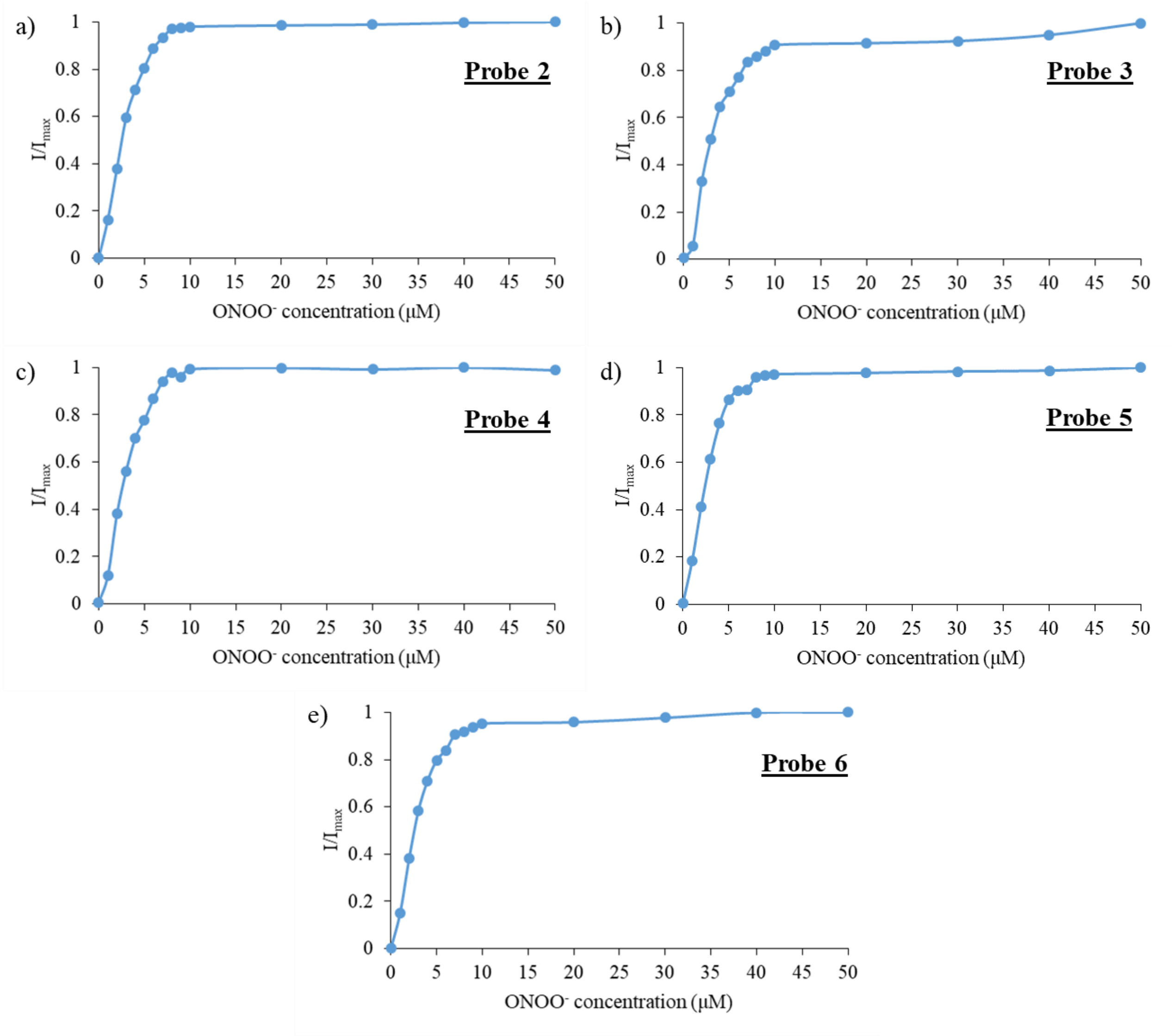

Figure S8. Dose dependence curve (I/I_{max}) for probes a) **2** (500 nM), b) **3** (500 nM), c) **4** (500 nM), d) **5** (500 nM) and e) **6** (500 nM) in the presence of $ONOO^-$ (1, 2, 3, 4, 5, 6, 7, 8, 9, 10, 20, 30, 40,50 µM) in PBS buffer 52 % MeOH : H_2O, pH = 8.2 at 25 °C. Fluorescence intensities were measured with λ_{ex} = 550 (bandwidth: 15) nm and max λ_{em} = 590 nm on a BMG Labtech CLARIOstar® plate reader.

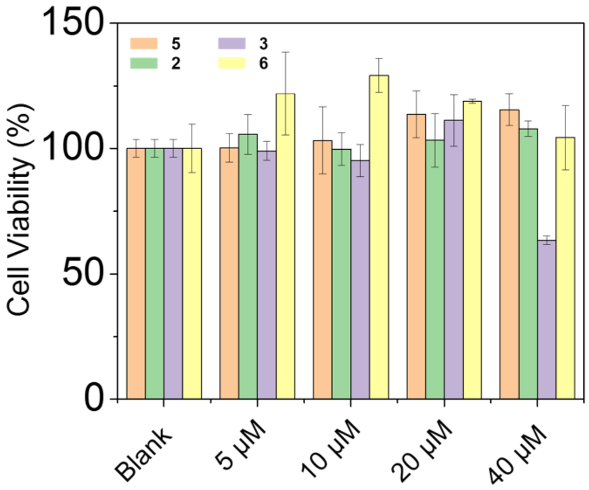

Figure S9. Viability of RAW 264.7 macrophages upon incubation of **2**, **3, 5** and **6** at different probe concentrations (0, 5, 10, 20, and 40 µM) determined by a MTS proliferation assay. Error bars represent s. d. N=3.

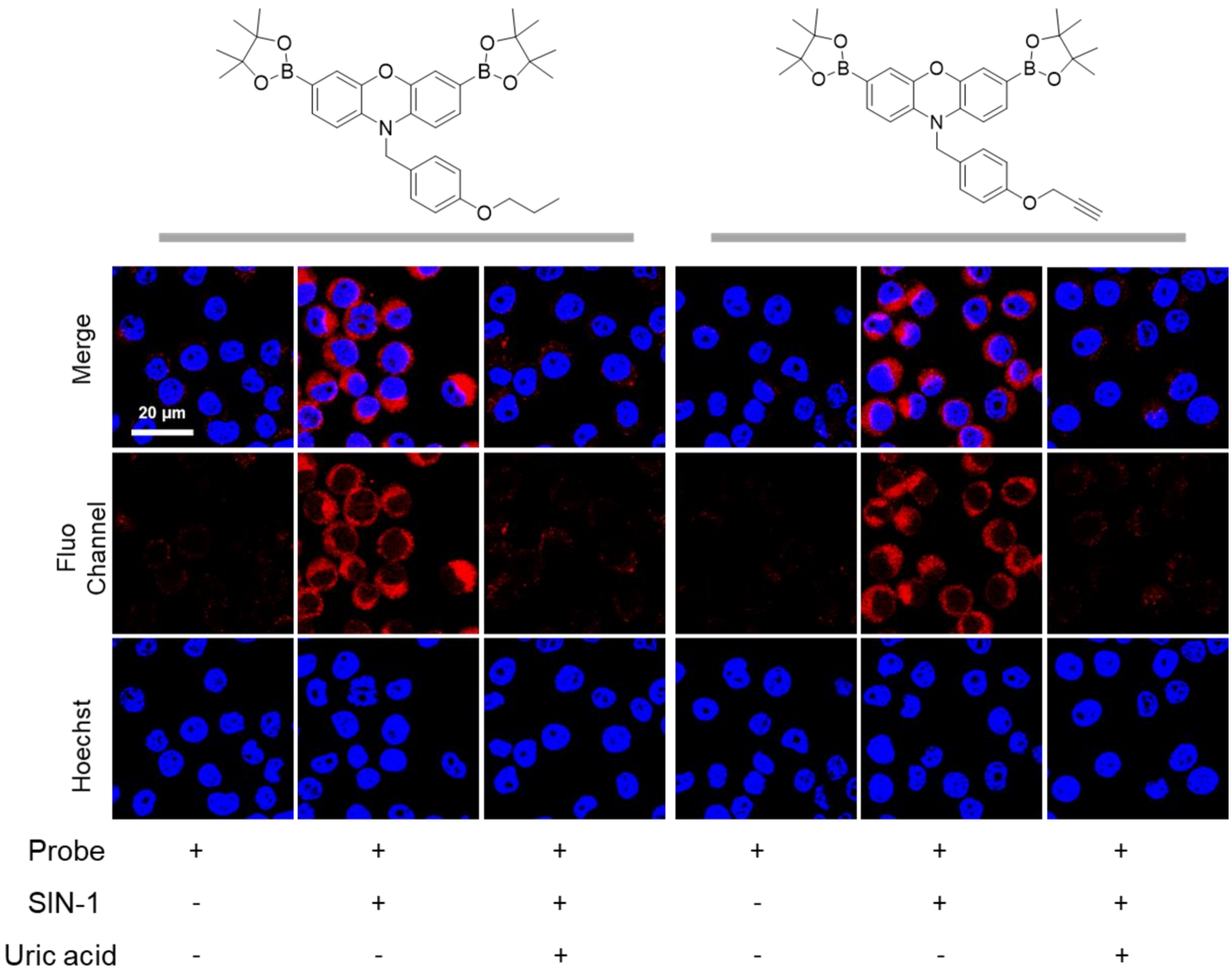

Figure S10. Confocal imaging of RAW 264.7 macrophages were loaded with **2** (20 μM, 30 min), and **5** (20 μM, 30 min) and treated with SIN-1 (500 μM, 30 min) or uric acid (100 μM, 2 h) and SIN-1 (500 μM, 30 min), as indicated. Probe fluorescence was collected at λ_{ex} = 559 nm and λ_{em} = 580-650 nm, respectively. The cell nuclei was stained by Hoechst 33342 and fluorescence collected at λ_{ex} = 405 nm and λ_{em} = 450-480 nm. Scale bar = 20 μm. N = 3.

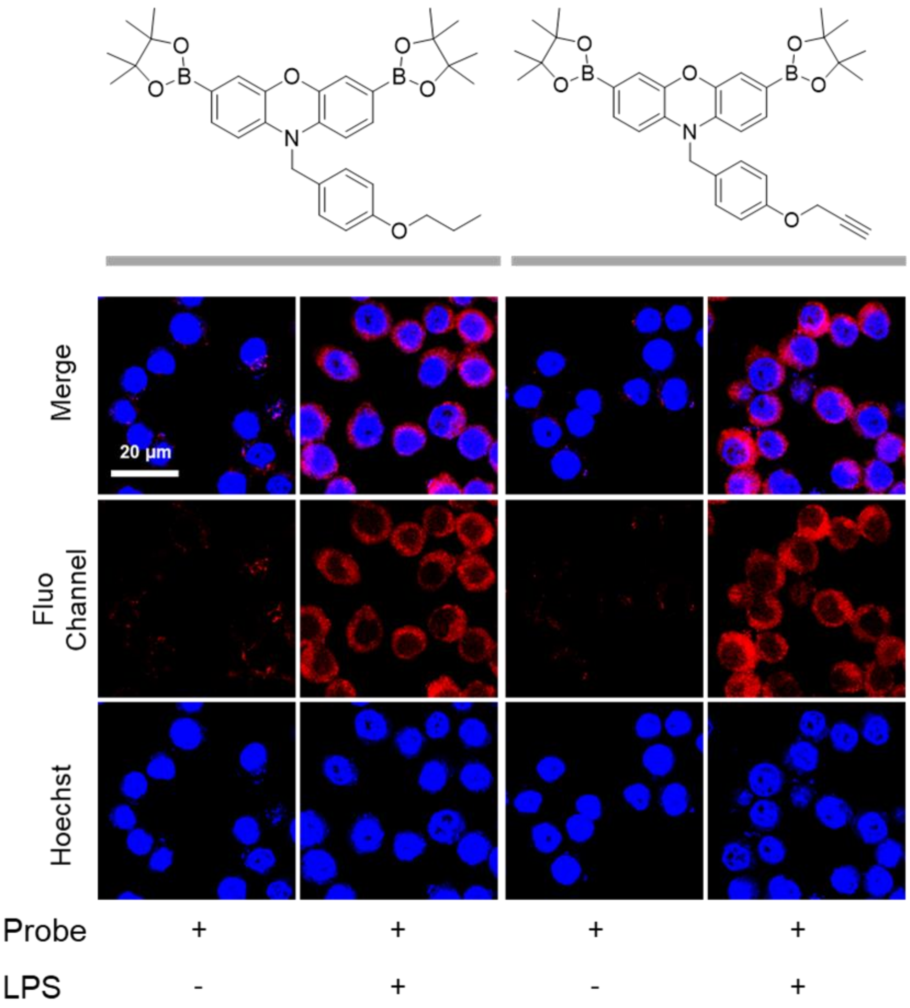

Figure S11. Confocal imaging of RAW 264.7 macrophages primed with LPS (1 µg/ml, 24 h) and then loaded with **2** (20 µM, 30 min), and **5** (20 µM, 30 min) as indicated. Probe fluorescence was collected at λ_{ex} = 559 nm and λ_{em} = 580-650 nm, respectively. The cell nuclei was stained by Hoechst 33342 and fluorescence collected at λ_{ex} = 405 nm and λ_{em} = 450-480 nm. Scale bar = 20 µm. N = 3.

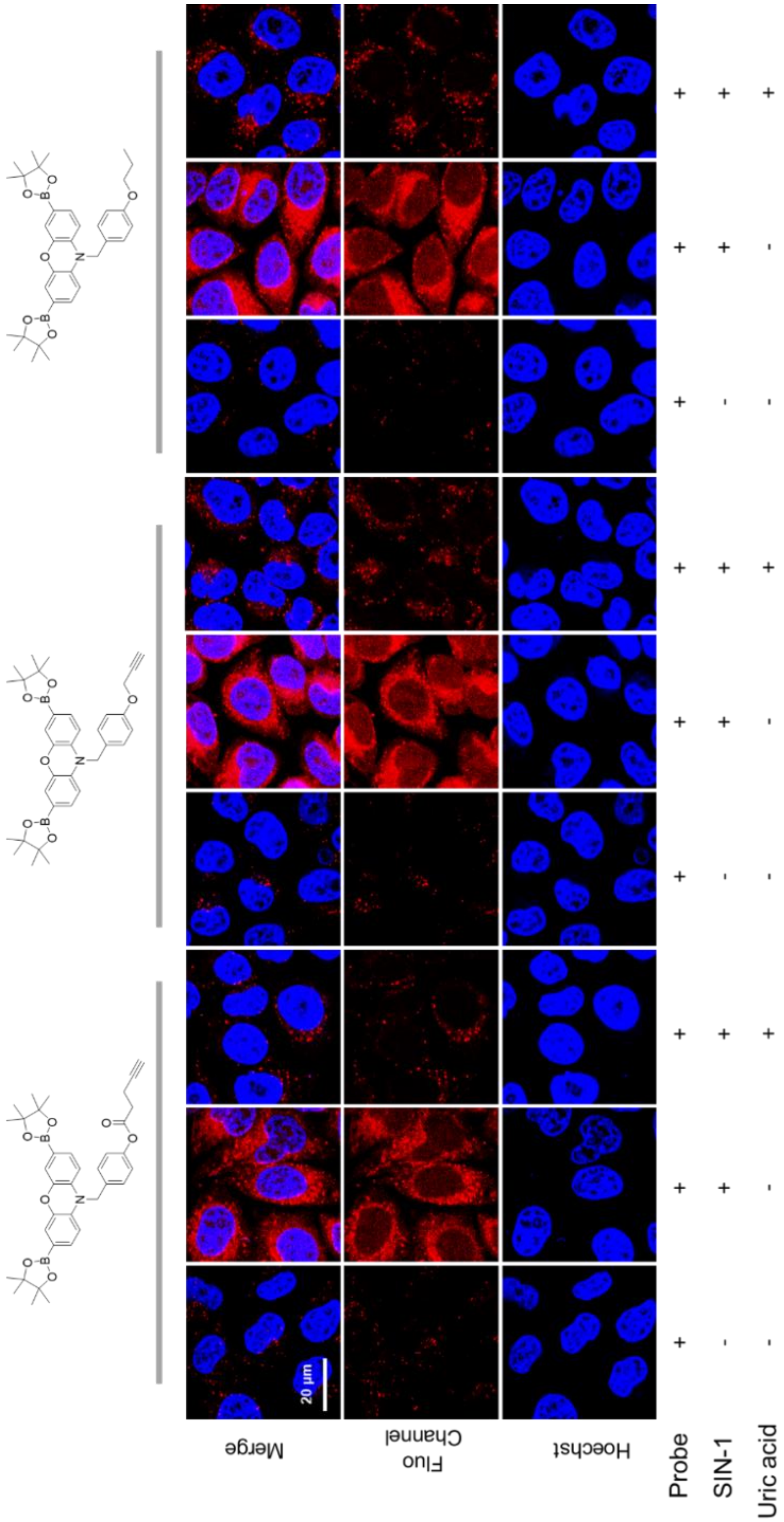

Figure S12. Confocal imaging of HeLa cells loaded with **2** (20 µM, 30 min), **5** (20 µM, 30 min) or **6** (20 µM, 30 min), then treated with SIN-1 (500 µM, 30 min) or uric acid (100 µM, 2 h) and SIN-1 (500 µM, 30 min), as indicated. Probe fluorescence was collected at λ_{ex} = 559 nm and λ_{em} = 580- 650 nm, respectively. The cell nuclei was stained by Hoechst33342 and fluorescence collected at λ_{ex} = 405 nm and λ_{em} = 450-480 nm. Scale bar = 20 µm. N = 3.

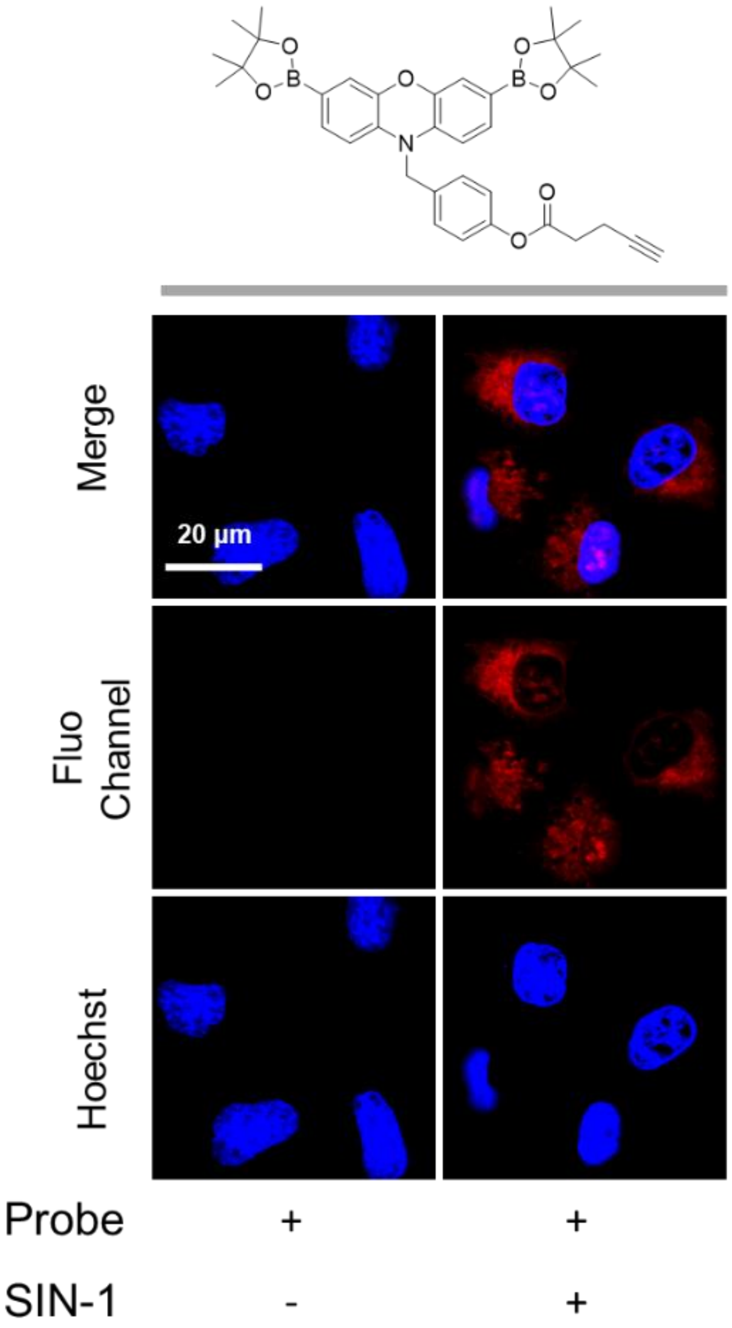

Figure S13. Confocal imaging of A549 cells were loaded with probe **6** (20 µM, 30 min) without and with SIN-1 (500 µM, 30 min) as indicated. Probe fluorescence was collected at λ_{ex} = 559 nm and λ_{em} = 580- 650 nm, respectively. The cell nuclei were stained by Hoechst 33342 and fluorescence collected at λ_{ex} = 405 nm and λ_{em} =450-480 nm. Scale bar = 20 µm. N = 3.

A solution of either probe **7** or **8** in PBS buffer (5 µM, pH = 7.40) and a solution of $ONOO^-$ (c = 45 mM) were separately infused via syringe pumps into the mass spectrometer at a flow rate of 3 µL/min. The reagents reacted after infusion to the mass spectrometer and provided the results given below

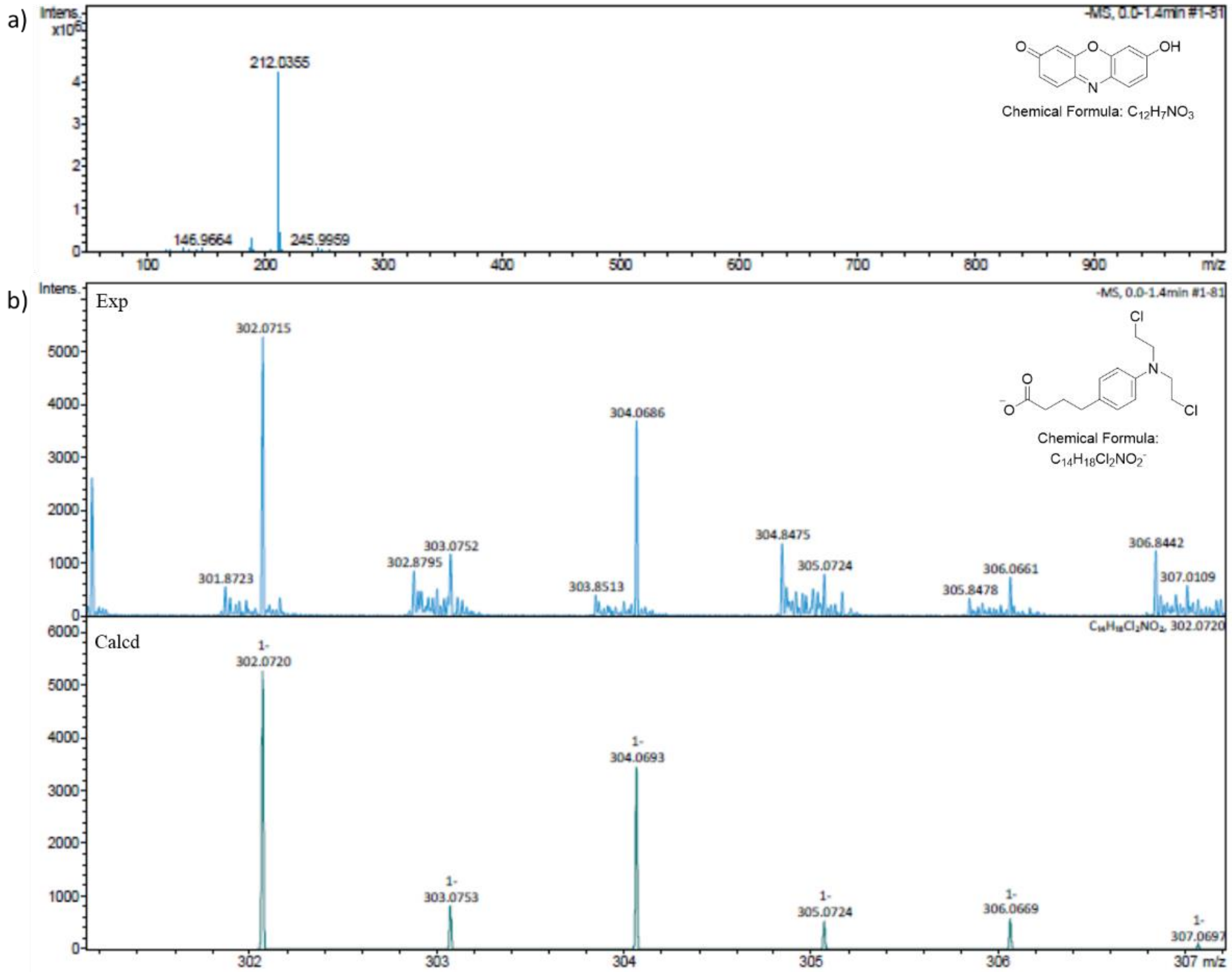

Figure S14. MS data revealed that **7** upon reaction with $ONOO^-$ releases (a) resorufin and (b) chlorambucil.

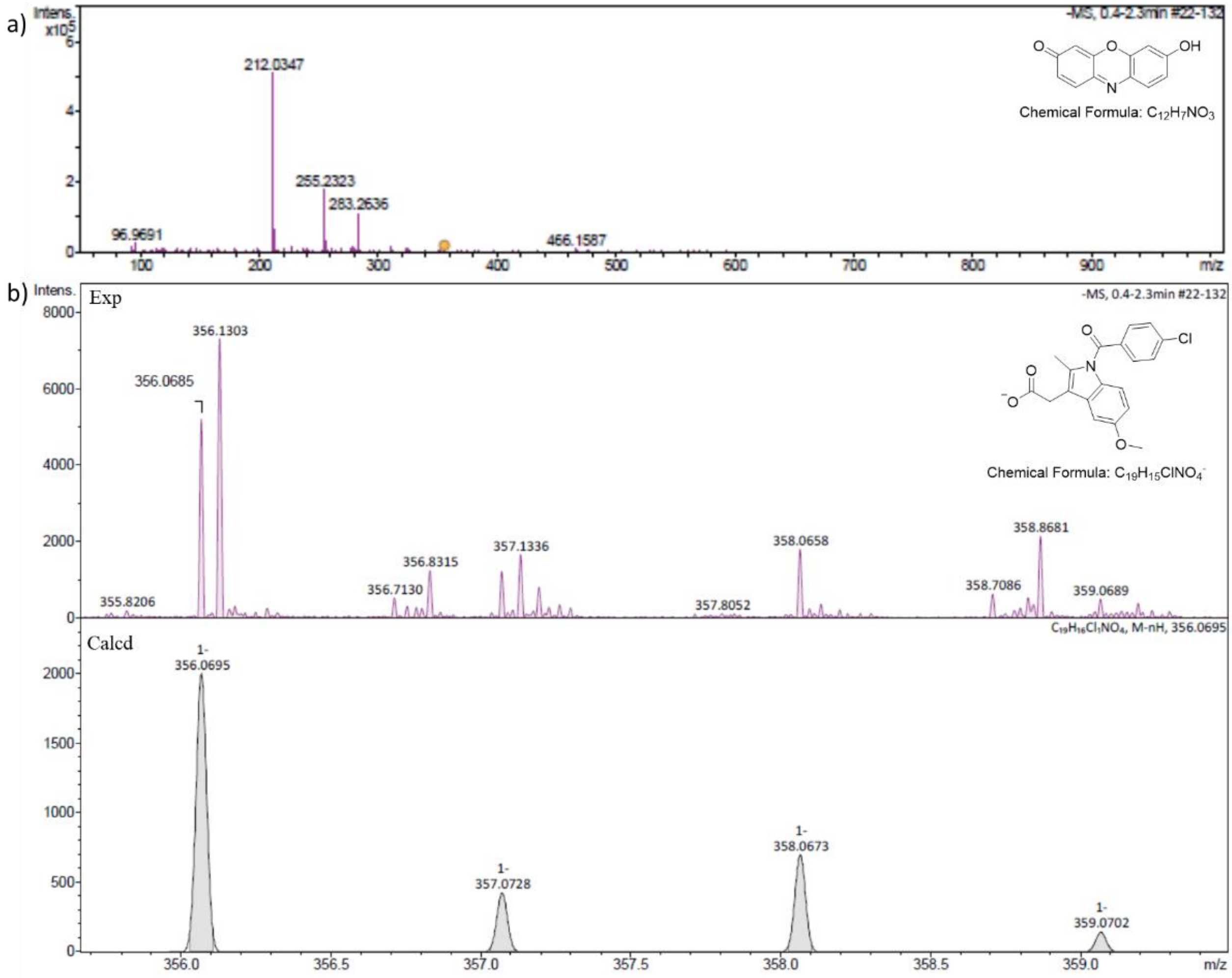

Figure S15. MS data revealed that **8** upon reaction with $ONOO^-$ releases (a) resorufin and (b) chlorambucil.

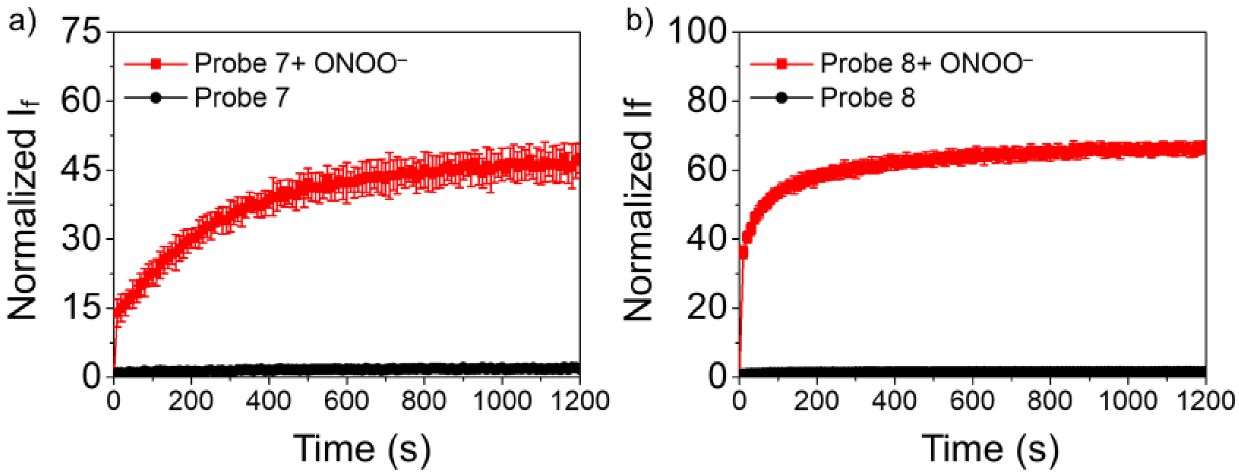

Figure S16. Fluorescence intensity changes over time for probes a) **7** (5 µM), b) **8** (5 µM) in the presence of $ONOO^-$ (50 µM) in PBS buffer at 25 °C. Fluorescence intensities were measured with λex = 550 nm and max λem = 590 nm on a M5 microplate reader (Molecular Device, USA).

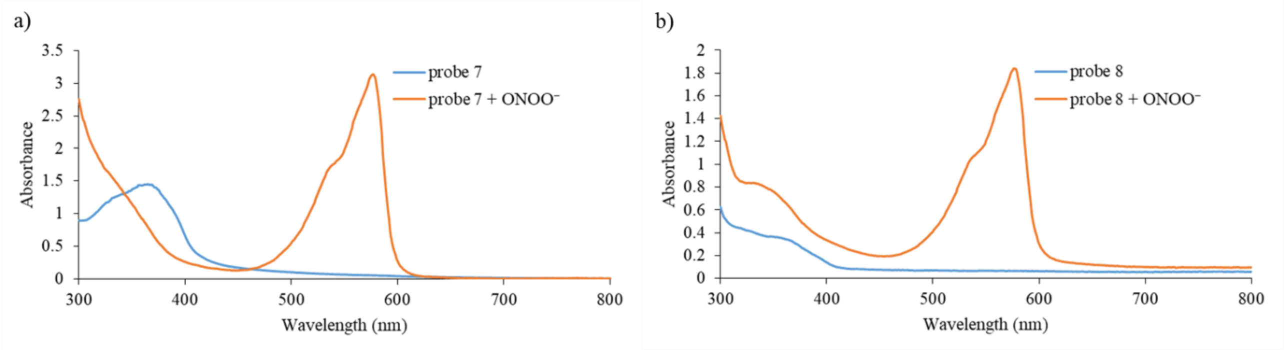

Figure S17. UV spectra of a) **7** and b) **8** with and without $ONOO^-$ (excess) in PBS buffer 52 % MeOH : H_2O, pH = 8.2 at 25 °C measured on a BMG Labtech CLARIOstar® plate reader.

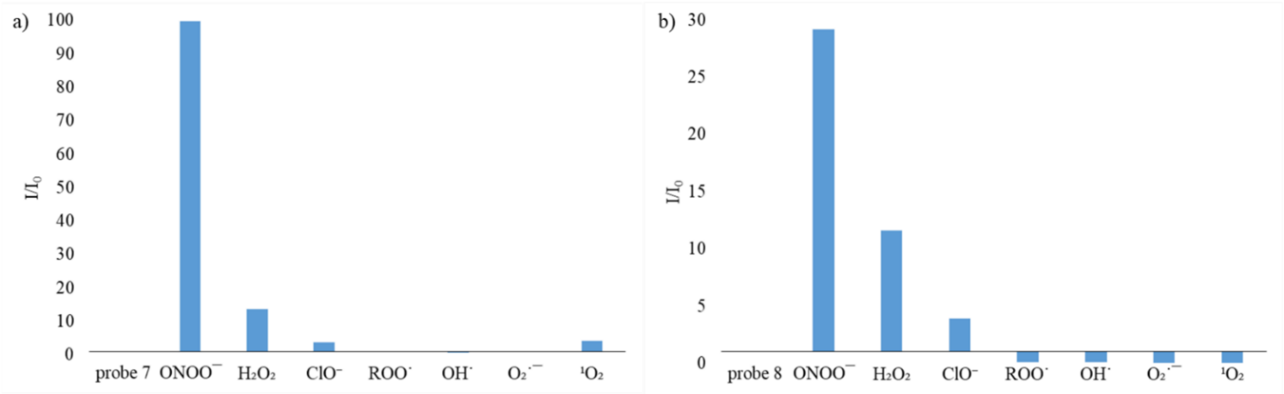

Figure S18. Selectivity data for a) **7** (500 nM), and e) **8** (500 nM) in the presence of $ONOO^-$ (50 µM), OH· (500 µM), $O_2^{\cdot -}$ (500 µM), $^1O_2$ (500 µM) after 5 min. H_2O_2 (1 mM), ROO· (500 µM) and ClO^- (500 µM) were measured after 30 min. The data was obtained in PBS buffer 52 % MeOH : H_2O, pH = 8.2 at 25 °C at λ_{ex} = 550 (bandwidth: 15) nm and max λ_{em} = 590 nm on a BMG Labtech CLARIOstar® plate reader.

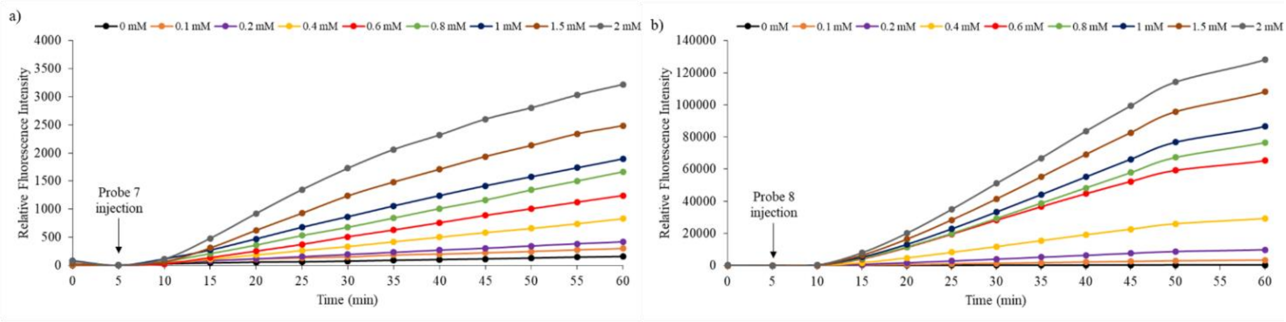

Figure S19. Fluorescence intensity changes over time for a) **7** (500 nM) and b) **8** (500 nM) in the presence of H_2O_2 (0.1, 0.2, 0.4, 0.6, 0.8, 1, 1.5, 2 mM) in PBS buffer 52 % MeOH : H_2O, pH = 8.2 at 25 °C. Fluorescence intensities were measured with λ_{ex} = 550 (bandwidth: 15) nm and max λ_{em} = 590 nm on a BMG Labtech CLARIOstar® plate reader.

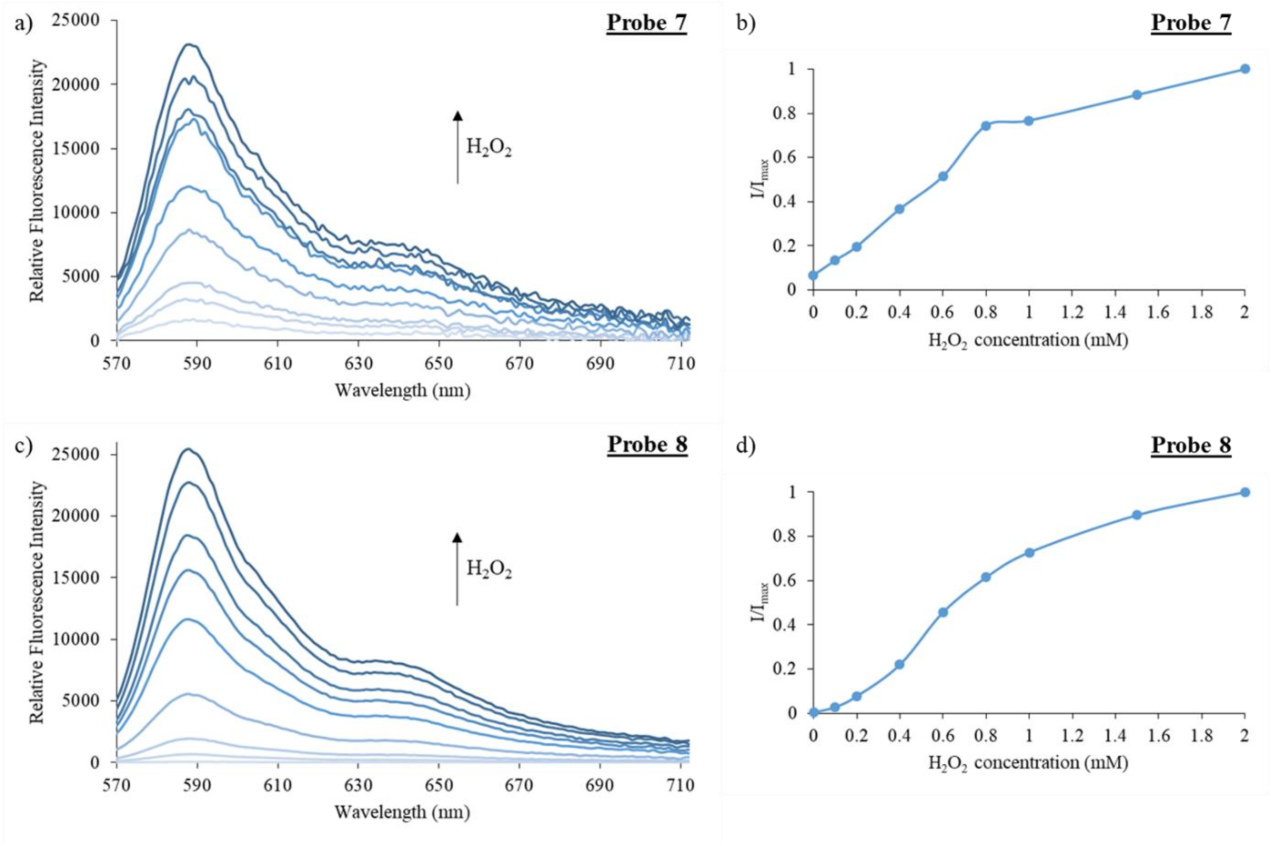

Figure S20. Emission spectra and dose dependence curve (I/I_{max}) for a) & b) **7** (500 nM) and c) & d) **8** (500 nM) in the presence of H_2O_2 (0.1, 0.2, 0.4, 0.6, 0.8, 1, 1.5, 2 mM) after 1 h in PBS buffer 52 % MeOH : H_2O, pH = 8.2 at 25 °C. Fluorescence intensities were measured with λ_{ex} = 550 (bandwidth: 15) nm and max λ_{em} = 590 nm on a BMG Labtech CLARIOstar® plate reader.

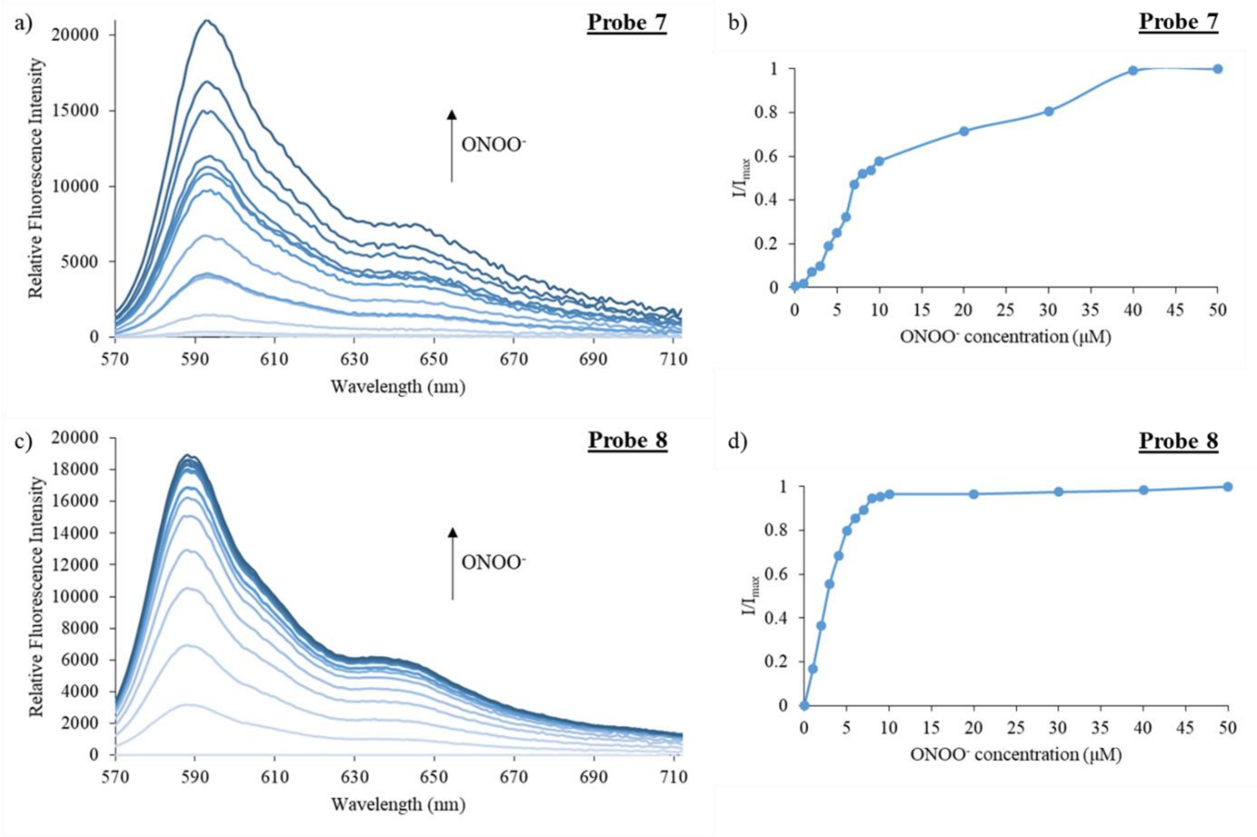

Figure S21. Emission spectra and dose dependence curve (I/I_{max}) for a) & b) **7** (500 nM) and c) & d) **8** (500 nM) in the presence of $ONOO^-$ (1, 2, 3, 4, 5, 6, 7, 8, 9, 10, 20, 30, 40, 50 µM) in PBS buffer 52 % MeOH : H_2O, pH = 8.2 at 25 °C. Fluorescence intensities were measured with λ_{ex} = 550 (bandwidth: 15) nm and max λ_{em} = 590 nm on a BMG Labtech CLARIOstar® plate reader.

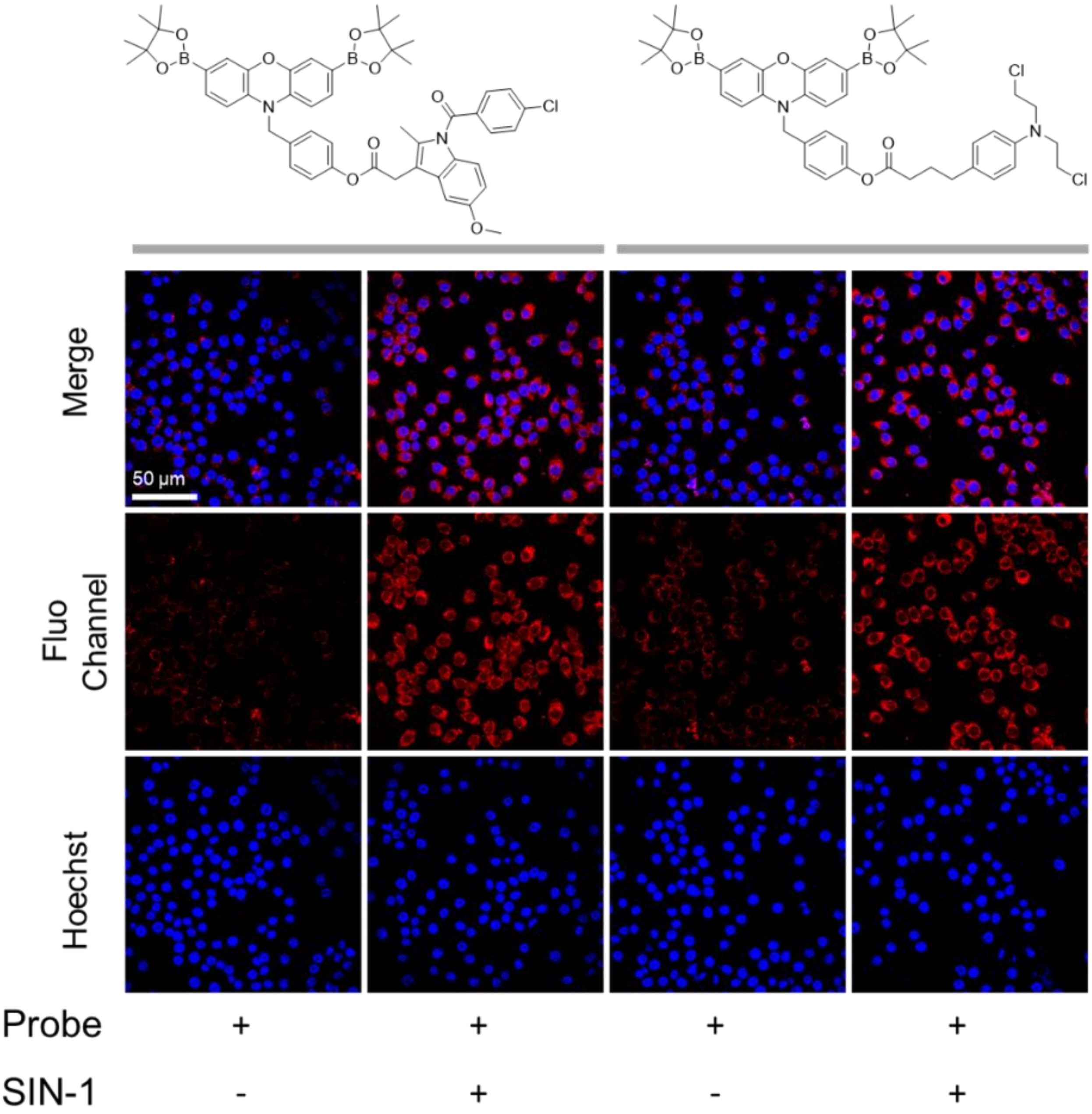

Figure S22. Confocal imaging: RAW 264.7 macrophages were loaded with **7** (20 μM, 30 min), and **8** (20 μM, 30 min) and treated with SIN-1 (500 μM, 30 min), as indicated. Probe fluorescence was collected at λ_{ex} = 559 nm and λ_{em} = 580- 650 nm, respectively. The cell nuclei was stained by Hoechst 33342 and fluorescence collected at λ_{ex} = 405 nm and λ_{em} = 450-480 nm. Scale bar = 50 μM, N = 3.

4. NMR Spectra

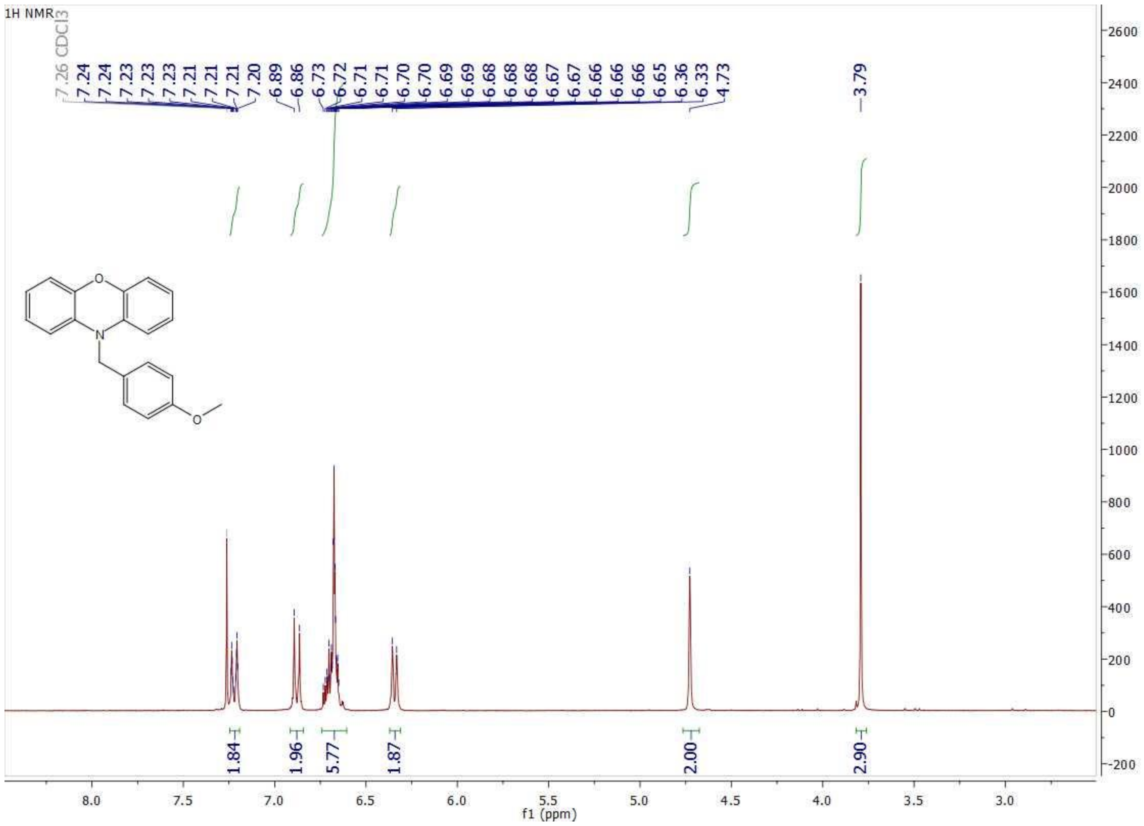

^{1}H NMR of **10-(4-methoxybenzyl)-10*H*-phenoxazine**

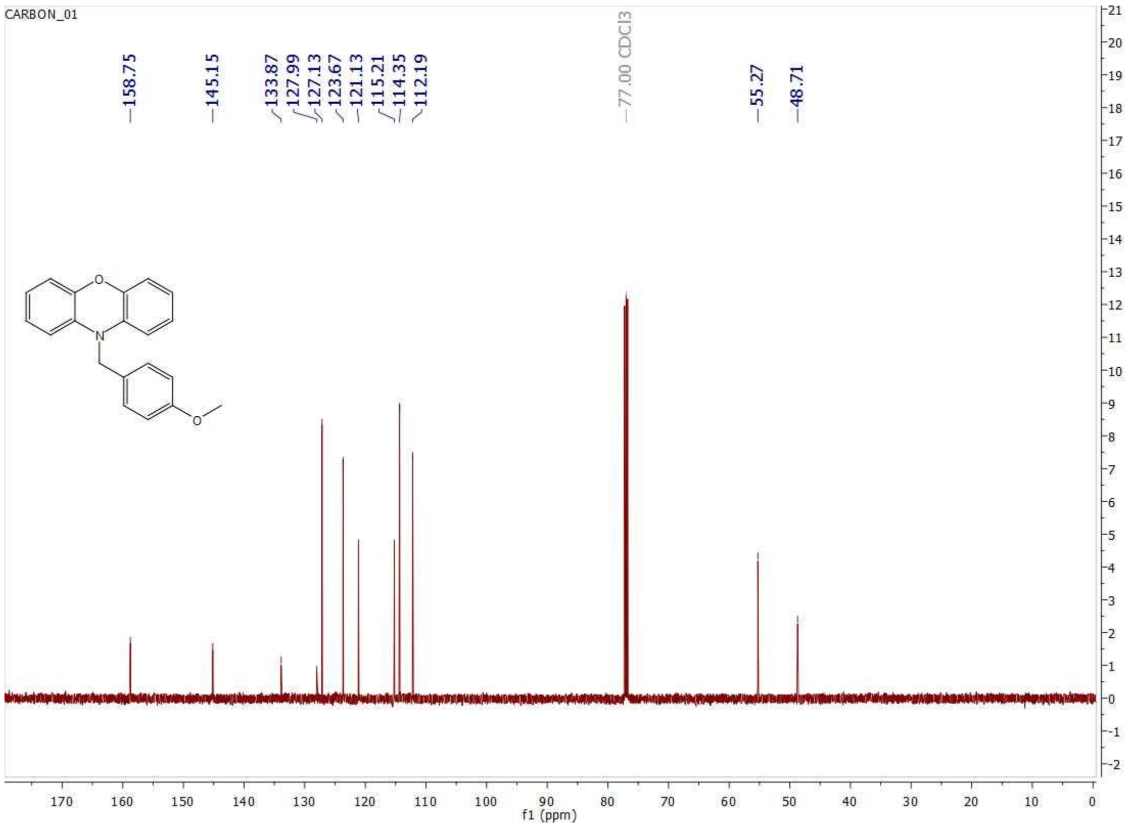

^{13}C NMR of **10-(4-methoxybenzyl)-10*H*-phenoxazine**

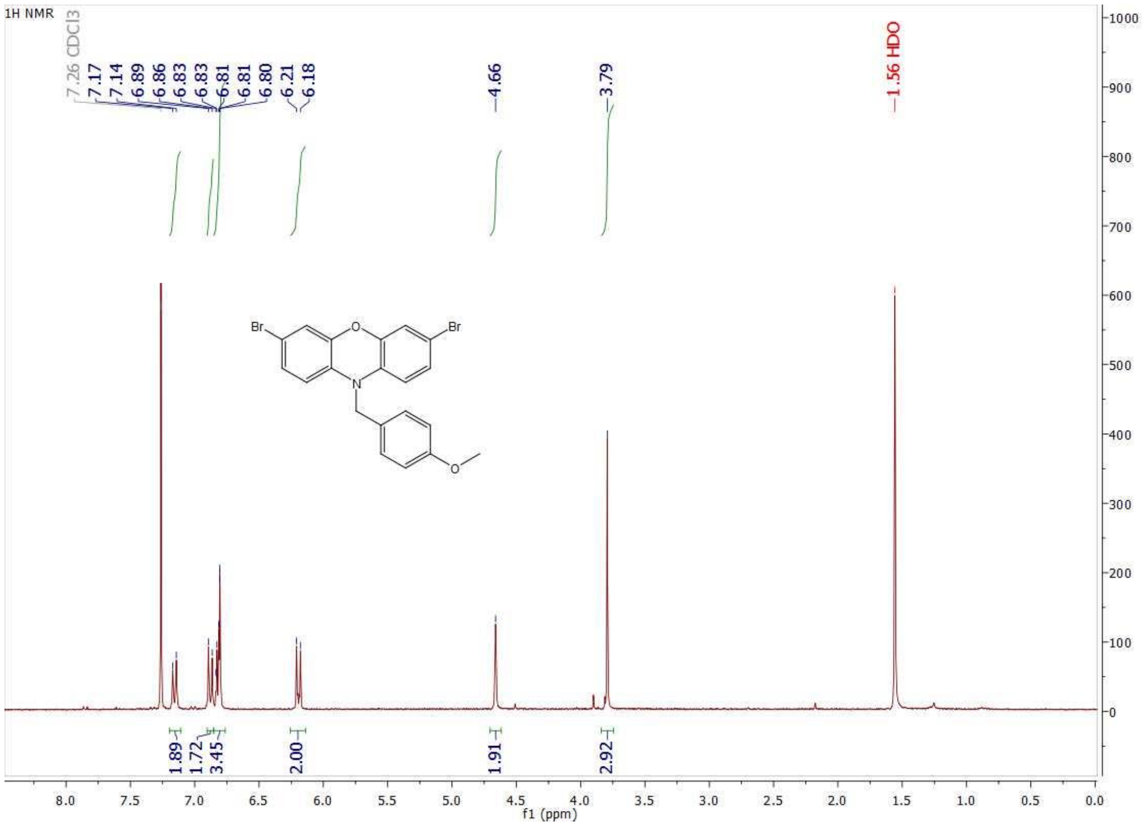

^{1}H NMR of **3,7-dibromo-10-(4-methoxybenzyl)-10*H*-phenoxazine**

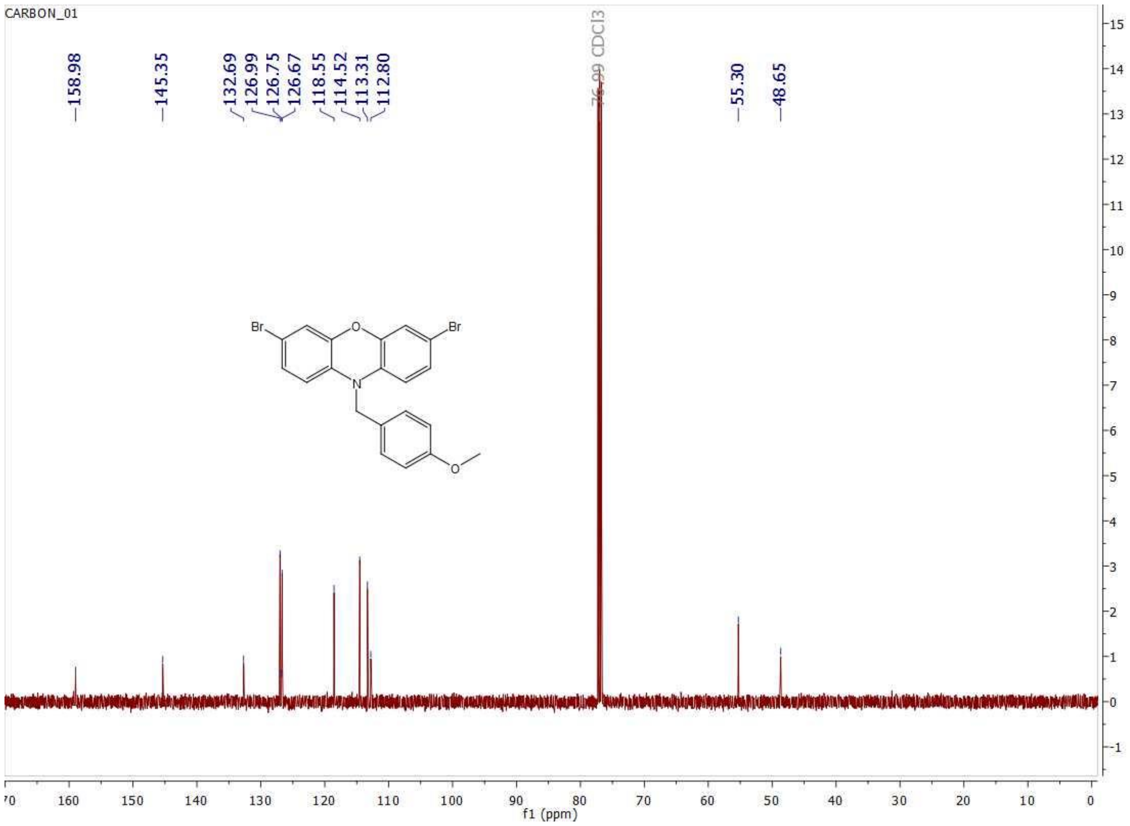

^{13}C NMR of **3,7-dibromo-10-(4-methoxybenzyl)-10*H*-phenoxazine**

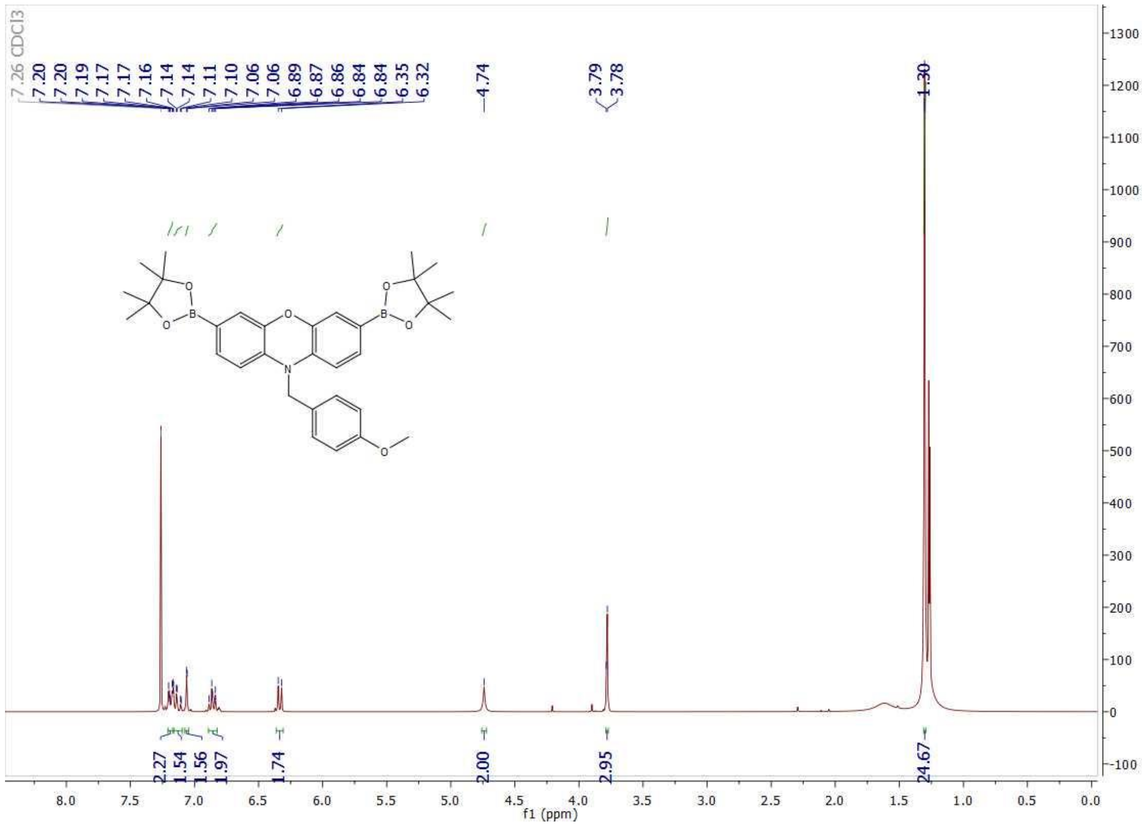

^{1}H NMR of **10-(4-methoxybenzyl)-3,7-bis(4,4,5,5-tetramethyl-1,3,2-dioxaborolan-2-yl)-10*H*-phenoxazine**

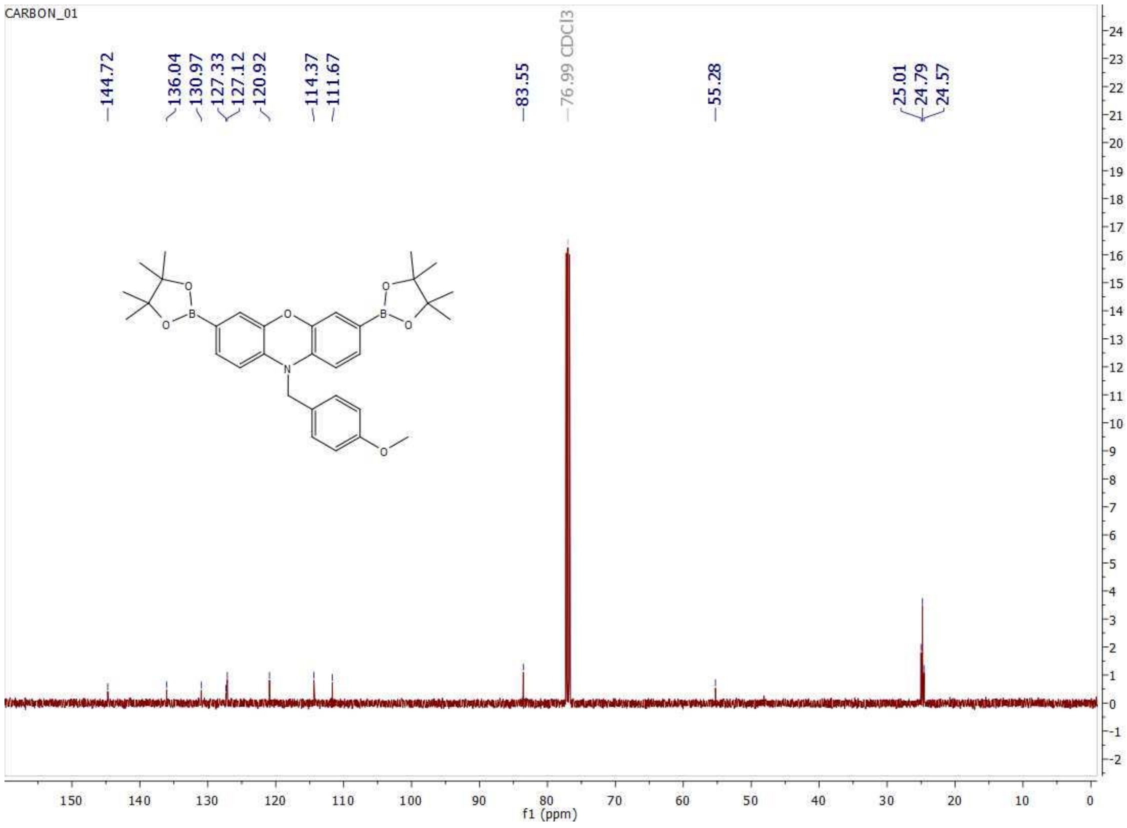

^{13}C NMR of **10-(4-methoxybenzyl)-3,7-bis(4,4,5,5-tetramethyl-1,3,2-dioxaborolan-2-yl)-10*H*-phenoxazine**

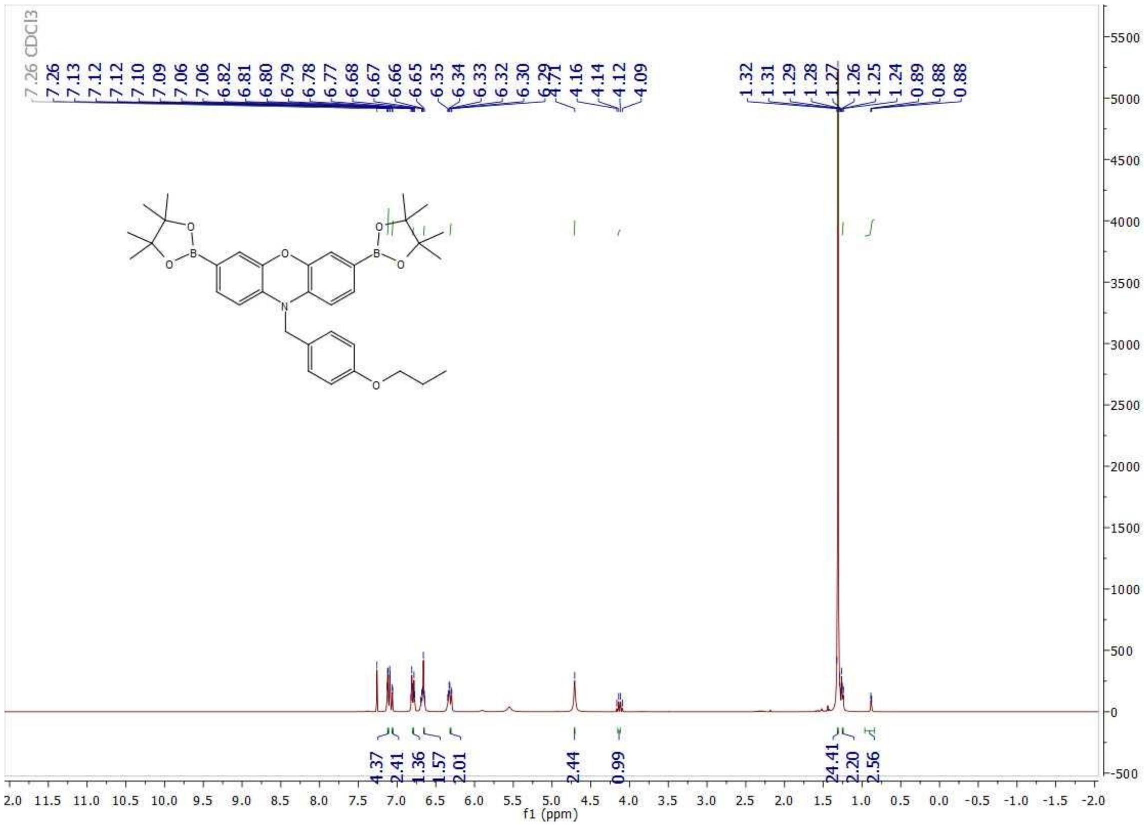

^{1}H NMR of **10-(4-propoxybenzyl)-3,7-bis(4,4,5,5-tetramethyl-1,3,2-dioxaborolan-2-yl)-10*H*-phenoxazine**

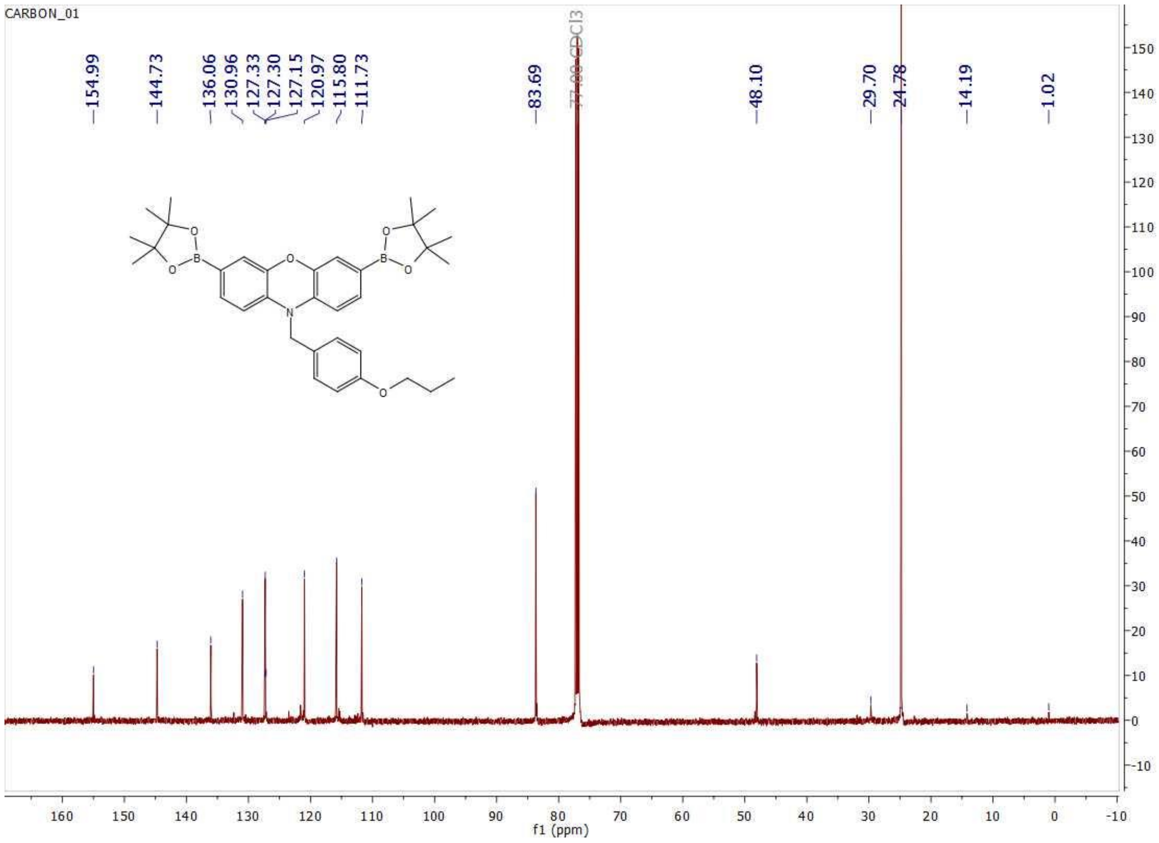

^{13}C NMR of **10-(4-propoxybenzyl)-3,7-bis(4,4,5,5-tetramethyl-1,3,2-dioxaborolan-2-yl)-10*H*-phenoxazine**

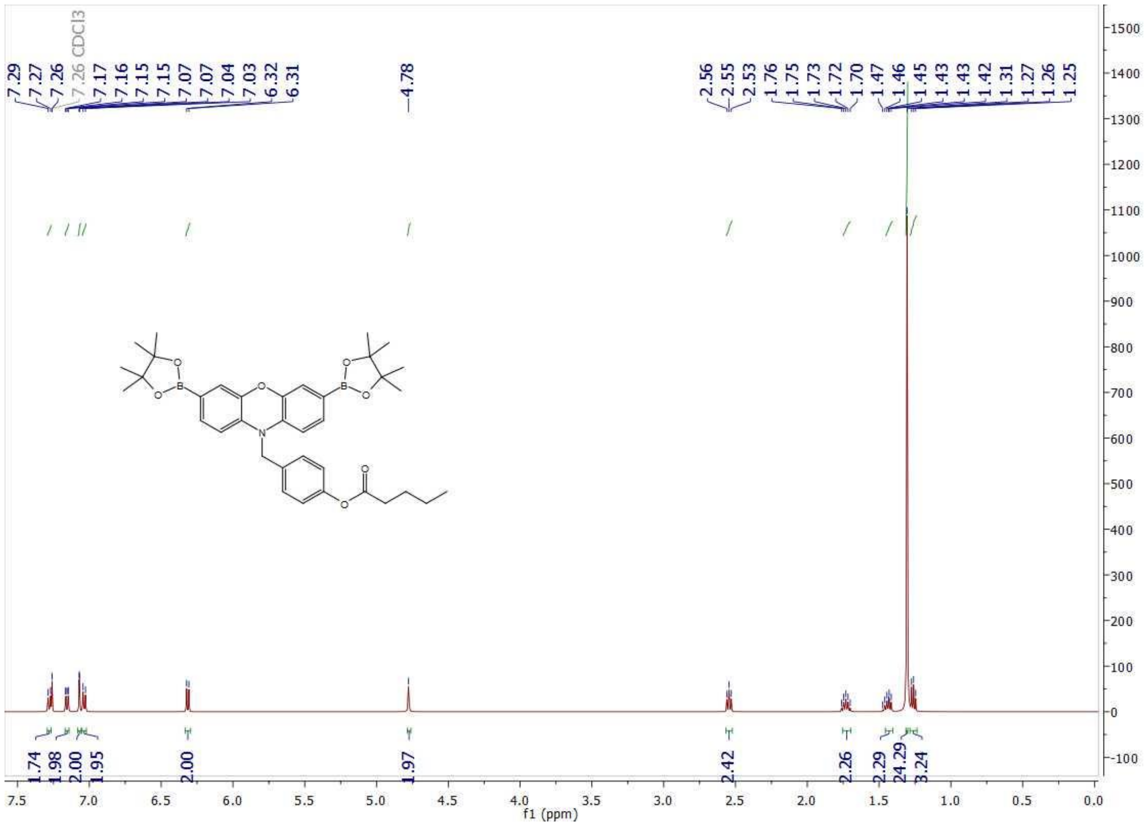

^{1}H NMR of **4-((3,7-bis(4,4,5,5-tetramethyl-1,3,2-dioxaborolan-2-yl)-10*H*-phenoxazin-10-yl)methyl)phenyl pentanoate**

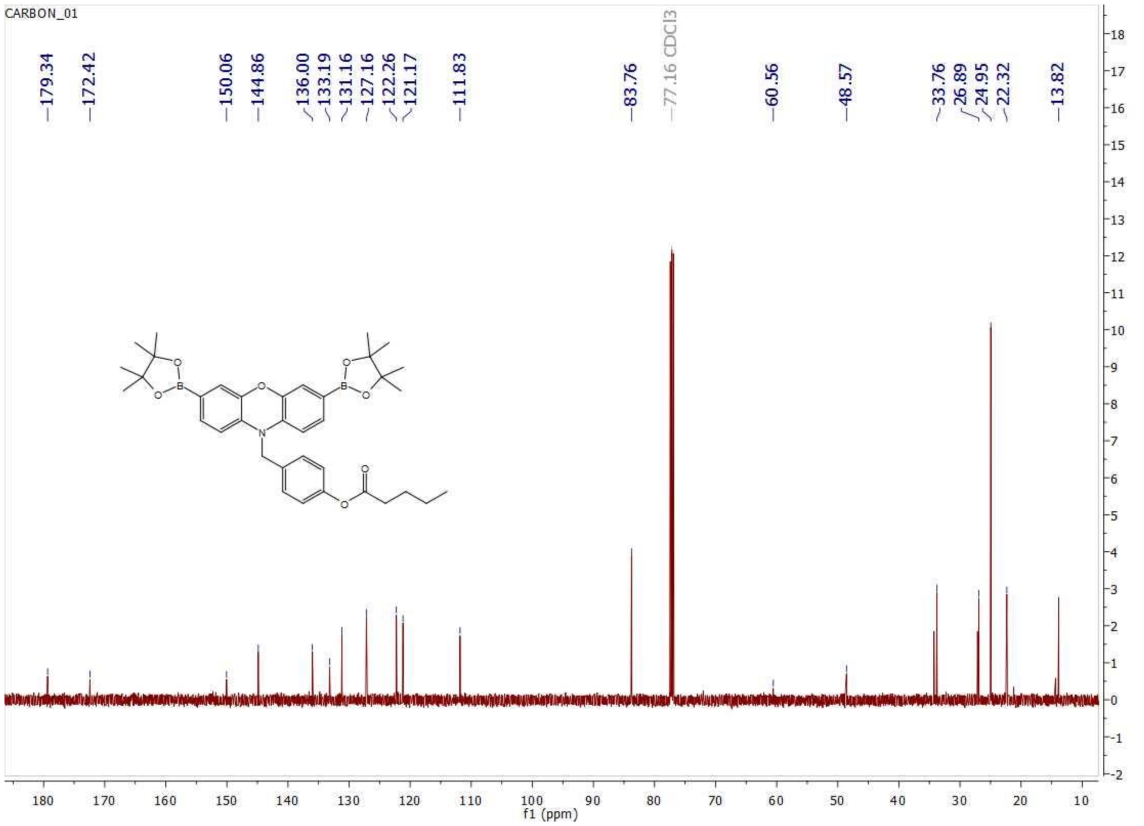

^{13}C NMR of **4-((3,7-bis(4,4,5,5-tetramethyl-1,3,2-dioxaborolan-2-yl)-10*H*-phenoxazin-10-yl)methyl)phenyl pentanoate**

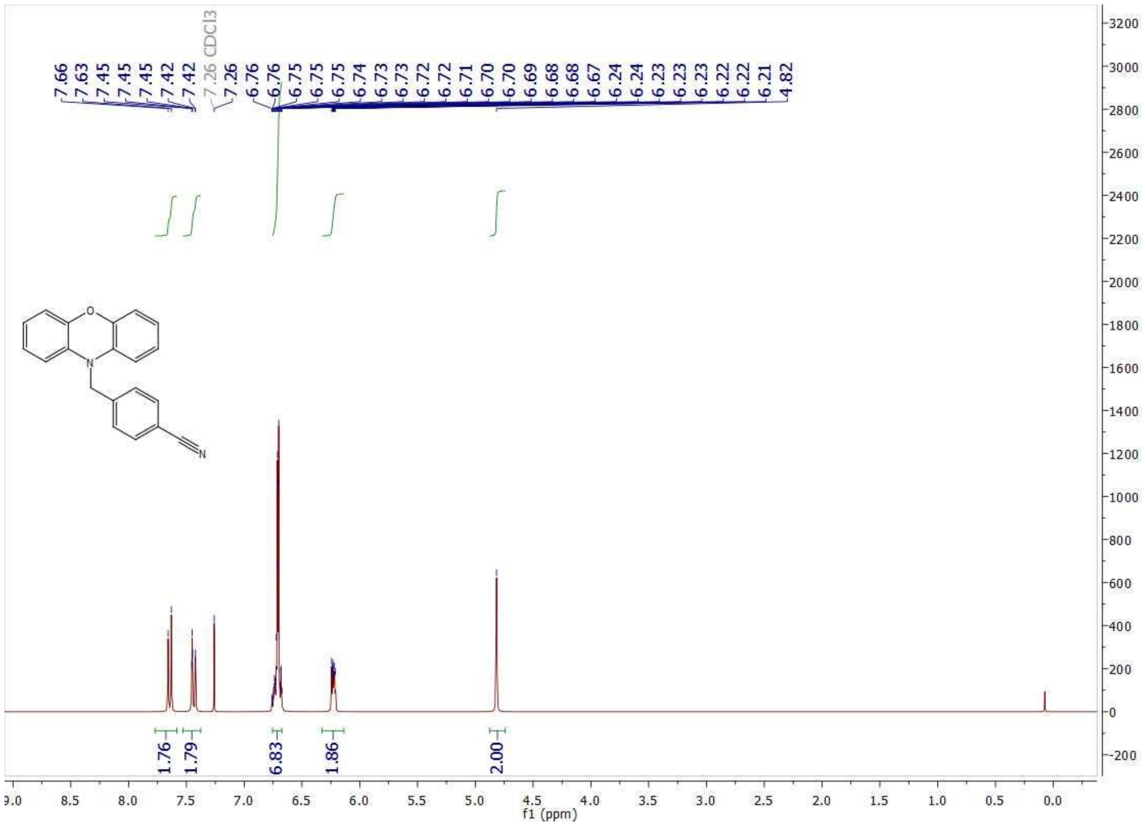

^{1}H NMR of **4-((10*H*-phenoxazin-10-yl)methyl)benzonitrile**

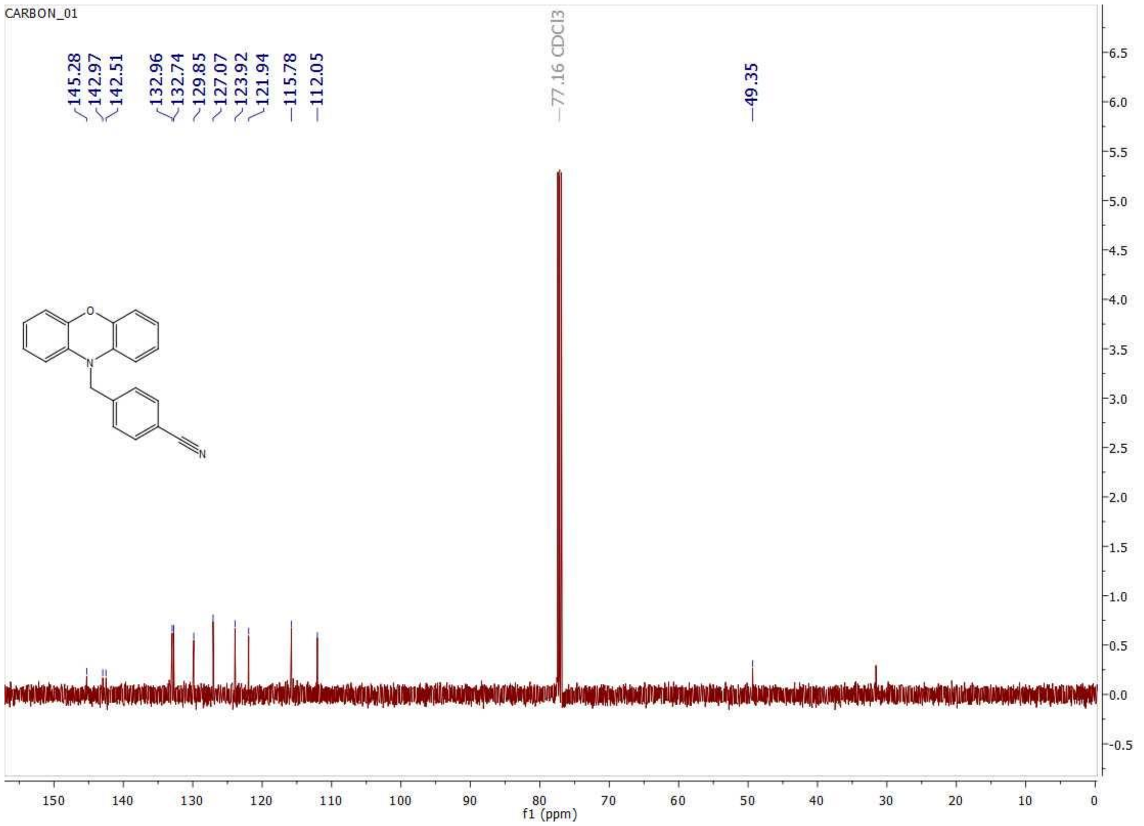

^{13}C NMR of **4-((10*H*-phenoxazin-10-yl)methyl)benzonitrile**

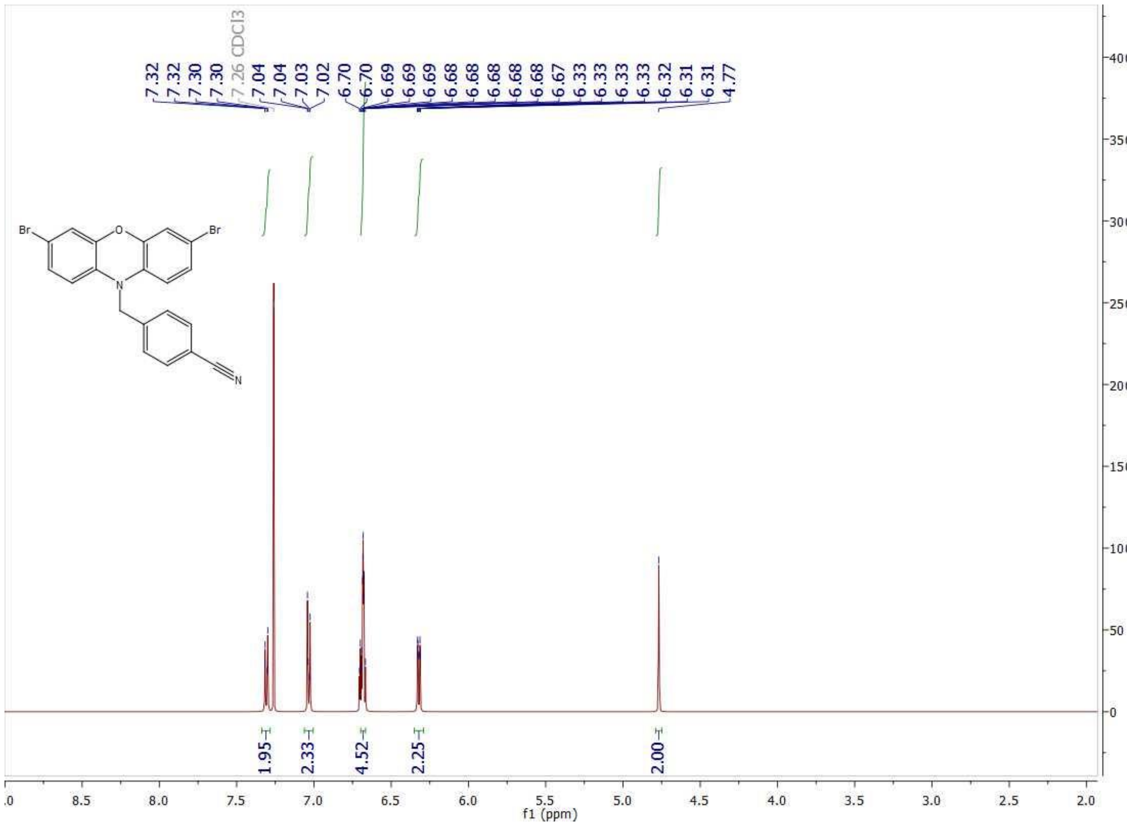

^{1}H NMR of **4-((3,7-dibromo-10*H*-phenoxazin-10-yl)methyl)benzonitrile**

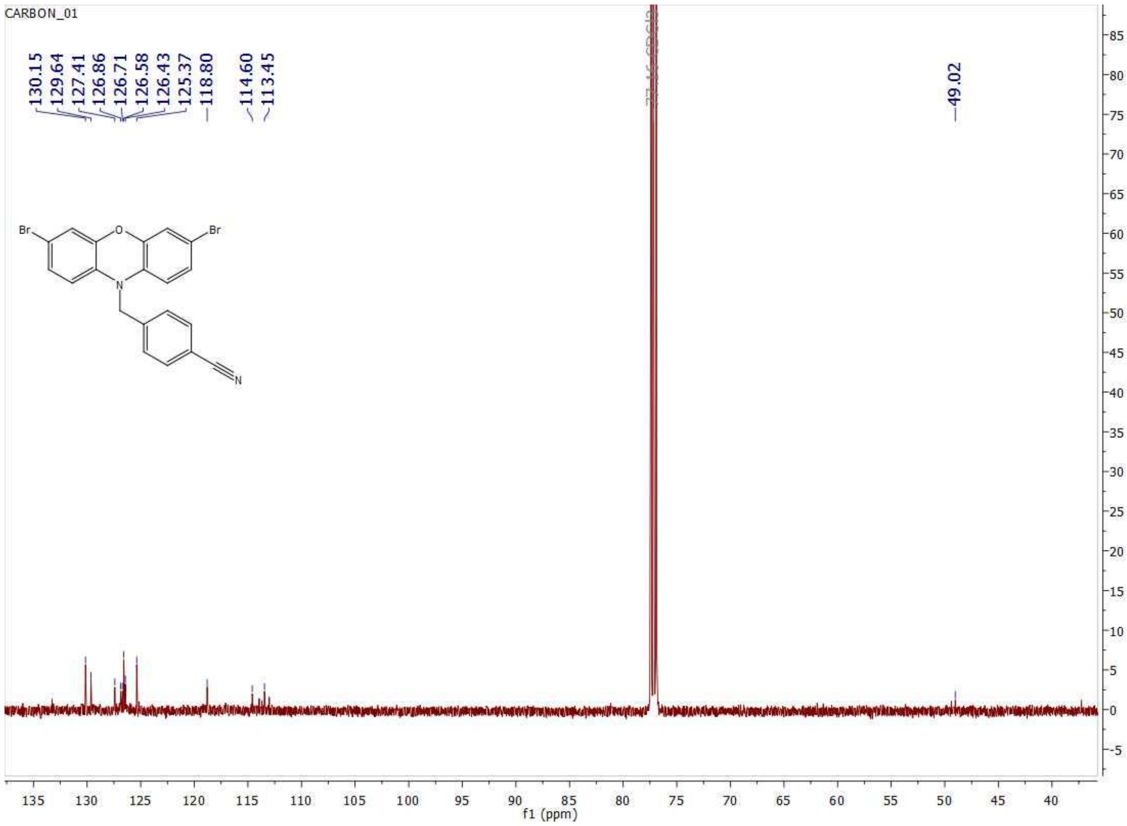

^{13}C NMR of **4-((3,7-dibromo-10*H*-phenoxazin-10-yl)methyl)benzonitrile**

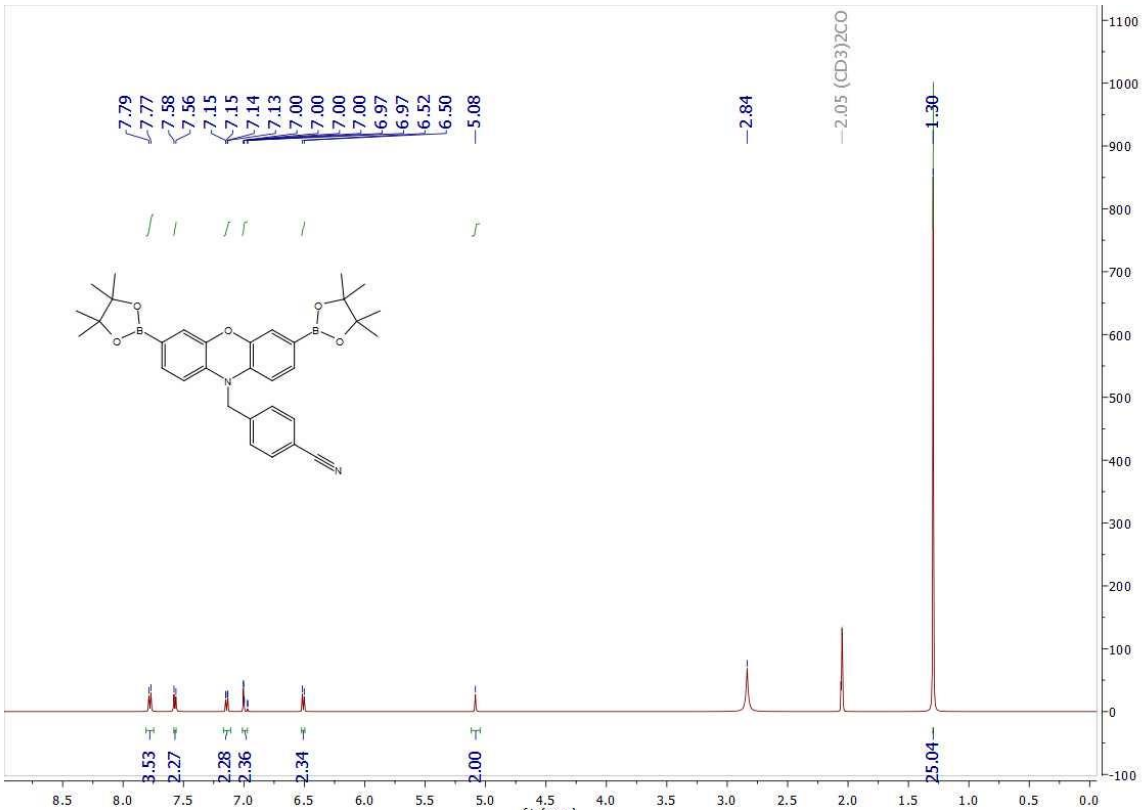

^{1}H NMR of **4-((3,7-bis(4,4,5,5-tetramethyl-1,3,2-dioxaborolan-2-yl)-10*H*-phenoxazin-10-yl)methyl)benzonitrile**

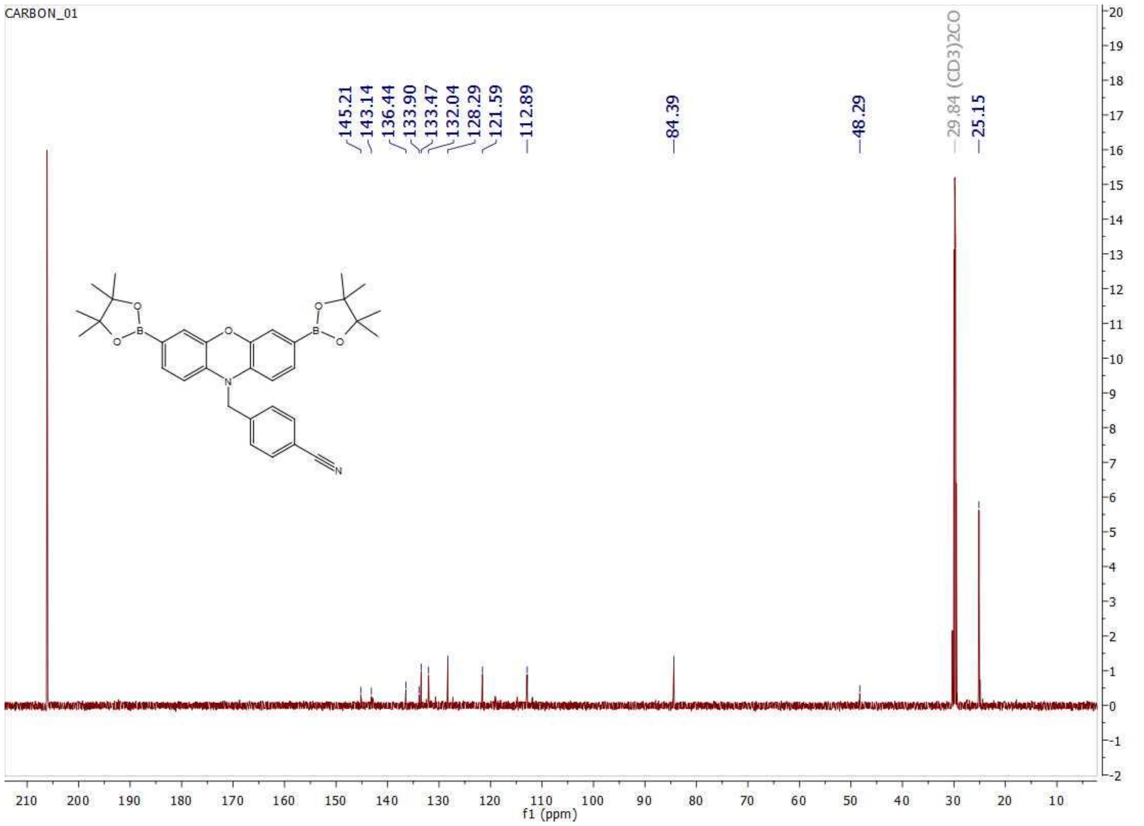

^{13}C NMR of **4-((3,7-bis(4,4,5,5-tetramethyl-1,3,2-dioxaborolan-2-yl)-10*H*-phenoxazin-10-yl)methyl)benzonitrile**

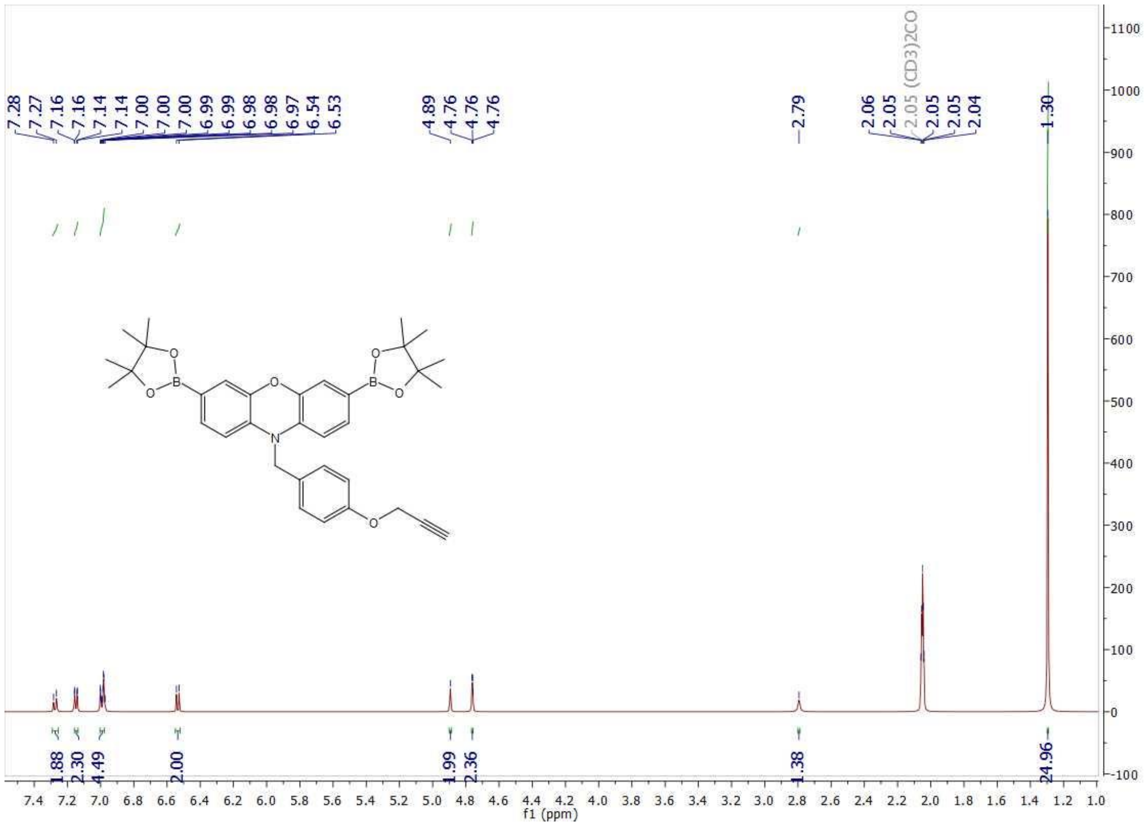

^{1}H NMR of **10-(4-(prop-2-yn-1-yloxy)benzyl)-3,7-bis(4,4,5,5-tetramethyl-1,3,2-dioxaborolan-2-yl)-10*H*-phenoxazine**

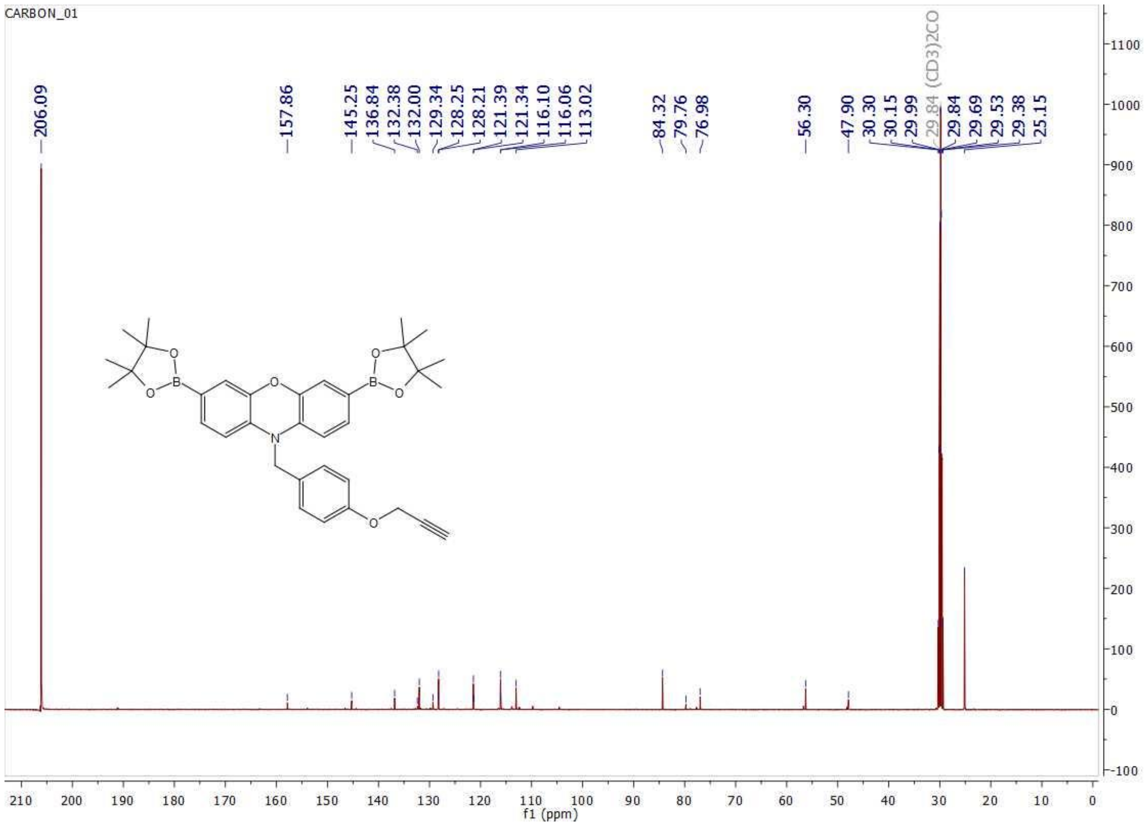

^{13}C NMR of **10-(4-(prop-2-yn-1-yloxy)benzyl)-3,7-bis(4,4,5,5-tetramethyl-1,3,2-dioxaborolan-2-yl)-10*H*-phenoxazine**

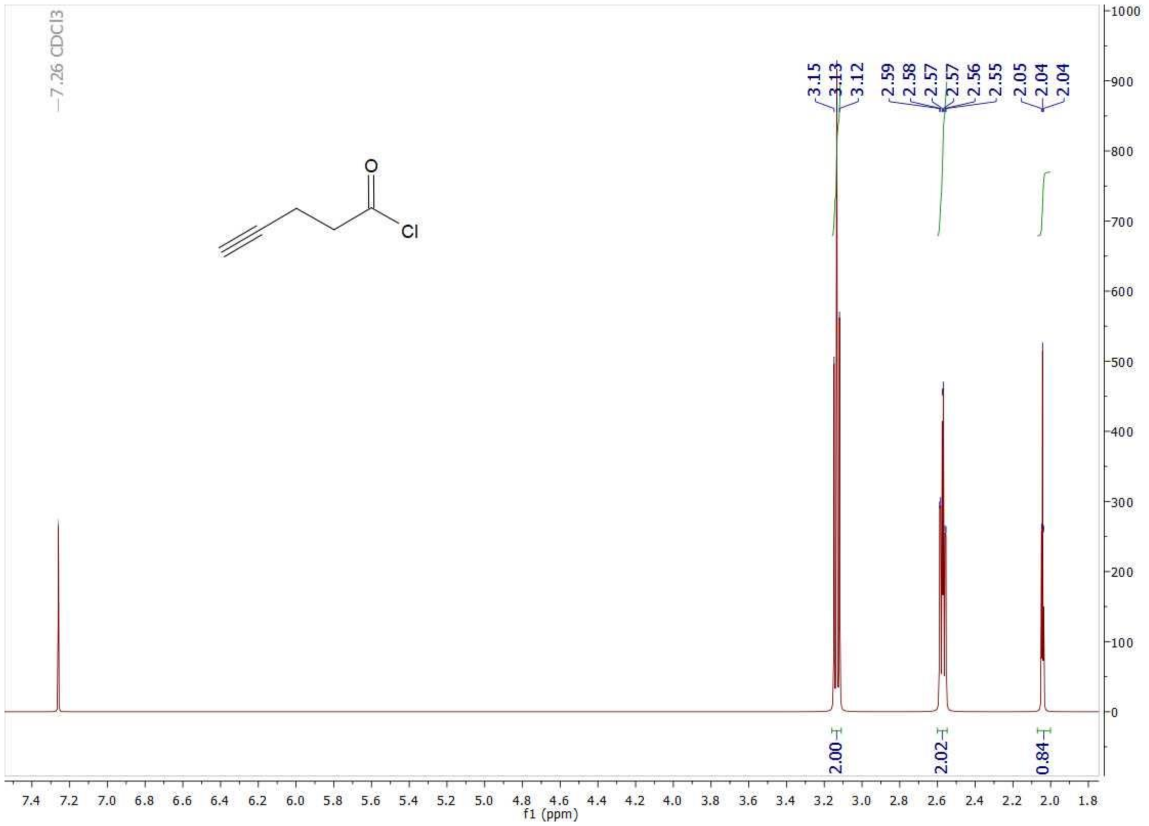

^{1}H NMR of **pent-4-ynoyl chloride**

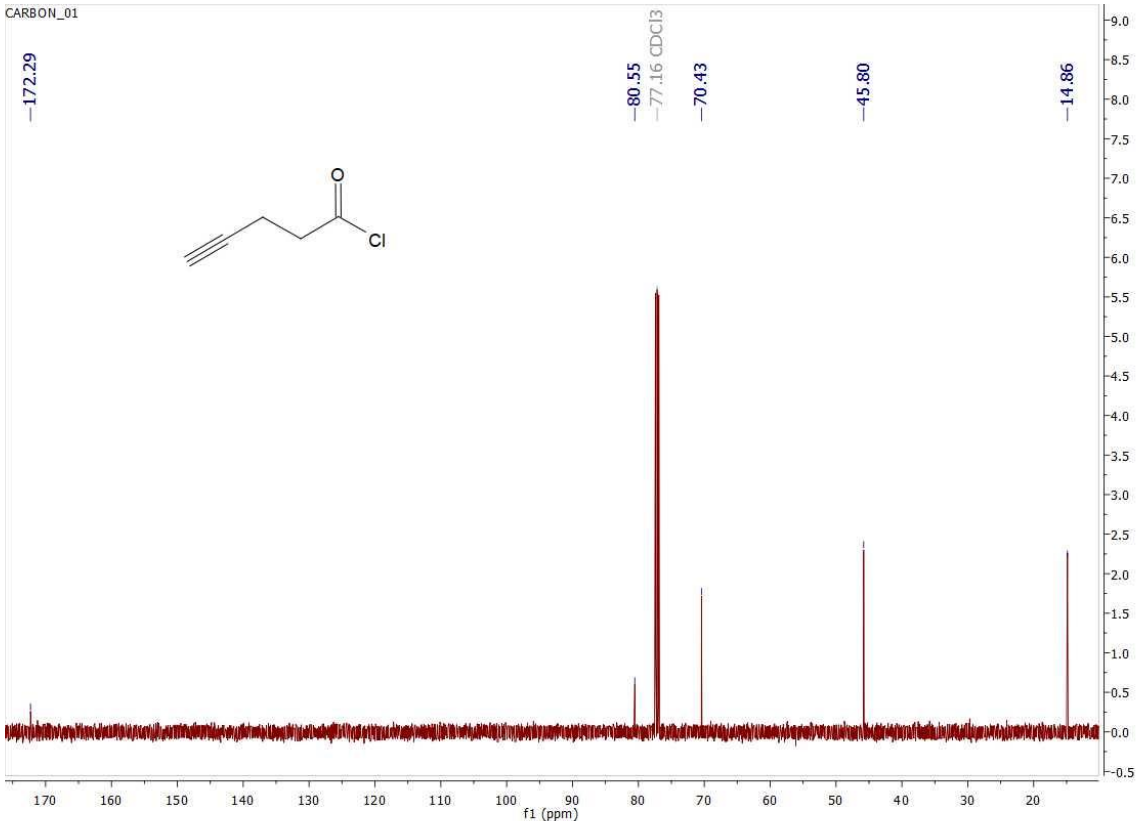

^{13}C NMR of **pent-4-ynoyl chloride**

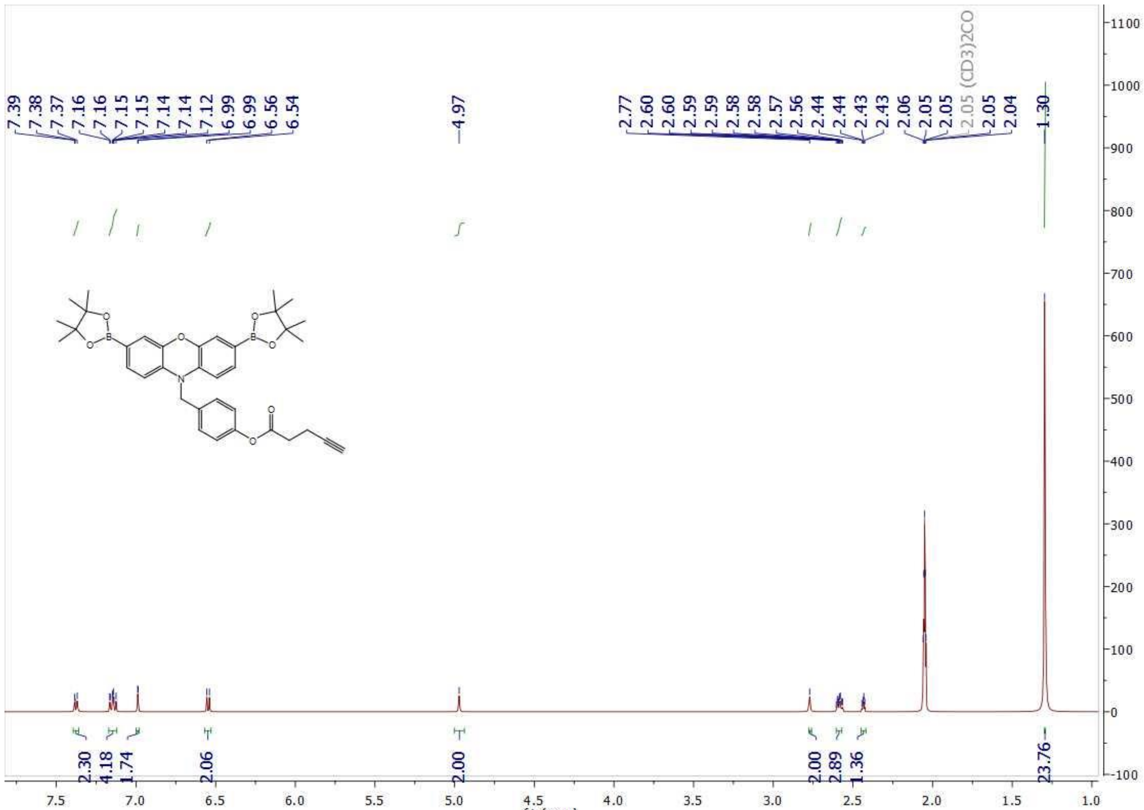

^{1}H NMR of **4-((3,7-bis(4,4,5,5-tetramethyl-1,3,2-dioxaborolan-2-yl)-10*H*-phenoxazin-10-yl)methyl)phenyl pent-4-ynoate**

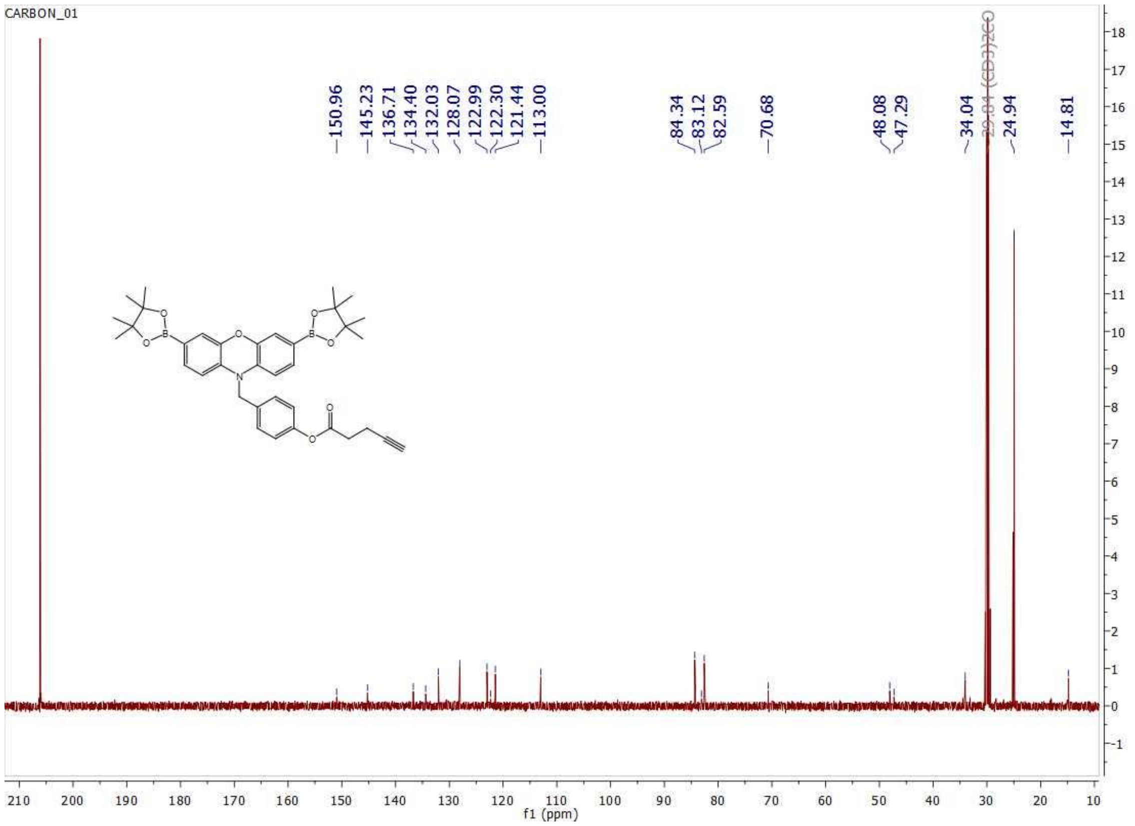

^{13}C NMR of **4-((3,7-bis(4,4,5,5-tetramethyl-1,3,2-dioxaborolan-2-yl)-10*H*-phenoxazin-10-yl)methyl)phenyl pent-4-ynoate**

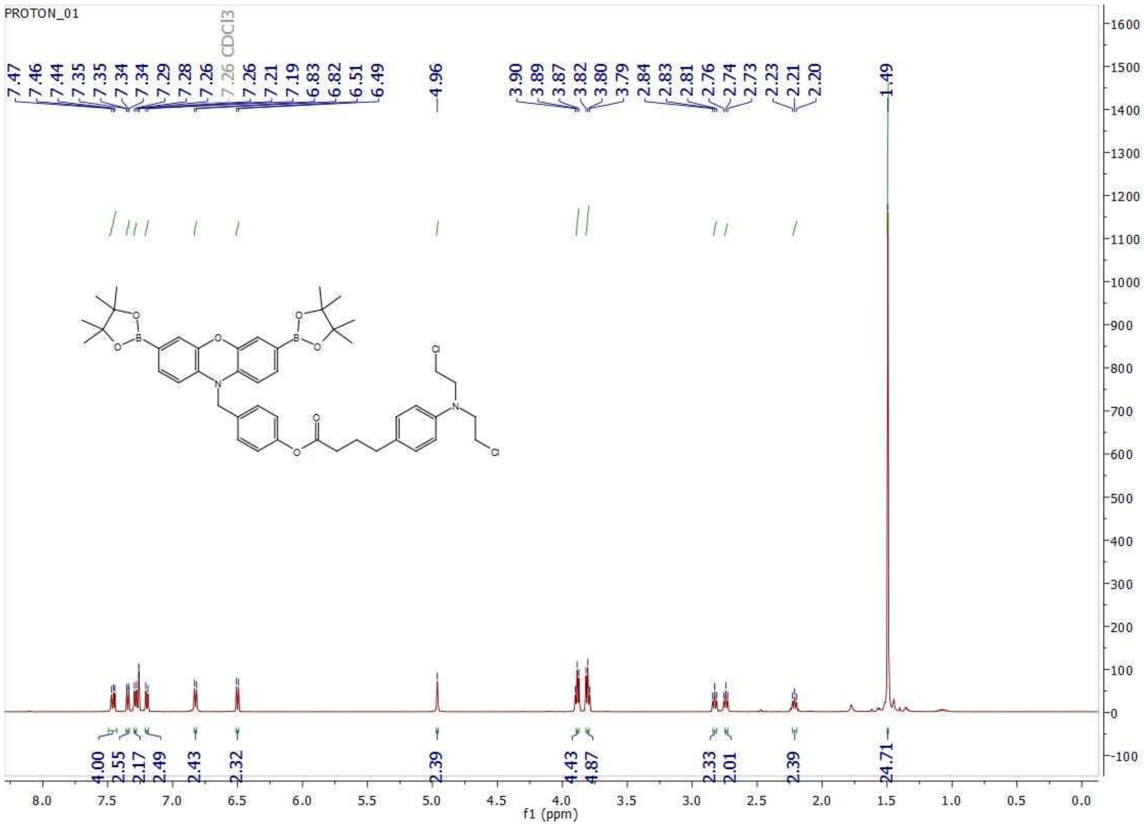

^{1}H NMR of **4-((3,7-bis(4,4,5,5-tetramethyl-1,3,2-dioxaborolan-2-yl)-10*H*-phenoxazin-10-yl)methyl)phenyl 4-(4-(bis(2-chloroethyl)amino) phenyl) butanoate**

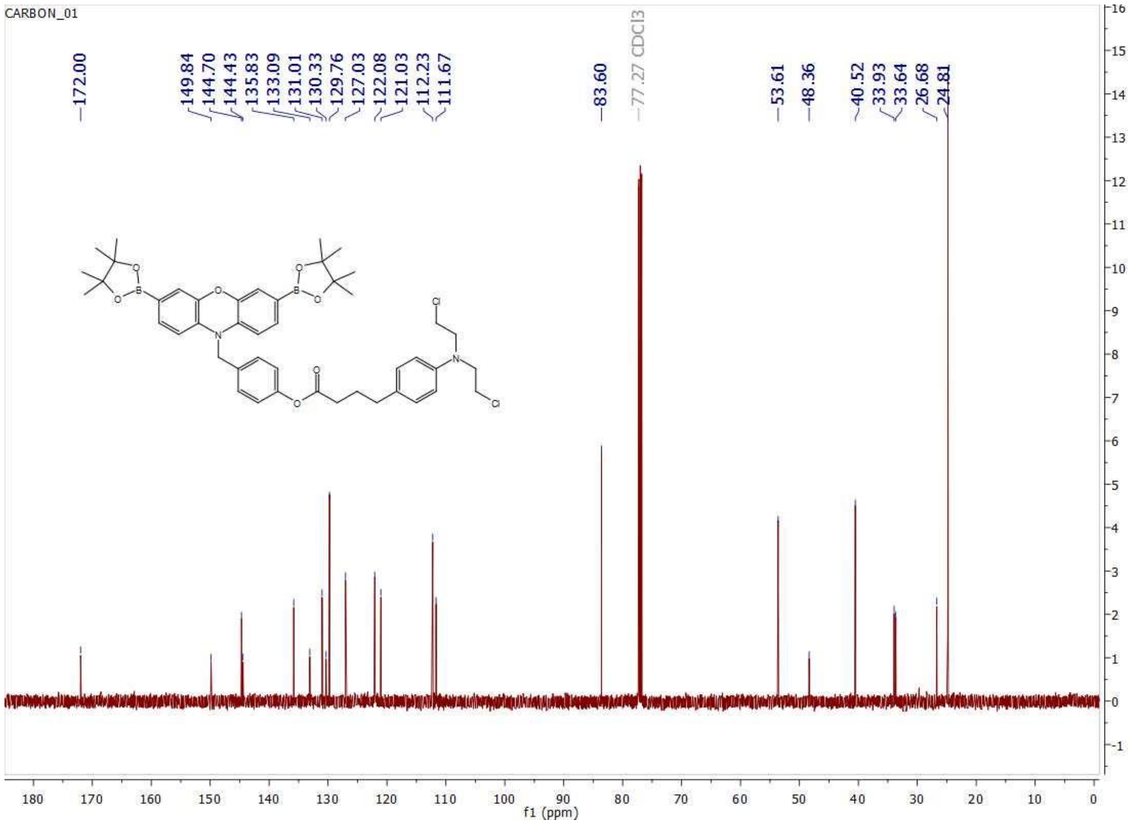

^{13}C NMR of **4-((3,7-bis(4,4,5,5-tetramethyl-1,3,2-dioxaborolan-2-yl)-10*H*-phenoxazin-10-yl)methyl)phenyl 4-(4-(bis(2-chloroethyl)amino) phenyl) butanoate**

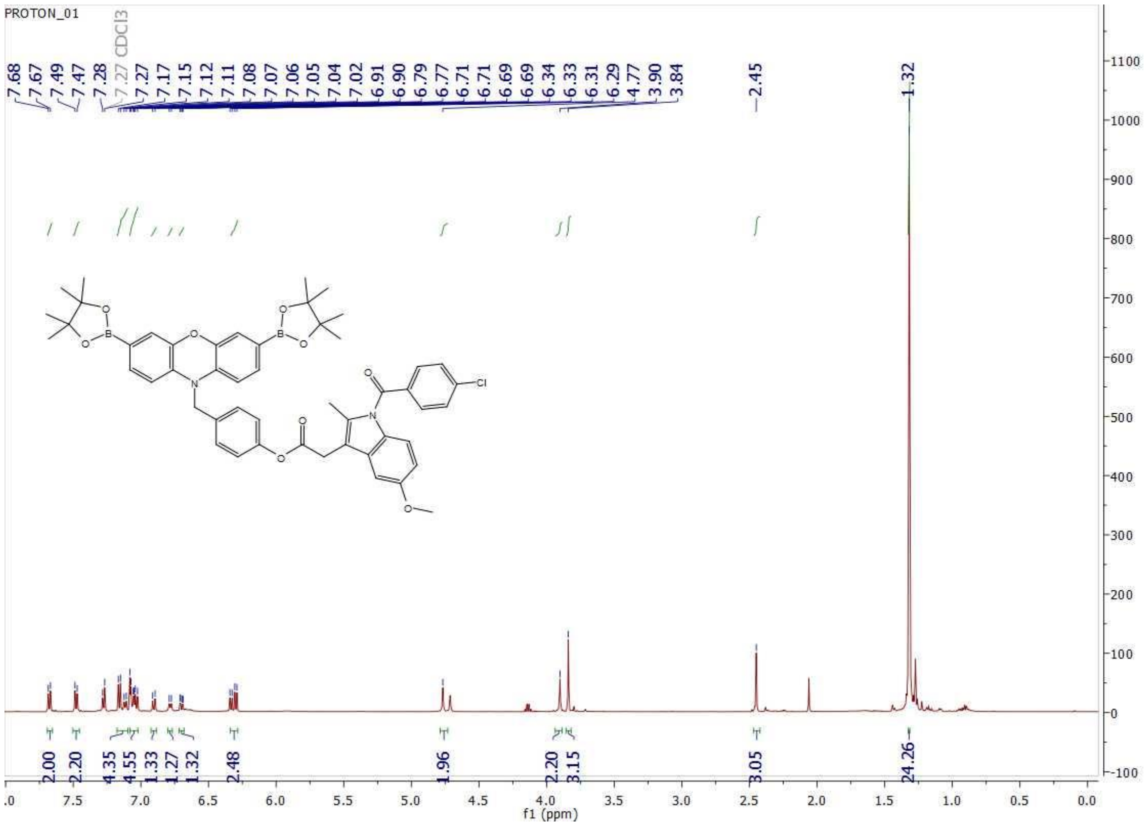

^{1}H NMR of **4-((3,7-bis(4,4,5,5-tetramethyl-1,3,2-dioxaborolan-2-yl)-10*H*-phenoxazin-10-yl)methyl)phenyl 2-(1-(4-chlorobenzoyl)-5-me-thoxy-2-methyl-1*H*-indol-3-yl)acetate**

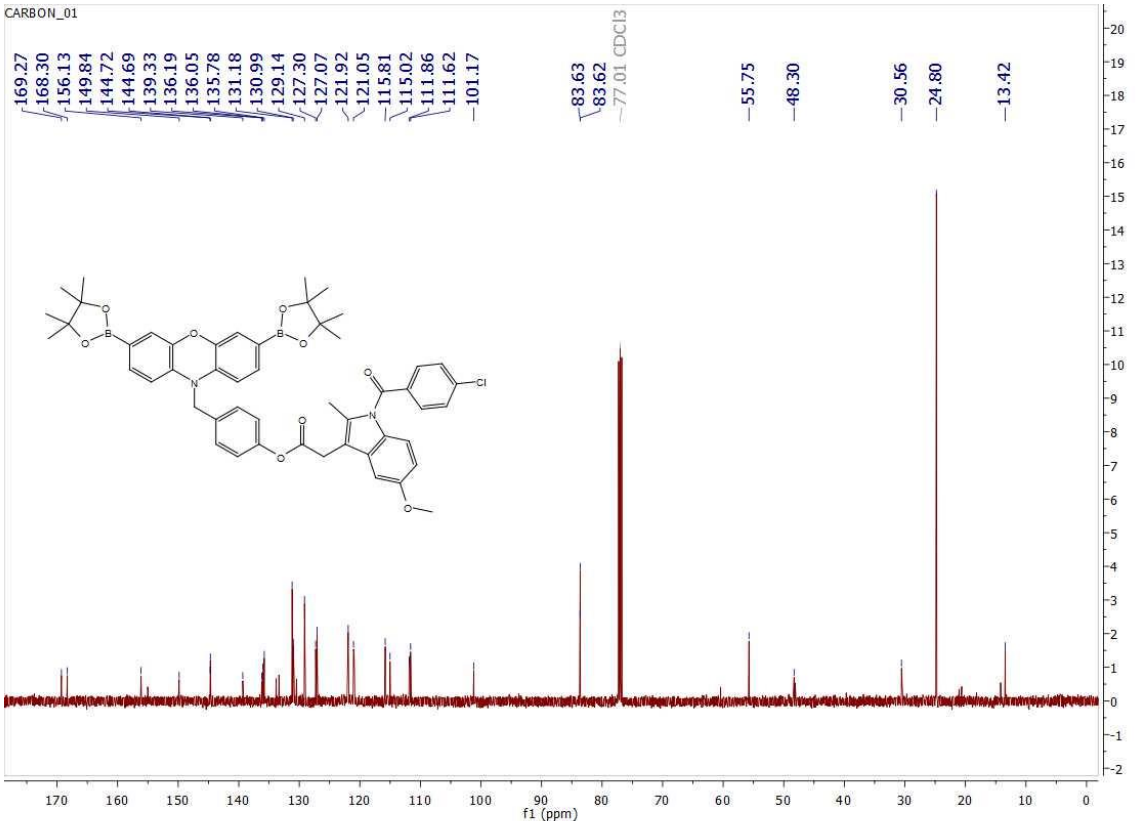

^{13}C NMR of **4-((3,7-bis(4,4,5,5-tetramethyl-1,3,2-dioxaborolan-2-yl)-10*H*-phenoxazin-10-yl)methyl)phenyl 2-(1-(4-chlorobenzoyl)-5-me-thoxy-2-methyl-1*H*-indol-3-yl)acetate**

Author Contributions

M.W. and A.C.S. conceived and designed the research. M.W. conducted the synthesis and fluorescence experiments and carried out data analyses with M.L.O and C.E.F.J. supporting on some of these aspects. H.-H.H. conducted cell experiments. B.L. conducted *in vivo* experiments. Y.Z and J.L supported H.-H.H. and B.L. The manuscript was written by M.W with support from A.C.S., T.D.J, H.-H.H. and X.P.H. S.D.B, A.B.M, and J.L. offered guidance on the project. All authors read and approved the final manuscript.
